# MDT-15/MED15 permits longevity at low temperature via enhancing lipidostasis and proteostasis

**DOI:** 10.1371/journal.pbio.3000415

**Published:** 2019-08-13

**Authors:** Dongyeop Lee, Seon Woo A. An, Yoonji Jung, Yasuyo Yamaoka, Youngjae Ryu, Grace Ying Shyen Goh, Arshia Beigi, Jae-Seong Yang, Gyoo Yeol Jung, Dengke K. Ma, Chang Man Ha, Stefan Taubert, Youngsook Lee, Seung-Jae V. Lee

**Affiliations:** 1 Department of Life Sciences, Pohang University of Science and Technology, Pohang, Gyeongbuk, South Korea; 2 Department of Biological Sciences, Korea Advanced Institute of Science and Technology, Yuseong-gu, Daejeon, South Korea; 3 Department of Integrative Bioscience & Biotechnology, Pohang University of Science and Technology, Pohang, Gyeongbuk, South Korea; 4 Research Division and Brain Research Core Facilities, Korea Brain Research Institute, Daegu, South Korea; 5 Centre for Molecular Medicine and Therapeutics, BC Children's Hospital Research Institute, Department of Medical Genetics, University of British Columbia, Vancouver, British Columbia, Canada; 6 Department of Chemical Engineering, Pohang University of Science and Technology, Pohang, Gyeongbuk, South Korea; 7 School of Interdisciplinary Bioscience and Bioengineering, Pohang University of Science and Technology, Pohang, Gyeongbuk, South Korea; 8 Cardiovascular Research Institute and Department of Physiology, University of California San Francisco, San Francisco, California, United States of America; Buck Institute for Research on Aging, UNITED STATES

## Abstract

Low temperatures delay aging and promote longevity in many organisms. However, the metabolic and homeostatic aspects of low-temperature–induced longevity remain poorly understood. Here, we show that lipid homeostasis regulated by *Caenorhabditis elegans* Mediator 15 (MDT-15 or MED15), a transcriptional coregulator, is essential for low-temperature–induced longevity and proteostasis. We find that inhibition of *mdt-15* prevents animals from living long at low temperatures. We show that MDT-15 up-regulates *fat-7*, a fatty acid desaturase that converts saturated fatty acids (SFAs) to unsaturated fatty acids (UFAs), at low temperatures. We then demonstrate that maintaining a high UFA/SFA ratio is essential for proteostasis at low temperatures. We show that dietary supplementation with a monounsaturated fatty acid, oleic acid (OA), substantially mitigates the short life span and proteotoxicity in *mdt-15(-)* animals at low temperatures. Thus, lipidostasis regulated by MDT-15 appears to be a limiting factor for proteostasis and longevity at low temperatures. Our findings highlight the crucial roles of lipid regulation in maintaining normal organismal physiology under different environmental conditions.

## Abstract 2

Low temperatures delay aging and promote longevity in many organisms. This study shows that at low ambient temperatures, Mediator 15, a transcriptional coregulator, allows the nematode Caenorhabditis elegans to live longer by increasing the levels of unsaturated lipids, helping to maintain protein homeostasis.

## Introduction

Environmental temperature has a major influence on organismal physiology, including growth, metabolism, and aging. The body temperature of poikilothermic organisms, such as *C*. *elegans*, is subject to changes in environmental temperatures; these organisms live long at low ambient temperatures but short at high temperatures [1, 2]. In addition, homeothermic mice with reduced body temperature live long [3], suggesting potentially conserved mechanisms across diverse species. Several genetic factors have been identified to modulate temperature-dependent life-span changes in *C*. *elegans* [4–7]. However, the metabolic processes underlying this phenomenon remain poorly understood.

The relative proportion of unsaturated fatty acids (UFAs) and saturated fatty acids (SFAs) is homeostatically regulated in various organisms [8]. In *C*. *elegans*, the UFA/SFA ratio is inversely proportional to the environmental temperature [9]. This is consistent with the idea that more UFAs than SFAs are required for the maintenance of physical properties of lipids in biological membrane at low temperature [8]. Changes in the UFA/SFA ratio are also important for *C*. *elegans* to adjust its growth to high or low temperatures [10, 11]. However, the mechanisms by which the UFA/SFA ratio regulates the life span of organisms at different environmental temperatures remain largely unknown.

Here, we showed that Mediator 15 (MDT-15/MED15), a subunit of the Mediator complex for RNA-polymerase-II–regulated transcription, was essential for the longevity of *C*. *elegans* at low environmental temperatures. We found that *mdt-15* mutations prevented worms from living long at low temperatures. We showed that *mdt-15* was required for expressing *fat-7*, a fatty acid desaturase crucial for increasing the UFA/SFA ratios at low temperatures. Furthermore, we demonstrated that a homeostatic increase in the UFA/SFA ratios at low temperatures was critical for longevity and proteostasis. These data suggest that MDT-15 is a limiting factor for the low-temperature–mediated longevity of *C*. *elegans* and that it exerts this effect via the maintenance of the physical properties of lipidostasis and proteostasis.

## Results

### *mdt-15* is required for longevity and organismal fitness at low temperatures

**Fig 1:**
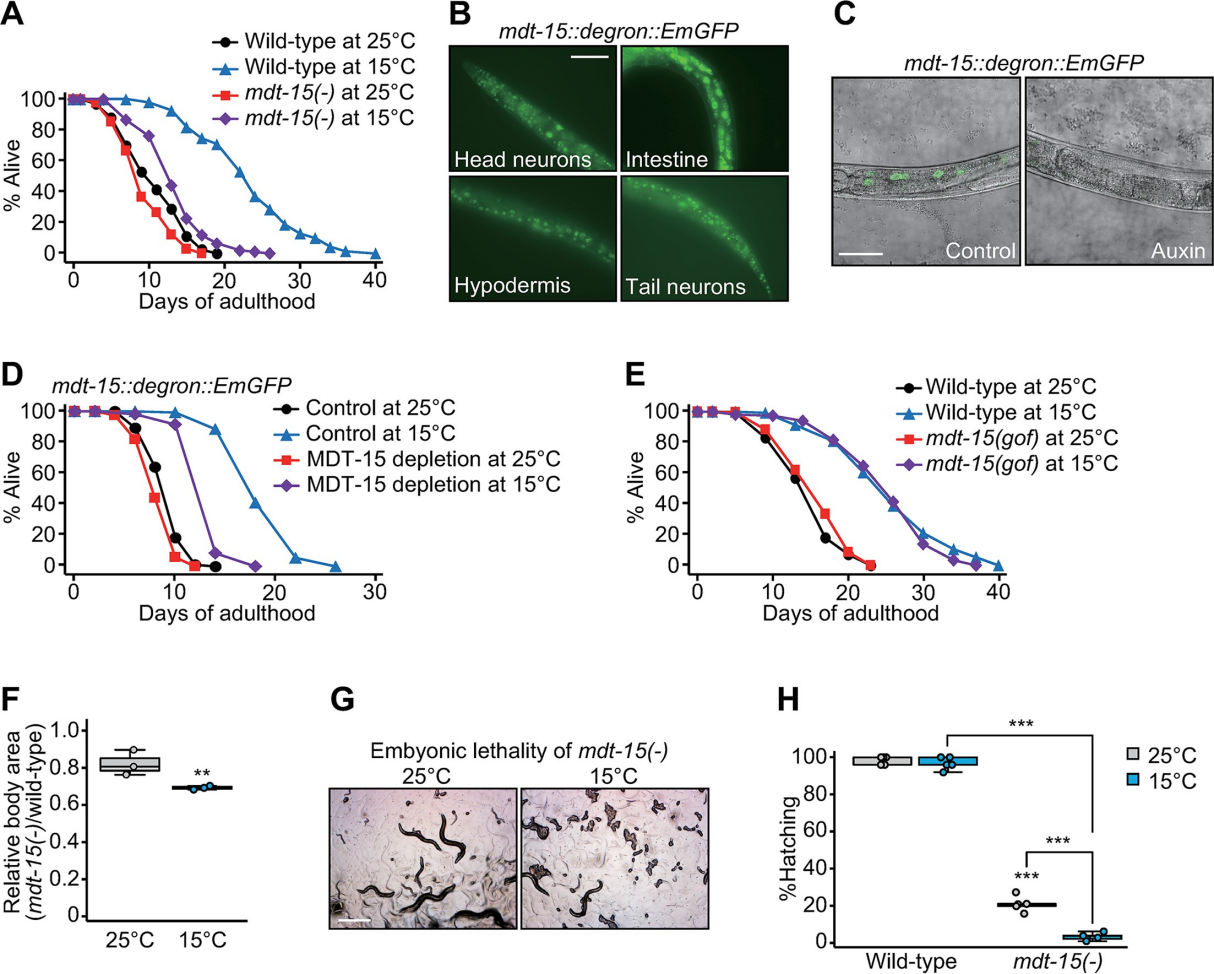
MDT-15 limits the growth and longevity at low temperatures. (**A**) Life span of wild-type and *mdt-15(tm2182)* [*mdt-15(-)*] *C*. *elegans* at 25 °C and 15 °C. The life-span assays in panel A were performed without FUdR, which prevents progeny from hatching. (**B**) Expression of *mdt-15*::*degron*::*EmGFP* generated by CRISPR/Cas9 knock-in in the nuclei of many tissues, including neurons, intestine, and hypodermis. Scale bar: 50 μm. (**C**) Depletion of MDT-15::degron::EmGFP by treatment with auxin at 15 °C was determined by using a confocal microscopy. The worms carry ubiquitously expressed TIR1 (*eft-3p*::*TIR1*::*mRuby*) for the auxin-inducible protein degradation. Scale bar: 50 μm. (**D**) MDT-15 depletion substantially decreased longevity at 15 °C. Control and MDT-15 depletions indicate solvent (ethanol) and auxin treatments, respectively. (**E**) Life-span curves of wild-type and *mdt-15(yh8)* [*mdt-15(gof)*] mutant animals at 25 °C and 15 °C. (**F**) The relative ratio of body areas of *mdt-15(-)* and wild-type animals at 25 °C and 15 °C. The relative ratios were calculated from the same data set shown in S1F Fig. Data points indicate the averages of the ratios from independent experiments. Eight worms were analyzed in each experimental set. Box and whiskers were plotted by using the Tukey method in this and other figures (two-tailed Student *t* tests, *p < 0.05). (**G**) Images show unhatched progeny of *mdt-15(-)* animals at 15 °C. Scale bar: 500 μm. (**H**) Percentage of wild-type and *mdt-15(-)* mutant larvae that hatched at 25 °C and 15 °C. The hatching defect caused by *mdt-15(-)* was also measured at 16 °C (S1G Fig). (*n* ≥ 248 from five independent experiments.) We were able to maintain *mdt-15(-)* worms at low temperatures because the embryonic lethality was not 100%. See S1 and S2 Tables for statistical analysis and additional repeats of the life-span assays, as well as S6 Table for the Cox proportional hazard regression analysis for this and other figures and S7 Table for the standard deviations of box plot data. *eft-3p*, eukaryotic translation elongation factor 3 promoter; *EmGFP*, Emerald green fluorescent protein; FUdR, 5-fluoro-2′-deoxyuridine; *gof*, gain of function; MDT-15, Mediator 15; TIR1, transport inhibitor response 1.

MDT-15 is a subunit of the Mediator complex that regulates diverse physiological aspects, including lipid metabolism, stress resistance, and life span [12–17]. While performing experiments for our previous reports [16, 18], we found that reduction of function *mdt-15* mutations [*mdt-15(-)*] greatly suppressed the long life span of *C*. *elegans* at a low temperature (15 °C) (Fig 1A and S1A and S1B Fig). In contrast, *mdt-15(-)* had a marginal effect on the life span at a high temperature (25 °C) (Fig 1A and S1A and S1B Fig) (*n* = 5). The Cox proportional hazard regression analysis also supports the bigger life-span–shortening effect of *mdt-15(-)* at low temperatures more than at high temperatures (S1A Fig). To confirm this result, we employed an auxin-inducible degron system using CRISPR/Cas9 knock-in [19] and generated *mdt-15*::*degron*::*Emerald green fluorescent protein* (*EmGFP*) strains (Fig 1B). We found that auxin-induced depletion of MDT-15 (Fig 1C and S1C and S1D Fig) substantially suppressed longevity at 15 °C (Fig 1D and S1E Fig) (*n* = 3). Next, we examined the effects of an *mdt-15* gain-of-function (*gof*) mutation [10], which we generated in a wild-type background by using CRISPR/Cas9, on life span at different temperatures. We found that the *mdt-15(gof)* mutations did not affect the life span at 25 °C or 15 °C (Fig 1E) (*n* = 4). Together, these data indicate that MDT-15 is required but not sufficient for increasing the adult life span at low temperatures.

Next, we determined whether *mdt-15* was important for maintaining the overall fitness, including growth and reproduction, of *C*. *elegans* at different temperatures. *mdt-15(-)* mutations elicited a greater impact on growth at low temperatures than at high temperatures (Fig 1F and S1F Fig). In addition, the defects in hatching caused by *mdt-15(-)* mutations were more pronounced at low temperatures than at a high temperature (25 °C) (Fig 1G and 1H and S1G Fig), although the progeny numbers of *mdt-15(-)* mutants were similar at low and high temperatures (S1H Fig). These data suggest that MDT-15 plays a role in longevity and fitness more substantially at low temperatures than at high temperatures. Our results extend the previously reported functions of MDT-15 in life-span regulation [13] to differential roles of MDT-15 in longevity at high and low temperatures.

### MDT-15 regulates the proper expression of fatty acid desaturases at low temperatures

Next, we sought to identify genes whose expression was affected by MDT-15 at different temperatures. Our RNA sequencing (RNA-seq) analysis indicates that 79 genes were up-regulated and 253 genes were down-regulated at 15 °C in an MDT-15–dependent manner (fold change > 1.5 and *p*-values < 0.05; S2A–S2C and S3A–S3F Figs). Gene ontology (GO) analysis showed that GO terms involved in diverse metabolic processes, including aminoglycan, fatty acid, and carboxylic acid metabolism, were enriched among genes up-regulated in an MDT-15–dependent manner at 15 °C (S2D Fig). GO terms, including chromatin assembly, aminoglycan-catabolic process, response to heat, immune system process, and response to unfolded protein, were overrepresented among the MDT-15–dependently down-regulated genes at 15 °C (S2E Fig).

**Fig 2:**
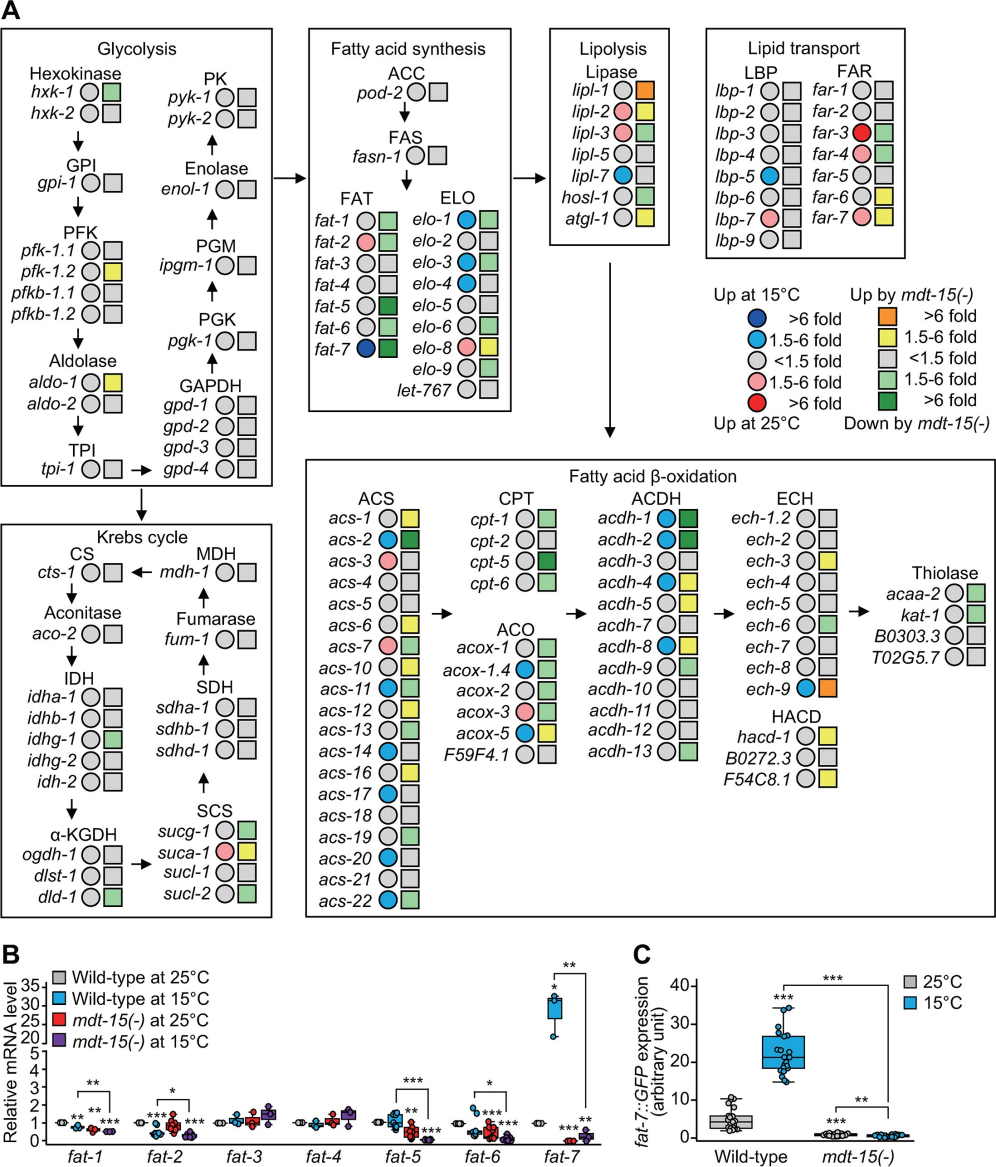
The effects of temperature changes and *mdt-15(-)* mutations on the expression of genes involved in metabolism. (**A**) Simplified carbohydrate and lipid metabolic pathways. Changes in the expression of genes involved in carbohydrate and fatty acid metabolism are shown. Fold changes at 15 °C and by *mdt-15(tm2182)* [*mdt-15(-)*] mutations are shown in circles and squares, respectively. See S3 Table for fold changes and *p*-values as well as S1 Data and GEO database (accession number GSE128815) for RNA-seq data. (**B**) mRNA levels of *fat-1* through *fat-7* in wild-type and *mdt-15(-)* at 25 °C and 15 °C were measured by using qRT-PCR (two-tailed Student *t* tests, *p < 0.05, **p < 0.01, ***p < 0.001, *n* ≥ 3). (**C**) Quantification of *fat-7*::*GFP* fluorescence intensity in wild-type and *mdt-15(-)* animals at 25 °C and 15 °C (two-tailed Student *t* tests, **p < 0.01, ***p < 0.001, *n* ≥ 20 from three independent experiments). Representative images are shown in S3H Fig. See S7 Table for the standard deviations of box plot data. *acaa*, acetyl-CoA acyltransferase; ACC, acetyl-CoA carboxylase; ACDH, acyl-CoA dehydrogenase; ACO, acyl-CoA oxidase; ACS, acyl-CoA synthetase; aldo, aldolase; *atgl*, adipose triglyceride lipase; CPT, carnitine palmitoyl transferase; CS, citrate synthase; ECH, enoyl-CoA hydratase; *elo*, fatty acid elongase; enol, enolase; FAR, fatty-acid–and retinol-binding protein; FAS, fatty acid synthase; FAT, fatty acid desaturase; GAPDH, glyceraldehyde 3-phosphate dehydrogenase; GEO, Gene Expression Omnibus; *GFP*, green fluorescent protein; GPI, glucose-6-phosphate isomerase; HACD, hydroxyacyl-CoA dehydrogenase; *hosl*, hormone-sensitive lipase; *hxk*, hexokinase; IDH, isocitrate dehydrogenase; *ipgm*, cofactor-independent phosphoglycerate mutase; *kat*, 3-ketoacyl-coA thiolase; LBP, lipid-binding protein; *lipl*, lipase-like; MDH, malate dehydrogenase; MDT-15, Mediator 15; qRT-PCR, quantitative reverse-transcription PCR; PFK, phospho-fructo-kinase; PGK, phosphoglycerate kinase; PGM, phosphoglycerate mutase; PK, pyruvate kinase; RNA-seq, RNA sequencing; SCS, succinyl-CoA synthetase; SDH, succinate dehydrogenase; TPI, triosephosphate isomerase; α-KGDH, α-ketoglutarate dehydrogenase.

Based on our GO analysis showing “metabolism” terms enriched among genes up-regulated at 15 °C in an MDT-15–dependent manner, we further analyzed our RNA-seq data with a focus on genes located in carbohydrate and lipid metabolic pathways (Fig 2A and S3 Table). We found that genes that directly affect lipid metabolism, including fatty acid synthesis, lipolysis, lipid transport, and fatty acid *β*-oxidation, tended to be more up- or down-regulated by changes in temperature and *mdt-15(-)* mutation than those involved in glycolysis and the Krebs cycle (Fig 2A and S3 Table). In addition, the expression of many fatty acid desaturases and elongases involved in fatty acid biosynthesis was down-regulated in *mdt-15(-)* mutants, except *fatty acid elongase-8* (*elo-8*) (Fig 2A and S3 Table). In contrast, the expression of genes involved in lipolysis, lipid transport, and fatty acid *β*-oxidation did not show a consistent pattern at different temperatures or by *mdt-15(-)* mutations (Fig 2A and S3 Table). These data suggest that MDT-15 up-regulates the biosynthesis and desaturation of fatty acids at low temperatures.

We noticed that the expression of *fat-7* was the most strongly associated with temperature and MDT-15 genetic background changes (Fig 2A, S3G Fig and S2 Data). Among all seven fatty acid desaturases, the expression level of *fat-7* is significantly higher at 15 °C than at 25 °C [11], and we found that this was MDT-15 dependent (S3G Fig). In contrast, the other six fatty acid desaturase genes did not exhibit MDT-15–dependent changes in expression at different temperatures (S3G Fig). We confirmed the results by using quantiative reverse-transcription PCR (qRT-PCR) assays for *fat-1* to *fat-7* mRNAs and *fat-7*::*GFP* transgenic animals (Fig 2B and 2C and S3H Fig). Together, these data suggest that MDT-15 specifically increases the expression of *fat-7* at low temperatures.

### *mdt-15* mutations decrease the UFA/SFA ratio

**Fig 3:**
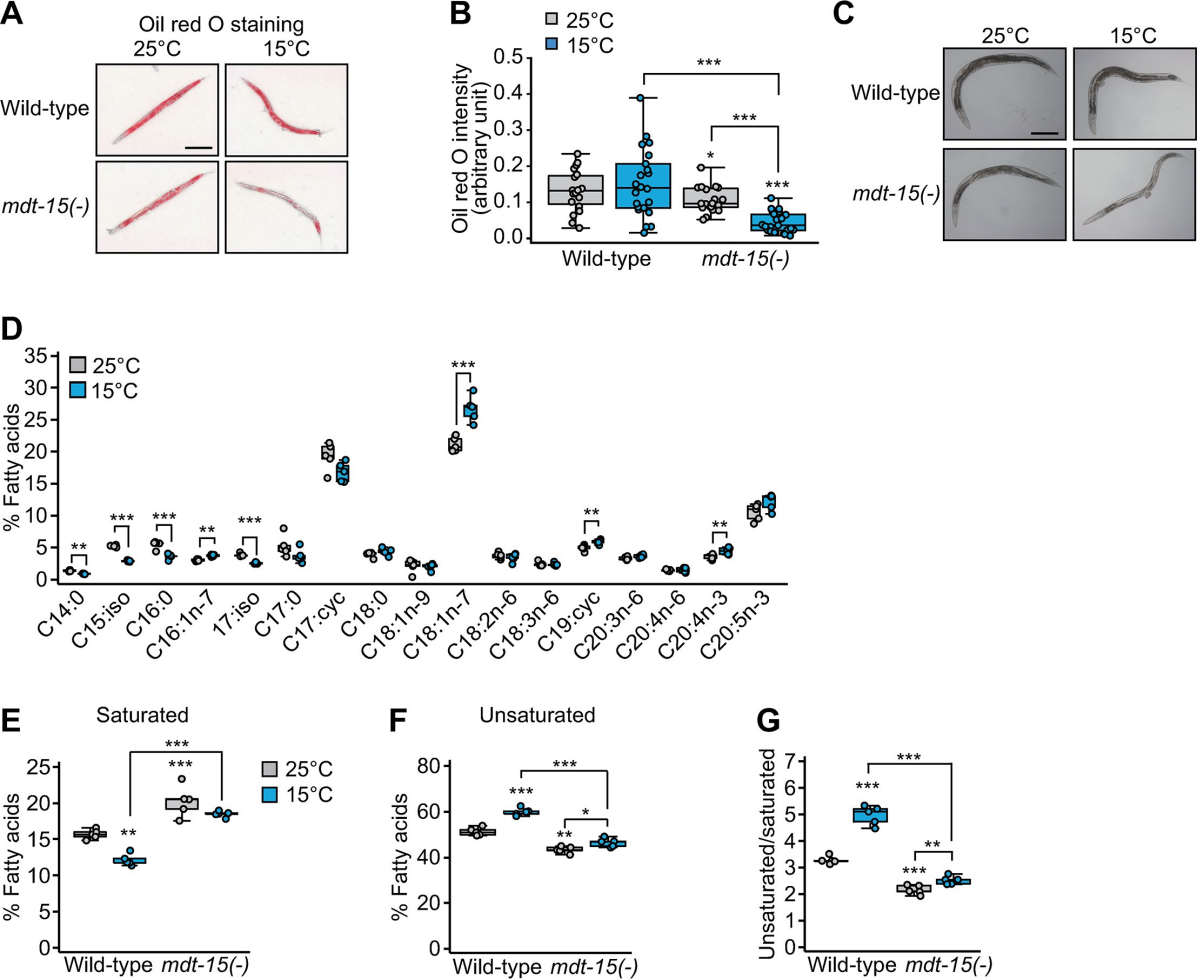
MDT-15 maintains overall fat levels and UFA/SFA ratios at low temperatures. (**A**) Oil red O signals represent overall fat levels in wild-type and *mdt-15(tm2182)* [*mdt-15(-)*] mutant animals at 25 °C and 15 °C. Scale bar: 200 μm. (**B**) Quantification of the Oil red O staining in panel A (two-tailed Student *t* tests, ***p < 0.001, *n* ≥ 20 from three independent experiments). (**C**) Bright-field images of wild-type and *mdt-15(-)* mutant animals at 25 °C and 15 °C. *mdt-15(-)* mutants displayed pale intestines at 15 °C. The images were obtained when the worms reached day 1 adult stage. Scale bar: 200 μm. (**D**) Total fatty acid composition in wild-type animals at 25 °C and 15 °C was analyzed by using GC/MS (*n* = 5). Fractions of each fatty acid in the total fatty acids (mole/mole) were calculated. (**E**, **F**) Fractions of SFAs (**E**) and UFAs (**F**) in wild-type and *mdt-15(-)* mutant animals at 25 °C and 15 °C. These results are consistent with the effect of *mdt-15(-)* on fatty acid composition at 20 °C [42]. (**G**) The ratios of UFAs/SFAs that were calculated from panels E and F (two-tailed Student *t* tests, *p < 0.05, **p < 0.01, ***p < 0.001, *n* = 5). See S7 Table for the standard deviations of box plot data. cyc, cyclo; GC/MS, gas chromatography/mass spectrometry; iso, isomer; MDT-15, Mediator 15; SFA, saturated fatty acid; UFA, unsaturated fatty acid.

Fatty acid desaturases and MDT-15 are crucial for de novo fat synthesis [20]. By using Oil red O staining, we showed that the overall fat levels were not changed at 15 °C in wild-type animals but were greatly reduced in *mdt-15(-)* mutants (Fig 3A and 3B). At 15 °C, *mdt-15(-)* mutants also displayed a pale intestine phenotype (Fig 3C), which correlates with low fat levels [21]. These data suggest that MDT-15 is crucial for maintaining overall fat levels at low temperatures.

We then examined the effects of low temperature and *mdt-15(-)* mutation on the relative proportion of SFAs and UFAs because fatty acid desaturases increase the unsaturated status of fatty acids. The levels of SFAs, including C14:0 and C16:0, were reduced in wild-type animals at 15 °C (Fig 3D); this was consistent with the findings of a previous report [9]. In contrast, the levels of several UFAs, including C16:1n-7, C18:1n-7, and C20:4n-3, were increased at 15 °C (Fig 3D). The overall levels of SFAs were decreased, whereas those of UFAs were increased, in the wild type at 15 °C (Fig 3E and 3F), and this led to significant increases in the UFA/SFA ratio (Fig 3G). Notably, *mdt-15(-)* mutations reduced the effect of low temperature on changes in the SFA and UFA levels (Fig 3E and 3F). This resulted in a reduced UFA/SFA ratio in *mdt-15(-)* mutants, which was more pronounced at 15 °C than at 25 °C (Fig 3G). These data suggest that MDT-15 is crucial for increasing the UFA/SFA ratio at low temperatures.

### Low UFA/SFA ratio suppresses longevity at low temperatures

**Fig 4:**
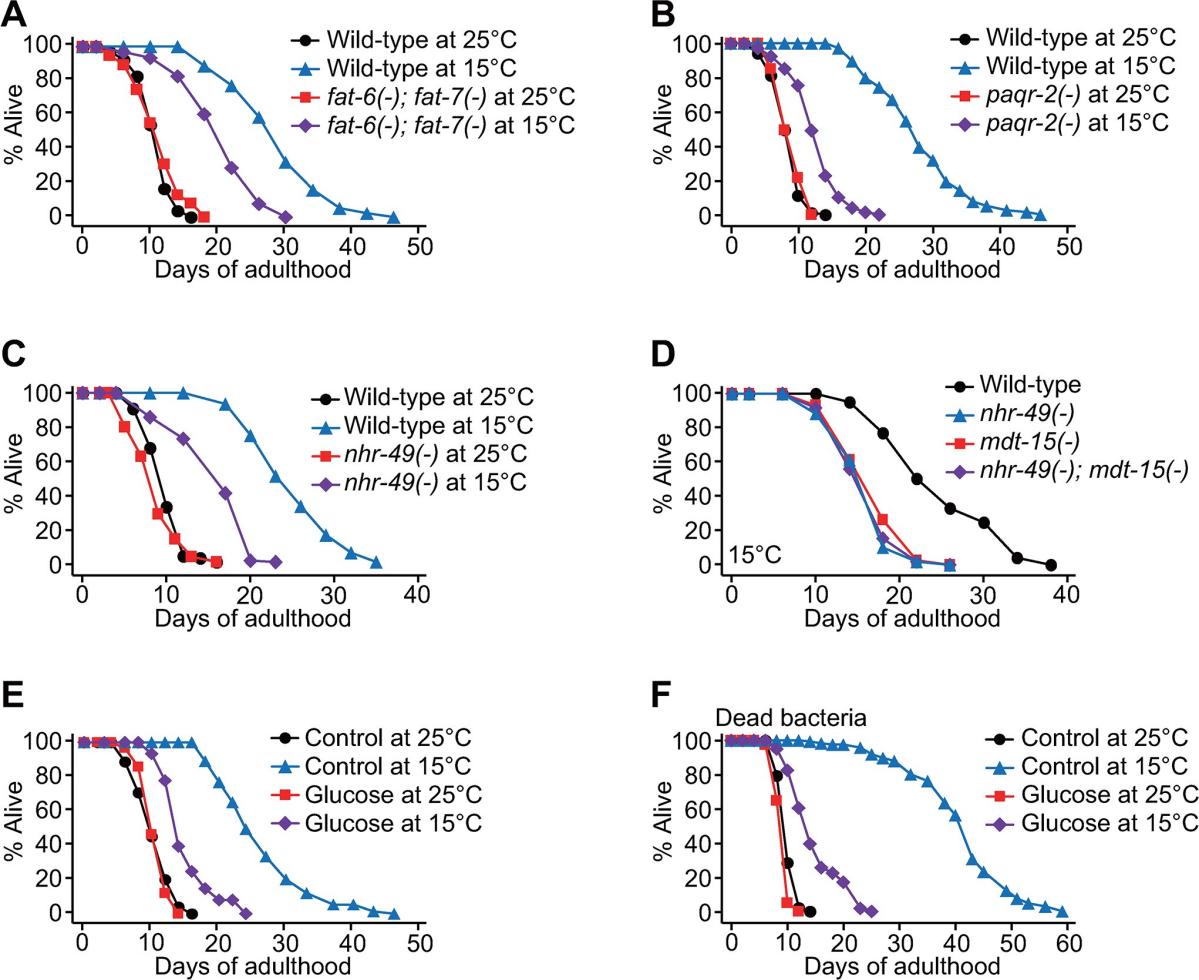
Maintenance of UFA/SFA ratios permits long life span at low temperatures. (**A**) Life-span curves of wild-type and *fat-6(tm331); fat-7(wa36)* [*fat-6(-); fat-7(-)*] mutants at 15 °C and 25 °C. Wild-type and *fat-6(-); fat-7(-)* mutants were cultured at 20 °C until reaching L4 larval stage and then shifted to 15 °C or 25 °C. *mdt-15(tm2182)* [*mdt-15(-)*] mutations also substantially suppressed longevity at 15 °C, when the worms were shifted from 20 °C to 15 °C at L4 larval stage (S1B Fig). (**B**) Life-span curves of wild-type and *paqr-2(tm3410)* [*paqr-2(-)*] animals at 25 °C and 15 °C. Wild-type and *paqr-2(-)* worms were shifted from 20 °C to indicated temperatures at L4 stage. (**C**) Life-span curves of wild-type and *nhr-49(gk405)* [*nhr-49(-)*] animals at 25 °C and 15 °C. (**D**) *nhr-49(-)* mutations did not further shorten the life span of *mdt-15(-)* worms at 15 °C. Genetic inhibition of each of *skn-1* and *sbp-1*, which encode transcription factors that interact with MDT-15 [14, 15, 24], had a small or no effect on the longevity at low temperatures (S4D and S4E Fig). (**E**) Glucose (2%)-enriched diets substantially suppressed the longevity of wild-type animals at 15 °C. (**F**) Life-span curves of worms fed with dead bacteria supplemented with 2% glucose at 25 °C and 15 °C. The worms were shifted from 20 °C to 25 °C or 15 °C at L4 stage. See S1 Table for statistical analysis and additional repeats of the life-span assays and S6 Table for the Cox proportional hazard regression analysis. MDT-15, Mediator 15; *nhr*, nuclear hormone receptor; *paqr*, progestin and adipoQ receptor; *sbp*, sterol regulatory element binding protein; SFA, saturated fatty acid; *skn*, skinhead; UFA, unsaturated fatty acid.

Having established that *mdt-15* is crucial for maintaining overall fat levels and increasing the UFA/SFA ratio at a low temperature, we sought to determine which of these two effects was critical for low-temperature–induced longevity. Several lines of evidence based on genetic and dietary interventions suggest that decreasing the UFA/SFA ratio results in a short life span at low temperatures rather than a reduction in overall fat levels. First, *fat-6(-); fat-7(-)* double mutations, which increase SFA levels [22], significantly and specifically shortened life span at 15 °C but not at 25 °C (Fig 4A). Second, loss of function mutations in the progestin and adipoQ receptor-2 (*paqr-2*), which decrease the expression of *fat-7* and increase the levels of SFAs [10], largely suppressed longevity at 15 °C (Fig 4B). Third, nuclear hormone receptor-49 (*nhr-49*) mutations, which decreased *fat-7* mRNA levels and the UFA/SFA ratio while increasing overall fat levels (S4A–S4C Fig) [23], significantly shortened longevity at 15 °C (Fig 4C). The life span of *nhr-49(-); mdt-15(-)* double mutants at low temperature was similar to that of the single mutants (Fig 4D), suggesting that MDT-15 and NHR-49 act together to contribute to the longevity at low temperature. Fourth, glucose-enriched diets, which decrease the UFA/SFA ratio while increasing the overall fat levels [16, 24, 25], also substantially shortened life span at 15 °C but not at 25 °C (Fig 4E and 4F) [16, 26]. Together, these data suggest that increasing the UFA/SFA ratio, rather than maintaining the overall fat levels, is essential for the longevity of *C*. *elegans* at low temperatures.

### Reduced UFA/SFA ratio induces the expression of cytosolic chaperones at low temperatures

**Fig 5:**
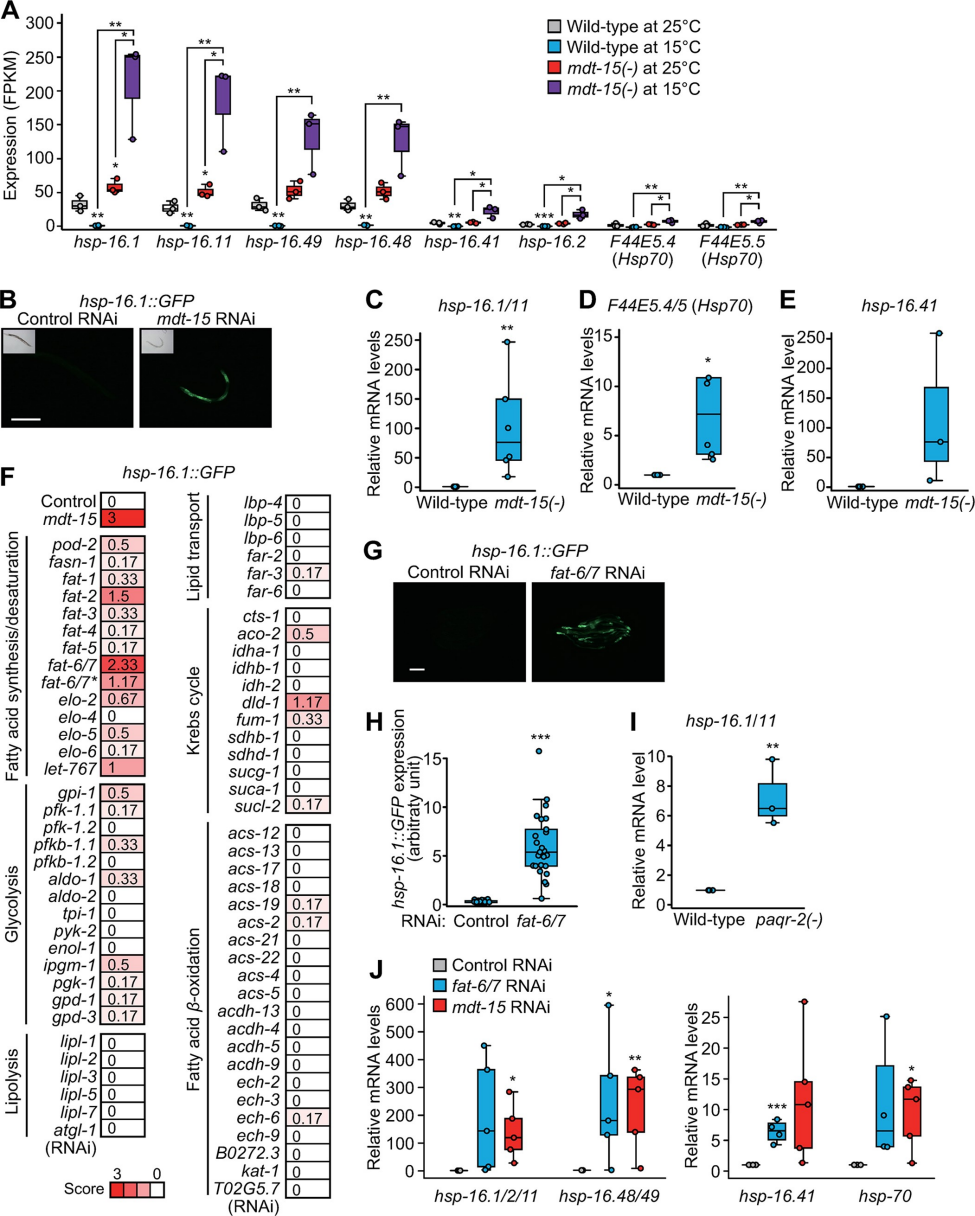
Decreased UFA/SFA ratio induces cytosolic chaperones. (**A**) mRNA levels of selected cytosolic chaperones, *hsp-16*.*1*, *hsp-16*.*11*, *hsp-16*.*49*, *hsp-16*.*48*, *hsp-16*.*41*, *hsp-16*.*2*, *F44E5*.*4*, and *F44E5*.*5* in wild-type and *mdt-15(-)* worms at 25 °C and 15 °C were measured by using RNA-seq (*n* = 3, two-tailed Student *t* tests, *p < 0.05, **p < 0.01). Note that the mRNA levels of *hsp-16*.*1* and *hsp-16*.*11*, those of *hsp-16*.*48* and *hsp-16*.*49*, and those of *F44E5*.*4* and *F44R5*.*5* may not be completely distinguishable because the sequences of these pairs of transcripts are very similar. The induction of some of these chaperone genes by inhibition of *mdt-15* was also observed at 20 °C in a previous report [33]. See S1 Data and GEO database (accession number GSE128815) for RNA-seq data. (**B**) Images of *hsp-16*.*1p*::*hsp-16*.*1*::*GFP* [*hsp-16*.*1*::*GFP*] transgenic worms treated with control RNAi or *mdt-15* RNAi at 15 °C. Scale bar: 200 μm. (**C**) mRNA levels of *hsp-16*.*1*/*11* were measured by using qRT-PCR in wild-type and *mdt-15(tm2182)* [*mdt-15(-)*] animals at 15 °C (*n* = 6). (**D**) The expression of *F44E5*.*4*/*5* at 15 °C was determined by using qRT-PCR (*n* = 5). (**E**) mRNA levels of *hsp-16*.*41* in wild-type and *mdt-15(-)* at 15 °C (*n* = 3). The expression changes were not statistically significant because of large variations. (**F**) A targeted RNAi screen against metabolic genes using *hsp-16*.*1*::*GFP*. Heat maps display average arbitrary scores between 0 to +3 determined by two independent researchers from three different experimental sets. RNAi targeting each of genes encoding various enzymes that regulate fatty acid synthesis/desaturation, glycolysis, lipolysis, lipid transport, Krebs cycle, and fatty acid *β*-oxidation were tested. *fat-6/7* RNAi clones are predicted to target both *fat-6* and *fat-7*, which are 87% identical [74]. Further experimental data using fat-6/7* were excluded based on our qRT-PCR analysis (S5D Fig). (**G**) Representative fluorescence images of *hsp-16*.*1*::*GFP* treated with control RNAi or *fat-6/7* RNAi at 15 °C. Knocking down *fat-6* and *fat-7* increased the level of *hsp-16*.*1*::*GFP*. Scale bar: 200 μm. (**H**) Quantification of the *hsp-16*.*1*::*GFP* treated with control RNAi or *fat-6/7* RNAi at 15 °C. Images of the individual worms were obtained separately for the quantification. (*n* ≥ 23 from three independent experiments). (**I**) *paqr-2(tm3410)* [*paqr-2(-)*] mutations induced the expression of *hsp-16*.*1*/*11* at 15 °C measured by using qRT-PCR (*n* = 3). (**J**) Relative expression of cytosolic chaperone genes by RNAi knock-down of *fat-6/7* and *mdt-15* at 15 °C (*n* ≥ 4, two-tailed Student *t* tests, *p < 0.05, **p < 0.01, ***p < 0.001). The effect of RNAi targeting *fat-6/7* or *mdt-15* on the expression of *fat-6* and *fat-7* are shown in S5D Fig. See S7 Table for the standard deviations of box plot data. ACDH, acyl-CoA dehydrogenase; ACS, acyl-CoA synthetase; *aldo*, aldolase; *atgl*, adipose triglyceride lipase; *cts*, citrate synthase; *dld*, dihydrolipoamide dehydrogenase; ECH, enoyl-CoA hydratase; ELO, fatty acid elongase; *enol*, enolase; FAR, fatty-acid–and retinol-binding protein; FAS, fatty acid synthase; FPKM, fragments per kilobase of exon model per million reads mapped; *fum*, fumarase; GEO, Gene Expression Omnibus; GFP, green fluorescent protein; *gpd*, glyceraldehyde 3-phosphate dehydrogenase; GPI, glucose-6-phosphate isomerase; *hsp*, heat shock protein; IDH, isocitrate dehydrogenase; *ipgm*, cofactor-independent phosphoglycerate mutase; *kat*, 3-ketoacyl-coA thiolase; LBP, lipid-binding protein; *let*, lethal; *lipl*, lipase-like; MDT-15, Mediator 15; *paqr*, progestin and adipoQ receptor; PFK, phospho-fructo-kinase; PGK, phosphoglycerate kinase; *pod*, polarity and osmotic sensitivity defect; *pyk*, pyruvate kinase; qRT-PCR, quantitative reverse-transcription PCR; RNAi, RNA interference; RNA-seq, RNA sequencing; SDH, succinate dehydrogenase; SFA, saturated fatty acid; *suc*, succinyl-CoA ligase; TPI, triosephosphate isomerase; UFA, unsaturated fatty acid.

How does fatty acid composition affect longevity at low temperatures? In our RNA-seq analysis, we noticed that the GO terms “response to heat” and “response to unfolded protein” were enriched among genes down-regulated by low temperature in an MDT-15–dependent manner (S2E Fig). We found that the expression of cytosolic chaperones, such as heat-shock-protein (*hsp*)–encoding genes *hsp-16*.*1*, *hsp-16*.*11*, *hsp-16*.*49*, *hsp-16*.*48*, *hsp-16*.*41*, *hsp-16*.*2*, *F44E5*.*4* (*Hsp70*), and *F44E5*.*5* (*Hsp70*), was reduced at 15 °C in the wild type as expected (Fig 5A). Surprisingly, however, these cytosolic chaperones were highly induced at low temperature (15 °C) compared to high temperature (25 °C) in *mdt-15(-)* mutants (Fig 5A and S2 Data). We confirmed the RNA-seq data by using reporters and qRT-PCR (Fig 5B–5E). In contrast, low temperatures or *mdt-15(-)* mutations had small or no effects on the expression of other proteostasis-related genes, including mitochondrial and endoplasmic reticulum (ER) chaperone, proteasome system, and autophagy genes (S5A–S5C Fig). Together, these data suggest that MDT-15 or MDT-15–mediated processes down-regulate the expression of cytosolic chaperones at low temperatures.

We further determined the connection between *mdt-15*–regulated metabolism and cytosolic chaperone expression at low temperatures because protein folding and homeostasis are crucial for healthy aging and longevity (reviewed in [27]). By performing a targeted RNAi screen against 73 fat- and carbohydrate-metabolism–related genes, we found that knocking down each of several fatty acid synthesis/desaturation genes, including *fat-6* and *fat-7*, substantially increased *hsp-16*.*1*::*GFP* expression (Fig 5F–5H), similar to *mdt-15(RNAi)* (Fig 5B). In addition, RNAi targeting *fat-6* and *fat-7* substantially elevated the expression of several other cytosolic chaperone genes, *hsp-16*.*1/11*, *hsp-16*.*41*, *hsp-16*.*48/49*, and *hsp-70* (Fig 5J). In contrast, genes in other metabolic categories, including *β*-oxidation and lipolysis, had marginal or no effects on *hsp-16*.*1*::*GFP* expression (Fig 5F). Additionally, *paqr-2(-)* mutations, which are expected to decrease UFA/SFA ratio [10], induced the expression of *hsp-16*.*1*/*11* at 15 °C (Fig 5I). Thus, *mdt-15(-)* mutations reduce the expression of *fat-6/7*, which leads to decreases in UFA/SFA ratio, and this in turn appears to induce the expression of cytosolic chaperones at low temperatures.

### Disruption of MDT-15–mediated fatty acid desaturation increases proteotoxicity at low temperature

**Fig 6:**
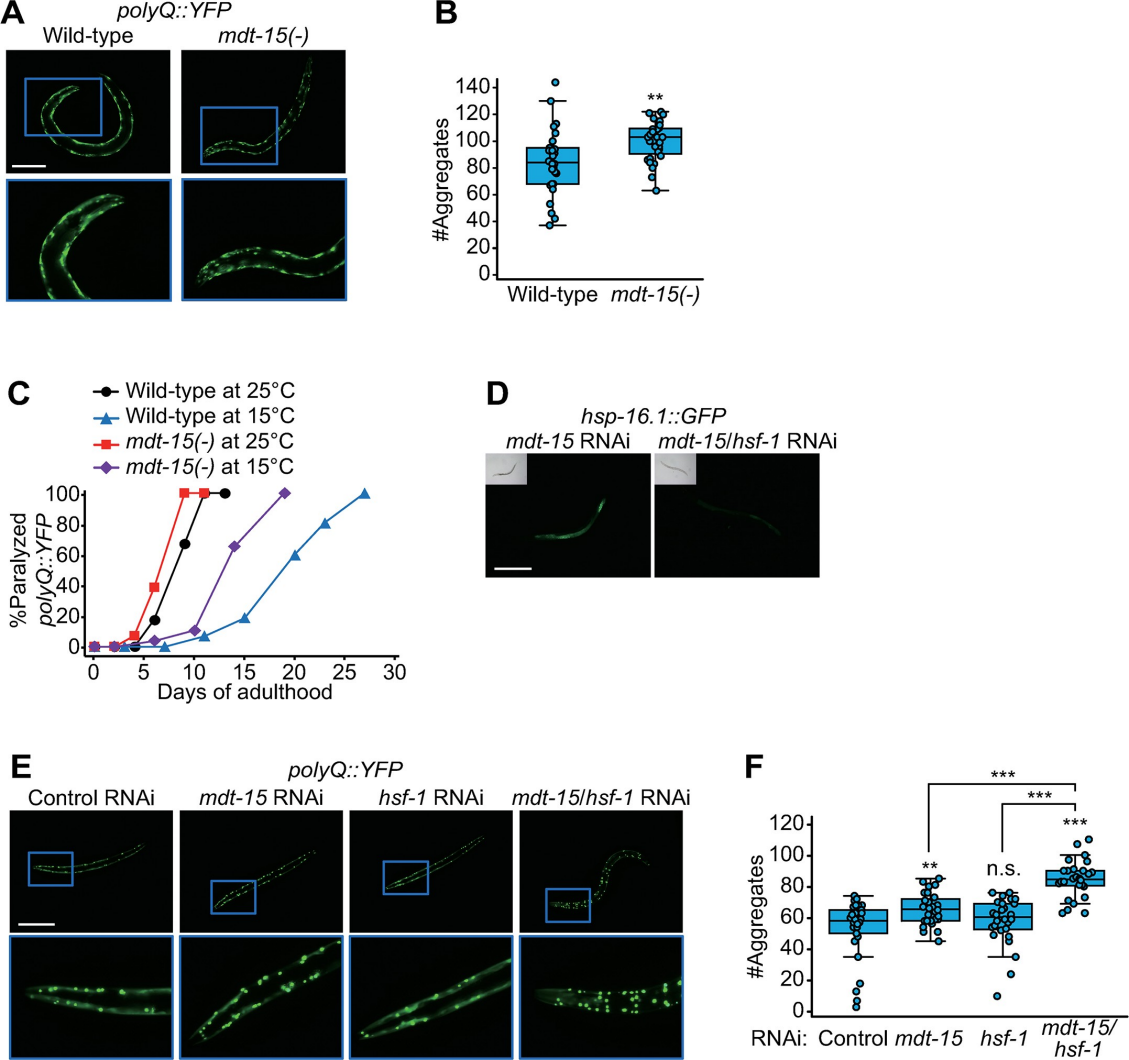
MDT-15 is required for maintaining HSF-1–dependent proteostasis at low temperatures. (**A**) Fluorescence images of *polyQ*::*YFP* (*Q35*::*YFP*) transgenic worms in wild-type and *mdt-15(tm2182)* [*mdt-15(-)*] backgrounds at 15 °C. The images were captured at day 4 adult stage. Scale bar: 200 μm. (**B**) Quantification of the data shown in panel A (*n* ≥ 28 from three independent experiments, two-tailed Student *t* tests, **p < 0.01). These data are also used in Fig 7D for comparison. (**C**) Age-dependent changes in paralyzed *polyQ*::*YFP* transgenic worms in wild-type and *mdt-15(-)* backgrounds at 25 °C and 15 °C. *mdt-15(-)* mutations substantially accelerated the age-dependent paralysis at 15 °C. The transgenic worms in *mdt-15(-)* and wild-type backgrounds were shifted from 20 °C to indicated temperatures at L1 stage. *p*-values were calculated with log-rank test and were smaller than 0.0001 for wild-type versus *mdt-15(-)* animals both at 25 °C and at 15 °C. (**D**) Fluorescence images of *hsp-16*.*1p*::*hsp-16*.*1*::*GFP* [*hsp-16*.*1*::*GFP*] animals treated with *mdt-15* RNAi or *mdt-15*/*hsf-1* double RNAi at 15 °C. Scale bar: 200 μm. (**E**) Images of *polyQ*::*YFP* (*Q40*::*YFP*) transgenic worms treated with *mdt-15* RNAi, *hsf-1* RNAi, or *mdt-15*/*hsf-1* double RNAi at 15 °C. The images were obtained by using L4 stage animals. Scale bar: 200 μm. (**F**) Quantification of the data shown in panel **E** (*n* ≥ 28 from three independent experiments, two-tailed Student’s *t* tests, **p < 0.01, ***p < 0.001). See S5 Table for statistical analysis and additional repeats of the paralysis assays and S7 Table for the standard deviations of box plot data. GFP, green fluorescent protein; HSF-1, heat shock factor 1; *hsp*, heat shock protein; MDT-15, Mediator 15; n.s., not significant; polyQ, polyglutamine; RNAi, RNA interference; YFP, yellow fluorescent protein.

We then asked how *mdt-15(-)* mutations caused the induction of cytosolic-chaperone–encoding genes at low temperatures. We hypothesized that mutations in *mdt-15* decreased protein homeostasis at low temperatures, and this in turn resulted in the induction of the chaperone genes as a response. Consistent with this hypothesis, *mdt-15* mutations significantly increased the number of polyglutamine (polyQ)::yellow fluorescent protein (YFP) puncta (Fig 6A and 6B), a polyglutamine proteotoxicity model in *C*. *elegans* [28]. We also performed age-dependent paralysis assays using the *polyQ*::*YFP* worms [28] and found that the age-dependent paralysis was exacerbated by *mdt-15(-)* mutations at 15 °C (Fig 6C). These results are consistent with our hypothesis that *mdt-15(-)* decreases the protein homeostasis at low temperatures.

Heat shock factor 1 (HSF-1) is a major transcription factor that regulates the expression of cytosolic chaperones [29]. We showed that *hsf-1* RNAi decreased the induction of *hsp-16*.*1*::*GFP* caused by *mdt-15* RNAi at 15 °C (Fig 6D). We also found that *hsf-1* RNAi further increased the number of the polyQ::YFP aggregates in *mdt-15(RNAi)* animals at 15 °C (Fig 6E and 6F). These data suggest that HSF-1–mediated chaperone induction is an adaptive response to increased proteotoxicity caused by the inhibition of *mdt-15* at 15 °C.

### Supplementation with OAs suppresses defects in proteostasis and life span caused by *mdt-15* mutations at low temperatures

**Fig 7:**
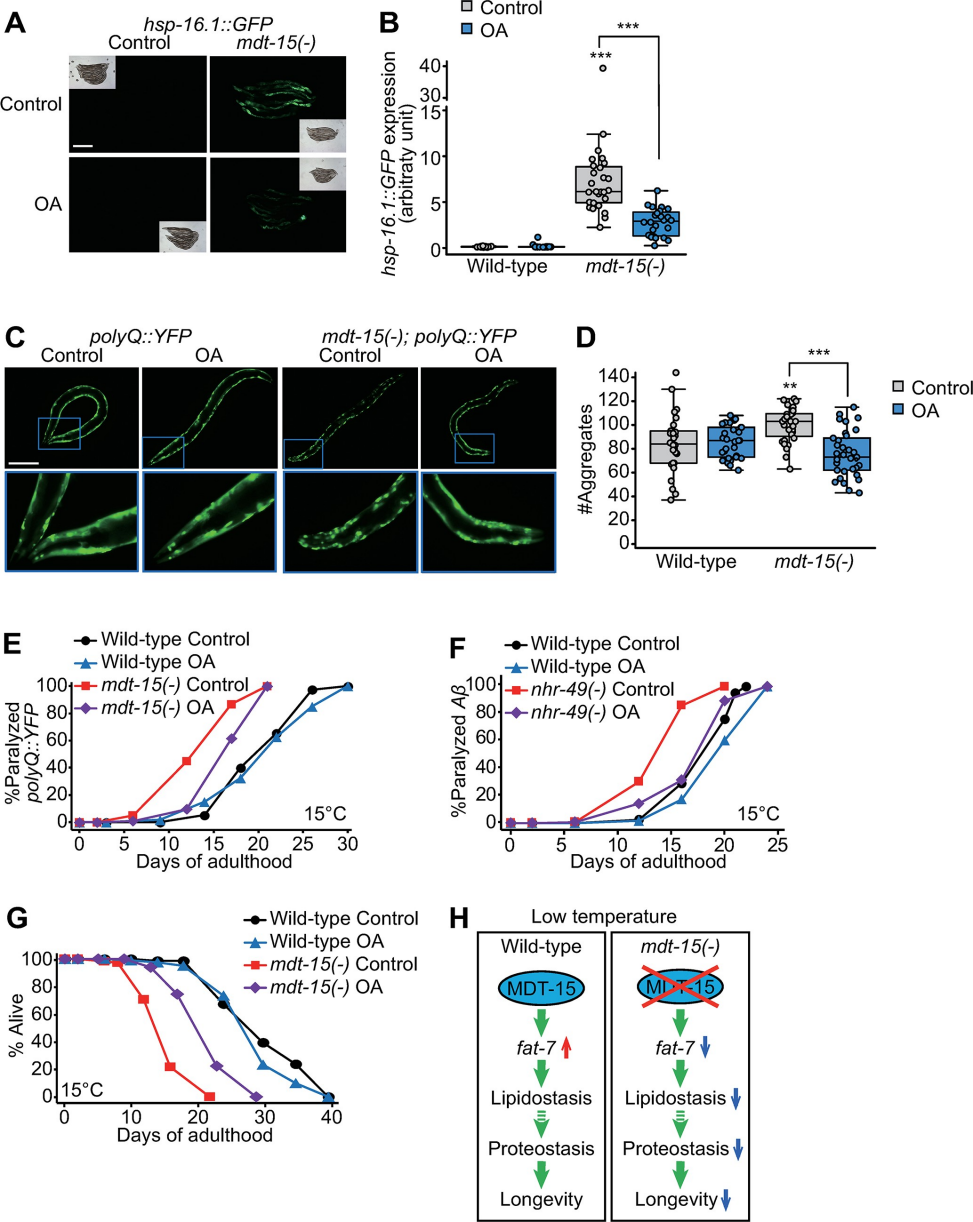
Supplementation of OAs ameliorates proteotoxicity and short life span caused by reduced UFA/SFA ratio at low temperatures. (**A**) Images of *hsp-16*.*1p*::*hsp-16*.*1*::*GFP* [*hsp-16*.*1*::*GFP*] transgenic worms in wild-type and *mdt-15(tm2182)* [*mdt-15(-)*] backgrounds at 15 °C on control or OA-containing plates. Scale bar: 200 μm. (**B**) Quantification of *hsp-16*.*1*::*GFP* treated with OA at 15 °C. Images of the individual worms were obtained separately for the quantification (*n* ≥ 22 from three independent experiments, two-tailed Student *t* tests, ***p < 0.001). See S7 Table for the standard deviations of box plot data in this figure. (**C**) Images of *polyQ*::*YFP* (*Q35*::*YFP*) transgenic worms treated with OA in wild-type and *mdt-15(-)* backgrounds at 15 °C. Scale bar: 200 μm. (**D**) Quantification of the data in panel **C** (*n* ≥ 28 from three independent experiments, two-tailed Student *t* tests, **p < 0.01, ***p < 0.001). The data shown in Fig 6B are used in this panel for comparison. (**E**) Age-dependent increases in the paralysis of *polyQ*::*YFP* transgenic animals in *mdt-15(-)* and wild-type backgrounds at 15 °C. The transgenic worms were fed with OA for their whole life. The paralysis assays were performed 6 times by two independent researchers. Four repeats were performed with synchronized worms from L1 stage, and the other two repeats were performed with worms treated with OA from eggs. For five out of the six repeats, OA substantially suppressed the accelerated age-dependent paralysis in *mdt-15(-)* animals that expressed *polyQ*::*YFP* (S5 Table) (log-rank test, *p* = 0.3895 for wild-type control diet versus wild-type OA, *p* < 0.0001 for *mdt-15(-)* control diet versus *mdt-15(-)* OA). (**F**) Age-dependent increases in the paralysis of *Aβ* transgenic animals in wild-type and *nhr-49(gk405)* [*nhr-49(-)*] backgrounds under control or OA-treated conditions (log-rank test, *p* = 0.091 for wild-type control diet versus wild-type OA, *p* < 0.0001 for *nhr-49(-)* control diet versus *nhr-49(-)* OA). See S5 Table for statistical analysis and additional repeats of the paralysis assays. (**G**) Life-span curves of wild-type and *mdt-15(-)* animals under control or OA-treated conditions at 15 °C. We also showed that OA feeding partially suppressed the defects in the growth of *mdt-15(-)* animals (S6C Fig). See S4 Table for statistical analysis and additional repeats of the life-span assays and S6 Table for the Cox proportional hazard regression analysis. (**H**) A schematic model summarizing the current study. At low temperatures, wild-type MDT-15 up-regulates *fat-7*, which is crucial for maintaining lipidostasis and subsequently proteostasis, which lead to longevity. In *mdt-15(-)* mutants, the expression of *fat-7* is low, and this in turn causes impaired lipidostasis and proteostasis leading to a very short life span at low temperature. GFP, green fluorescent protein; *hsp*, heat shock protein; MDT-15, Mediator 15; *nhr*, nuclear hormone receptor; OA, oleic acid; polyQ, polyglutamine; SFA, saturated fatty acid; UFA, unsaturated fatty acid; YFP, yellow fluorescent protein.

Next, we asked whether the supplementation with an unsaturated fatty acid—oleic acid (OA), which is expected to increase the UFA/SFA ratio [30]—affected proteostasis or life span in *mdt-15(-)* animals at low temperatures. We showed that dietary OA feeding decreased the expression of *hsp-16*.*1*::*GFP* in *mdt-15(-)* mutants (Fig 7A and 7B) and in *mdt-15(RNAi)* worms (S6A and S6B Fig). We also found that OA supplementation suppressed the increased number of puncta as well as enhanced age-dependent paralysis caused by *polyQ*::*YFP* expression in *mdt-15(-)* mutants (Fig 7C–7E). In addition, OA feeding largely suppressed accelerated age-dependent paralysis caused by *nhr-49(-)* mutations in *Aβ* transgenic worms (Fig 7F), a proteotoxicity model for Alzheimer’s disease [31]. Thus, dietary OA supplementation appears to alleviate proteotoxicity in *mdt-15(-)* and *nhr-49(-)* animals by restoring the level of UFA/SFA ratio. Lastly, we found that the OA supplementation substantially lengthened short life span in *mdt-15(−)* mutants at low temperatures (Fig 7G) (*n* = 4). Altogether, our data suggest that the homeostatic up-regulation of UFA/SFA ratio mediated by MDT-15 contributes to enhanced proteostasis and longevity at low temperatures (Fig 7H).

## Discussion

### UFA/SFA ratio limits longevity at low temperatures

MDT-15, a subunit of Mediator complex, regulates lipid metabolism and responses to various stresses in *C*. *elegans* [20]. Consistent with the role of MDT-15 in physiological functions, previous studies demonstrate that lipid metabolism plays important roles in longevity and stress resistance [32]. Our new findings indicate that fatty acid metabolism regulated by MDT-15 is required for the longevity of *C*. *elegans* at low temperatures. When worms were cultured at a low temperature (15 °C), MDT-15 increased the UFA/SFA ratio by increasing the expression of several fatty acid desaturases, including *fat-7*. This homeostatic regulation of lipid metabolism by MDT-15 was essential for the longevity of *C*. *elegans* at low temperatures. Consistent with this model, *mdt-15(-)* mutations reduced the UFA/SFA ratio and suppressed the low-temperature–induced longevity. Similar to *mdt-15(-)* mutations, *fat-6(-); fat-7(-)*, *paqr-2(-)* and *nhr-49(-)* mutations, and glucose-enriched diets decreased longevity at low temperatures. The reduction in the UFA/SFA ratio at low temperatures led to proteotoxicity, resulting in shortened life span. Previous studies established the functional importance of fatty acid metabolism in longevity and stress resistance [32]. MDT-15, a subunit of the Mediator complex, regulates lipid metabolism and responses to xenobiotic, pathogenic, and metal exposure in *C*. *elegans* [13–15, 33–35]. Combined with the roles of lipid metabolism and MDT-15 in various physiological processes [20], our new findings extend the role of fatty acid composition in life-span regulation by showing that *mdt-15* is critical for the longevity of *C*. *elegans* at low temperatures.

### MDT-15 regulates life span in response to changes in temperature and diet

*C*. *elegans* MDT-15 is a transcriptional coregulator crucial for physiological responses to ingested materials, including food, toxins, pathogens, and abiotic stressors [13–15, 33–35]. We previously reported that MDT-15–regulated lipid metabolism is critical for maintaining normal life span in glucose-enriched nutrient conditions [16]. In this study, we demonstrated that MDT-15 was important for longevity at low ambient temperatures. Together, these findings suggest that MDT-15 plays a key role in adapting to changes in environmental temperatures and responding to various ingested materials, including high-glucose diets, toxins, heavy metals, and pathogens. How MDT-15 differentially incorporates various inputs to exert proper cellular and physiological responses currently remains elusive. Because MDT-15 is a subunit of the Mediator complex that interacts with diverse transcription factors [12], it seems plausible that MDT-15 binds to these transcription factors differently under different conditions.

### Specific transcription factors acting with MDT-15 may regulate the temperature-dependent expression of *fat-7*

Several transcription factors—including NHR-49, NHR-45, sterol regulatory element binding protein (SREBP)/SBP-1, Forkhead box O (FOXO)/dauer formation-16 (DAF-16), and HSF-1—regulate transcriptomic changes together with MDT-15 [13, 14, 17, 36, 37]. Therefore, some of these transcription factors potentially act with MDT-15 to modulate low-temperature–induced longevity by regulating the expression of fatty acid desaturases. Among the seven *C*. *elegans* fatty acid desaturases, the expression of *fat-7* was down-regulated at high temperature instead of being up-regulated at low temperature in an MDT-15–dependent manner (Fig 2B and S3G Fig). This raises a possibility that the activity of NHR-49, SBP-1, DAF-16, or HSF-1, which induces *fat-7* [13, 14, 36, 37], is reduced at high temperatures. The effect of temperature on the activity of these transcription factors has not been investigated, except for the heat-activated HSF-1. However, HSF-1 positively regulates the expression of *fat-7* at high temperatures [37], and this is in contrast to the data shown in this study. We speculate that MDT-15 plays more prominent roles than HSF-1 in the temperature-dependent expression changes of *fat-7*. A study has shown that acyl-CoA dehydrogenase (ACDH)-11, whose expression is up-regulated at high temperatures, negatively regulates *fat-7* via NHR-49 [11]. Therefore, it seems possible that inhibition of ACDH-11 mediates up-regulation of *fat-7* via MDT-15 at low temperature, and it will be important to test the physiological role of ACDH-11 focusing on the relationship with NHR-49, MDT-15, and FAT-7.

Because MDT-15 directly interacts with NHR-49 and shares many target genes, including *fat-7* [13], NHR-49 is likely to be the transcription factor that acts with MDT-15 for regulating the temperature-dependent expression of *fat-7*. Consistent with this, *nhr-49(-)* mutations suppressed the longevity at a low temperature similar to *mdt-15(-)* mutations, and *nhr-49* and *mdt-15* appear to act in the same pathway for the longevity at low temperatures (Fig 4C and 4D) [11, 13].

### UFA/SFA ratio may influence low-temperature–induced longevity via tuning membrane fluidity

The UFA/SFA ratio is crucial for maintaining the membrane fluidity for adaptation to different temperatures [8]. In *C*. *elegans*, the disruption of membrane fluidity leads to developmental defects at low temperatures and resistance against heat (37 °C) stress [11, 38]. Here, we showed that *mdt-15(-)* mutations decreased the UFA/SFA ratio at low temperature and suppressed the longevity. We also found that various genetic interventions such as mutations in the fatty acid desaturases, *nhr-49*, and *paqr-2*, as well as glucose-enriched diets, all of which decrease the UFA/SFA ratio, shortened life span specifically at low temperatures. Thus, a reduction in the UFA/SFA ratio at low temperatures may increase the rigidity of membranes to a harmful level, which may in turn shorten life span. A recent study has demonstrated that feeding worms with glucose or glycolysis metabolites increases their membrane rigidity by altering bacterial metabolism [25]. However, we showed that feeding *C*. *elegans* with glucose along with dead bacterial diets also substantially shortened life span at low temperature (Fig 4F). Therefore, glucose-rich diets appear to directly decrease the life span of *C*. *elegans* at low temperatures independently of bacterial metabolism.

### MDT-15 regulates proteostasis independently of mitochondrial stress

Mitochondrial stress, or a mild disruption of mitochondrial functions, up-regulates the expression of cytosolic chaperones [39, 40]. Therefore, it is possible that the induction of cytosolic heat shock proteins in *mdt-15(-)* mutants observed in this study implicates mitochondrial stress. However, several lines of evidence are against this possibility. First, we found that *mdt-15(-)* mutations had a small or no effect on the induction of mitochondrial stress response genes. Second, unlike the increase in fat levels required for the induction of cytosolic chaperones under mitochondrial stress [39], we showed that *mdt-15(-)* mutations reduced overall fat levels while increasing cytosolic chaperone levels. Third, different from mitochondrial perturbations that improve cytosolic proteostasis [39, 40], we found that the inhibition of *mdt-15* disrupted the proteostasis. Overall, our data suggest that the induction of cytosolic chaperones indirectly occurs as a physiological response to increased proteotoxicity caused by *mdt-15(-)* mutations and is distinct from mitochondrial stress. A previous paper reported several negative regulators of the heat shock response, including *mdt-15* [41], and therefore it will be interesting to identify factors that mediate chaperone gene induction in *mdt-15(-)* at low temperature.

### MDT-15 possibly maintains cytosolic proteostasis via modulating lipidostasis of membrane at low temperature

Several previous reports have suggested the link between membrane fluidity and proteostasis. *mdt-15* mutation triggers ER unfolded protein response (UPR^ER^) by reducing ER membrane lipid desaturation at 20 °C [42]. However, we found that at a low temperature (15 °C), the expression of UPR^ER^ effectors such as *hsp-4* and *endoplasmin-1* (*enpl-1*) was not substantially increased by *mdt-15(-)*. We speculate that *mdt-15(-)* influences cytosolic proteostasis at low temperatures in an ER-homeostasis–independent manner. Consistent with this idea, a study using yeast reported that lipid droplets are crucial for the clearance of cytosolic inclusion bodies, which contain misfolded protein aggregates [43]. FAT-7 is required for the formation of large lipid droplets by desaturating membrane phospholipids in *C*. *elegans* [44]. Therefore, down-regulation of *fat-7* in *mdt-15(-)* animals may lead to defects in the formation of lipid droplets and may subsequently impair cytosolic proteostasis because of defects in the clearance of protein aggregates.

### Low UFA/SFA ratios are associated with various human diseases

Recent reports indicate that supplementing with certain types of UFAs is sufficient to extend the life span of *C*. *elegans* [30, 45]. Together with our current report, these findings suggest the beneficial effects of UFAs on healthy aging. Interestingly, low UFA/SFA ratios are associated with many diseases such as Niemann-Pick disease, hypertension, heart disease, and Alzheimer’s disease [46–49]. Human fibroblasts that carry mutations in Niemann-Pick C1 (*NPC1*), whose mutations are responsible for the Niemann-Pick type C disease, display high levels of SFAs and a reduced membrane fluidity [49]. Low UFA/SFA ratios also positively correlate with heart disease incidences in humans [48]. Spontaneously hypertensive rats show a lower UFA/SFA ratio in the aorta and kidney than normotensive rats [47]. Patients of Alzheimer’s disease display very high levels of SFAs in their brains [46], and this is consistent with our data showing the occurrence of proteotoxicity due to low UFA/SFA ratios. These findings point toward the importance of low UFA/SFA ratios in the pathophysiology of various diseases. Further research on MDT-15 and lipid metabolism using *C*. *elegans* may provide key information that may eventually help the treatment of human diseases because functions of the Mediator complex are evolutionarily well conserved [12].

### Lipidostasis is crucial for longevity

Homeostatic regulation of proteins and DNA is important to prevent premature aging and to promote longevity [50]. In addition, recent studies indicate that RNA quality control mediated by nonsense-mediated mRNA decay and accurate mRNA splicing contributes to longevity in *C*. *elegans* [51–54]. Our current work suggests that lipidostasis is also crucial for longevity at low temperatures, perhaps because lipids are very susceptible to changes in environmental temperatures. We further showed that the disruption of lipid composition at low temperatures increased proteotoxicity and shortened life span, although further mechanistic details need to be clarified. The homeostatic regulation of lipids and proteins appears to be interconnected and tightly regulated for health and longevity, and it will be interesting to investigate the relationship at the molecular level in future research.

## Materials and methods

### Strains

All strains were maintained on nematode growth medium (NGM) agar plates seeded with *Escherichia coli* (OP50). The following strains were used in this study: N2 wild type, IJ235 *mdt-15(tm2182) III* obtained by outcrossing XA7702 [33] four times to Lee lab N2, IJ1648 *ieSi57[eft-3p*::*TIR1*::*mRuby*::*unc-54 3′UTR; cb-unc-119] II* outcrossed four times to Lee lab N2, IJ1651 *yh44[mdt-15*::*degron*::*EmGFP] III* outcrossed four times to Lee lab N2 after CRISPR/Cas9 editing, IJ1729 *ieSi57[eft-3p*::*TIR1*::*mRuby*::*unc-54 3′UTR; cb-unc-119] II; yh44[mdt-15*::*degron*::*EmGFP] III* obtained by crossing IJ1648 and IJ1651, IJ1468 *mdt-15(yh8) III* outcrossed four times to Lee lab N2 after CRISPR/Cas9 editing, DMS303 *nIs590[fat-7p*::*fat-7*::*GFP] V*. IJ1742 *mdt-15(tm2182) III; nIs590[fat-7p*::*fat-7*::*GFP] V* obtained by crossing IJ235 and DMS303, IJ511 *fat-6(tm331) IV; fat-7(wa36) V* outcrossed seven times to Lee lab N2, IJ666 *paqr-2(tm3410) III* obtained by outcrossing QC121 (a gift from the Marc Pilon lab, University of Gothenburg, Göteborg, Sweden) four times to Lee lab N2, CF2774 *nhr-49(gk405) I* outcrossed four times to N2, IJ360 *nhr-49(gk405) I; mdt-15(tm2182) III* obtained by crossing CF2774 and IJ235, IJ1625 *skn-1(zj15)* outcrossed four times to Lee lab N2, LJ300 *In300 [hsp-16*.*1p*::*hsp-16*::*GFP + rol-6D]* (a gift from the Junho Lee lab, Seoul National University, Seoul, South Korea), IJ1811 *mdt-15(tm2182) III; In300 [hsp-16*.*1p*::*hsp-16*::*GFP + rol-6D]* obtained by crossing LJ300 and IJ235, IJ184 *rmIs133[unc-54p*::*Q40*::*YFP]* outcrossed four times to Lee lab N2, IJ1740 *mdt-15(tm2182) III*; *rmIs133[unc-54p*::*Q40*::*YFP]* obtained by crossing IJ235 and IJ184, AM140 *rmIs132[Q35*::*YFP] I*, IJ1809 *rmIs132[Q35*::*YFP] I; mdt-15(tm2182) III* obtained by crossing AM140 and IJ235, CL2006 *dvls2[pCL12(unc-54/human Abeta1-42 minigene); rol-6D]*, and IJ744 *nhr-49(gk405) I; dvls2[pCL12(unc-54/human Abeta1-42 minigene); rol-6D]* obtained by crossing CF2772 and CL2006.

### CRISPR/Cas9 genome editing

*mdt-15*::*degron*::*EmGFP* knock-in was generated as described previously with modifications [19]. The *degron*::*EmGFP* repair template with homology arms (37 bp for 5′ and 36 bp for 3′) was amplified by PCR using pLZ29 as a template [19], and the PCR products were purified by using a PCR purification kit (QIAGEN, Hilden, Germany). Wild-type young (day 1) adult worms were injected with pRF4 (roller injection marker, 50 ng/μl), Cas9 protein (250 ng/μl; Integrated DNA Technologies, Coralville, IA, USA), tracrRNA (100 ng/μl; Integrated DNA Technologies), *mdt-15* crRNA (56 ng/μl, 5′-ATAATCTTAACTTGTAAGTT-3′), and the purified *degron*::*EmGFP* repair template (450 ng/μl). Individual worms were subsequently transferred to new plates, and roller and nonroller F1 worms from plates that contained many rollers were genotyped by using PCR to identify worms that contained knock-in mutations. The *mdt-15*::*degron*::*EmGFP* knock-in was confirmed by sequencing, and the strain was outcrossed four times to Lee lab N2 to remove potential background mutations. For generating a gof *mdt-15(yh8)* mutation [10] in the wild-type background, CRISPR/Cas9 plasmids targeting *mdt-15* were generated by replacing the *pha-1* sgRNA sequence in pJW1285 [55] with *mdt-15* sgRNA sequence (5′-TTTCTTGCCTGAGCTGATGT-3′). Wild-type adults were injected with pRF4 (roller injection marker, 50 ng/μl), pIJ285 (CRISPR/Cas9 plasmid targeting *mdt-15*, 50 ng/μl), and repair template (5′-ATCGAGCTCCTGTGCCTCCAGATCCACAACTAACATCAGCTCAGGCAAGAAATCCACCTGTTACCGTAGCA-3′, 10 ng/μl), and individual worms were transferred to new plates. F1 roller worms were genotyped by PCR to identify the *mdt-15(yh8)* mutation. The *mdt-15(yh8)* mutation was confirmed by using sequencing, and the *mdt-15(yh8)* mutant worms were outcrossed four times to wild-type worms to remove potential nonspecific mutations.

### Life-span assays

Life-span assays were performed as described previously with some modifications [56]. Synchronized worms from eggs were cultured on the OP50-seeded NGM plates at different temperatures and then transferred to plates containing 5 to 10 μM FUdR (Sigma-Aldrich, St. Louis, MO, USA) at the young adult stage (day 1) to prevent their progeny from hatching. The worms were transferred to fresh plates every other day until they stopped producing progeny for life-span assays without FUdR treatment. Auxin-inducible degron assays were performed as described [19]. Briefly, synchronized worms were cultured on OP50-seeded NGM plates at 25 °C or 15 °C until reaching day 1 adult stage and then transferred to new OP50-seeded NGM plates containing 1 mM auxin (indole-3-acetic acid; Alfa Aesar, Haverhill, MA, USA). Ethanol (solvent) was used as a control for auxin treatments. For temperature shift assays, worms were cultured at 20 °C until reaching L4 stage and subsequently shifted to 25 °C or 15 °C. When the worms reached the young adult stage (day 1), the worms were transferred to plates containing FUdR. For glucose-enriched diet experiments, 2% glucose (DAEJUNG, Seoul, South Korea) was added to NGM as described previously [57]. OP50 bacteria that were cultured overnight in liquid LB media were concentrated 20 times by centrifugation and seeded onto NGM plates containing 10 μg/ml kanamycin (Sigma-Aldrich) for life-span assays with dead bacteria, as previously described [57]. For RNAi experiments, bacteria that express double-stranded RNA targeting a specific gene were cultured in liquid LB containing 50 μg/ml ampicillin (USB, Cleveland, OH, USA) at 37 °C overnight and then seeded onto NGM containing 50 μg/ml ampicillin. The RNAi-bacteria–seeded plates were incubated at 37 °C overnight and treated with 1 mM isopropyl β-D-1-thiogalactopyranoside (IPTG; GoldBio, St. Louis, MO, USA) to induce double-stranded RNA at room temperature. Dead worms were determined for the worms that displayed no response upon gently touching with a platinum wire. Animals that crawled off the plates, ruptured, bagged, or burrowed were censored but included in the statistical analysis.

### Body size assays

Wild-type and *mdt-15(tm2182)* worms were cultured on the OP50-seeded high-growth (HG) NGM plates at 20 °C and synchronized by using a bleaching method [58]. The bleached eggs were kept in M9 buffer at 20 °C overnight, and then hatched L1 worms were cultured on OP50-seeded NGM plates at 25 °C or at 15 °C. After developing into fully grown adults (72 hrs at 25 °C and 144 hrs at 15 °C), the worms were placed on a 2% agarose pad and anesthetized by using 100 mM sodium azide (Sigma-Aldrich). For the measurement of body sizes of OA-fed worms, synchronized wild-type and *mdt-15(tm2182)* worms were cultured on control or OA-containing NGM plates from eggs at 25 °C or at 15 °C. Day 1 adult worms were placed on a 2% agarose pad and anesthetized by using 100 mM sodium azide (Sigma-Aldrich). Bright-field images were captured using an AxioCam HRc (Zeiss, Oberkochen, Germany) camera attached to a Zeiss Axioscope A.1 microscope. ImageJ [59] was used for the quantification of body areas.

### Reproduction assays

Wild-type and *mdt-15(tm2182)* worms were cultured at 25 °C or 15 °C at least for two generations prior to the assays. Individual L2 or L3 stage larvae were transferred onto new individual OP50-seeded NGM plates and kept at 25 °C or 15 °C. Twenty wild-type and 30 *mdt-15(−)* mutant worms at each temperature were used for one experimental set, and the assays were repeated five times. When the worms were producing eggs, for 2–3 days at 25 °C and 4–5 days at 15 °C, the eggs were transferred onto new OP50-seeded NGM plates and kept at 25 °C or 15 °C. When the hatched larvae reached L3–L4 stages, larva/egg ratios were calculated for embryonic lethality. For 25 °C and 16 °C reproduction experiments, worms were first grown at 20 °C to a gravid adult stage. Ten worms were then transferred to 25 °C and 16 °C, respectively, and allowed to lay eggs for 24 hrs before being removed. The eggs remained at their respective temperatures for another 24 hrs to allow them to hatch. The numbers of live progeny and dead eggs were then counted after another 24 hrs. For brood size measurements, worms that developed were synchronized at L4 stage from egg stages at indicated temperatures, and the worms were transferred to new plates until they stopped laying eggs. Progeny that hatched were counted and included for the analysis.

### RNA-seq analysis

HG-cultured wild-type and *mdt-15(tm2182)* worms were synchronized by using a bleaching method [58] and incubated in M9 buffer overnight at 20 °C. The L1 worms were transferred onto OP50-seeded NGM plates and incubated at 25 °C or 15 °C until reaching day 1 adult stage. The adult worms were harvested by washing twice with M9 buffer, and then frozen at *-*80 °C. Total RNA from the worms was extracted by using RNAiso plus (Takara Bio, Shiga, Japan). Three independent biological repeats were used for subsequent analysis. The cDNA library was prepared, and cDNA sequencing was performed by using a HiSeq 4000 platform (MACROGEN, Seoul, South Korea). Paired-end reads were aligned to the *C*. *elegans* genome ce11 and analyzed by using HISAT2 (v.2.0.5), StringTie (v.1.3.3), and Ballgown (v.2.0.0) methods [60]. R packages limma (v.3.24.15) [61] and edgeR (v.3.10.5) [62] were used for analyzing differentially expressed genes (DEGs), and genes whose expression was not detected (counts per million [cpm] < 1) were excluded. Genes with significant changes in expression (fold change > 1.5 and *p*-values < 0.05) were further analyzed. Heat maps were generated by using Cluster 3.0 [63] and Java Treeview [64]. Venn diagrams were generated by using Venn Diagram Plotter (http://omics.pnl.gov/software/venn-diagram-plotter). GO analysis was performed by using DAVID [65]. Changes in the expression of metabolic genes were displayed as previously shown [66, 67], with minor modifications. Genes that were excluded during the DEG analysis are not presented in the metabolic pathways.

### Quantitative RT-PCR

Quantitative RT-PCR was performed as described previously with modifications [68]. Synchronized worms were cultured at different temperatures and were harvested at a young (day 1) adult stage by washing twice with M9 buffer. *paqr-2(tm3410)* and control (wild-type) worms were cultured at 20 °C until reaching L4 stage and then shifted to 25 °C or 15 °C for consistency with life-span assays. Total RNA was isolated using RNAiso plus (Takara Bio), and reverse transcription was performed using ImProm-II Reverse Transcriptase kit (Promega, Madison, WI, USA). Random primers (9-mers; COSMOGENTECH, Seoul, South Korea) were used for reverse transcription. Quantitative real-time PCR was performed by using StepOne and StepOnePlus real-time PCR system (Applied Biosystems, Foster City, CA, USA). Relative quantity of specific mRNA was analyzed by using comparative Ct methods described in the manufacturer’s manual. The mRNA levels of an RNA polymerase II large subunit (*ama-1*), tubulin α 1a (*tba-1*), and ATP-binding–cassette subfamily D member 4 (*pmp-3*) were used for normalization. The average of two technical repeats was used for each biological data set. Primers used for qRT-PCR assays were described in S8 Table.

### Oil red O staining

Oil Red O staining was performed as described previously with some modifications [69]. Synchronized worms were cultured on OP50-seeded NGM plates at 25 °C or 15 °C. Approximately 300 young (day 1) adult worms were harvested by washing twice with M9 buffer and fixed with 60% isopropanol for two min. Oil red O solution (0.5% in isopropanol; Sigma-Aldrich) was diluted in double-distilled water (ddH_2_O) to prepare the 60% working solution. Precipitates of Oil red O were eliminated by filtering. The fixed worms were incubated in the 60% working solution overnight at 25 °C. The stained worms were washed with M9 buffer and subsequently placed on a 2% agarose pad with M9 buffer containing 0.01% Triton X-100 (DAEJUNG) using a micropipette. DIC images were captured using an AxioCam HRc camera attached to a Zeiss Axioscope A.1 microscope (Zeiss). ImageJ [59] was used for the quantification of Oil red O intensity. The backgrounds of the images were subtracted, and the images were then converted to 8-bit grayscale images. Identical thresholds were set for the same experimental sets that measured the Oil red O signals. Areas that displayed higher intensities than the threshold in the whole body were measured, and the areas were normalized using the body size of each worm.

### Fluorescence imaging

Transgenic worms that expressed GFP were anesthetized by using 100 mM sodium azide or 2 mM levamisole (Sigma-Aldrich) and then placed on a 2% agarose pad. Images of the worms were captured by using an AxioCam HRc camera attached to a Zeiss Axioscope A.1 microscope (Zeiss). ImageJ [59] was used to quantify the fluorescence intensity, and the background signals were subtracted. High resolution confocal images were captured by using the Nikon A1si/Ni-E upright confocal microscope (Nikon, Tokyo, Japan) with 60×, 1.4 NA oil-immersion objective lens in the Brain Research Core Facilities in the Korea Brain Research Institute (KBRI). For double RNAi treatments, control and gene-specific RNAi bacteria were separately cultured in liquid LB containing 50 μg/ml ampicillin (USB) at 37 °C. Double RNAi treatment was performed as described previously [70] by adjusting OD_590_ to 0.9 and by mixing the same volumes of the RNAi bacterial culture [70]. Gene-specific RNAi bacteria were mixed with control RNAi bacteria for single RNAi treatments in the same experimental sets for comparison.

### Fatty acid composition assays

Lipid extraction was performed as described previously with some modifications [71]. Wild-type and *mdt-15(tm2182)* worms were synchronized by using a bleaching method [58] and incubated in M9 solution overnight at 20 °C. Hatched L1 worms were then cultured on OP50-seeded NGM plates at 25 °C or 15 °C. Approximately 1,000 to 1,500 young (day 1) adult worms were harvested and washed three times with ddH_2_O. The harvested worms were then frozen in liquid nitrogen and stored at *-*80 °C until use. Fatty acid methyl esters (FAMEs) were prepared by adding 2 ml of sulfuric acid (2.5%)/methanol solution to the worm samples, followed by heating at 70 °C for 1 hr. The FAMEs were extracted by using 3 ml ddH_2_O and 3 ml of hexane. The hexane layers were evaporated and analyzed by using GC/MS (GCMS-QP2010; Shimadzu, Kyoto, Japan and HP-INOWAX capillary column, 30 m, 0.25 mm; Agilent, Santa Clara, CA, USA).

### Preparation of plates for OA feeding assays

OA feeding assays were performed as described previously with modifications [16]. For preparing OA-containing plates, 600 μM of OA (C18:1n-9; Sigma-Aldrich) dissolved in ethanol was mixed with NGM containing 0.1% NP-40 (Tergitol) (Sigma-Aldrich).

### Targeted RNAi screen

Worms expressing *hsp-16*.*1p*::*hsp-16*.*1*::*GFP* were cultured on 100-mm NGM plates seeded with 2× concentrated freeze-dried OP50 (LabTIE, Leiden, the Netherlands) at 20 °C. Worms were synchronized by using a bleach method [58]. Bacteria expressing double-stranded RNA targeting each of the fat metabolism genes were cultured overnight at 37 °C and treated with 4 mM IPTG to induce the RNA for 4 hrs at 37 °C with shaking. Bacteria were concentrated by 100 times, and 20 μl of the concentrated bacteria were seeded on 24-well NGM plates containing 50 μg/ml ampicillin. The bleached eggs were cultured at 15 °C until worms reached gravid adult stages. The brightness of GFP was scored semiquantitatively by two independent researchers.

### Worm paralysis assays

Worms were synchronized as indicated for specific experiments. Synchronized worms were cultured on OP50-seeded plates at different temperatures and then transferred onto plates containing 5 μM FUdR (Sigma-Aldrich) at a young (day 1) adult stage to prevent their progeny from hatching. The paralysis of worms was determined for the ones that moved only heads upon gently touching bodies with a platinum wire. Animals that crawled off the plates, ruptured, bagged, burrowed, or were dead were censored but included in the statistical analysis.

### Statistics

Statistics used for each experiment was described in the figure legends. Briefly, *p*-values were calculated by using unpaired two-sample two-tailed Student *t* test for embryonic lethality, body size, RNA-seq, qRT-PCR, fluorescence imaging, Oil red O, and GC/MS assays. Outliers of qRT-PCR data were determined by using QuickCalcs of GraphPad (https://www.graphpad.com/quickcalcs/grubbs2) and excluded from the statistical analysis and plotting. Box plots were illustrated with BoxPlotR (http://shiny.chemgrid.org/boxplotr) by using the Tukey method [72]. For survival assays, statistical analysis was performed by using OASIS 2 (online application of survival analysis, http://sbi.postech.ac.kr/oasis2) [73] and *p*-values were calculated by using log-rank (Mantel-Cox method) test. Significance of the overlaps among gene sets was calculated by using hypergeometric probability test (http://systems.crump.ucla.edu/hypergeometric/index.php). The Cox proportional hazard regression was calculated by using OASIS (https://sbi.postech.ac.kr/oasis/surv/) using the coxph function in R coxrobust.

## Supporting information

S1 Fig MDT-15 is a limiting factor for longevity, growth and reproduction at low temperatures. (**A**) Results showing the Cox-proportional hazard ratio analysis. The hazard ratio of *mdt-15(−)* mutation was increased at 15 °C compared to that at 25 °C. See S6 Table for statistical analysis and values of the Cox-proportional hazard ratio analysis. (**B**) Lifespan curves of wild-type and *mdt-15(tm2182)* [*mdt-15(−)*] mutant animals that were shifted from 20 °C to indicated temperatures at L4 stage. (**C**) Depletion of MDT-15::degron::EmGFP by treatment with auxin at 25 °C and 15 °C. Scale bar: 50 μm. (**D**) Treatments with auxin from hatching caused developmental arrest in *mdt-15*::*degron*::*EmGFP* worms at 25 °C and 15 °C. Scale bar: 500 μm. (**E**) Treatments with auxin did not shorten the lifespan of control (*eft-3p*::*TIR1*::*mRuby*) worms at 25 °C or 15 °C. Control is a solvent (ethanol)-treated condition. (**F**) Relative body areas of wild-type and *mdt-15(−)* mutants at 25 °C and 15 °C. The relative ratios calculated from this data set are shown in Fig 1F. The size difference between wild-type and *mdt-15(−)* mutant adults was larger at 15 °C than at 25 °C. As wild-type worms displayed a comparable body size at 25 °C and 15 °C when the worms reached fully fertile adult stages, our data suggest that inhibition of *mdt-15* has a bigger impact on growth at low temperatures than at high temperatures (n = 24 for all conditions from three independent experiments, two-tailed Student *t*-tests, ***p<0.001). Box and whiskers were plotted by using the Tukey method in this and other figures. (**G**) Percentage of hatched progeny of *mdt-15(−)* mutants compared to wild-type animals at 25 °C and 16 °C (n≥589 from three independent experiments, two-tailed Student *t*-tests, **p<0.01). (**H**) Brood size of wild-type and *mdt-15(−)* animals at 25 °C and 15 °C. Each dot represents the average of each set (n≥4, two-tailed Student *t*-tests, **p<0.01, ***p<0.001). See S1 and S2 Tables for statistical analysis and additional repeats of the lifespan assays. MDT-15, Mediator 15.
(EPS)

S2 Fig Genes that are differentially regulated by MDT-15 at different temperatures. (**A**−**C**) Volcano plots represent genes up- and down-regulated in wild-type at 15 °C (**A**), and genes up-regulated at 15 °C (**B**) and down-regulated at 15 °C (**C**) in an MDT-15 dependent manner. (**D**, **E**) Gene ontology (GO) terms enriched among the genes up-regulated at 15 °C (**D**) and down-regulated at 15 °C (**E**) in an MDT-15-dependent manner. See S1 Data and GEO database (accession number(s) GSE128815) for RNA seq. data. MDT-15, Mediator 15.
(EPS)

S3 Fig MDT-15 regulates the expression of many temperature-responsive genes. (**A**, **B**) Genes up-regulated at 15 °C (**A**) and down-regulated at 15 °C (**B**) significantly overlapped with the RNA seq. data from Ma et al., 2015 [11]. Representation factor (RF) indicates relative overlaps compared to expected overlap. *p* values were calculated by using hypergeometric probability test. (**C**, **D**) Heat maps display the clusters of genes that were up-regulated (**C**) and down-regulated (**D**) at 15 °C, whose expression was reversed by *mdt-15(tm2182)* [*mdt-15(−)*] mutations compared to wild-type (WT). (**E**, **F**) Venn diagrams represent large fractions of genes whose expression was increased (**E**) or decreased at 15 °C (**F**) in an MDT-15-dependent manner. (**G**) mRNA levels of seven fatty acid desaturases in wild-type and *mdt-15(−)* at 25 °C and 15 °C obtained from RNA seq data were shown (two-tailed Student *t*-tests, *p<0.05, **p<0.01, n.s.: not significant). See S3 Table for fold change values and statistical analysis for RNA seq data as well as S1 Data and GEO database (accession number(s) GSE128815) for RNA seq. data. (**H**) Fluorescence images of *fat-7*::*GFP* in wild-type and *mdt-15(tm2182)* [*mdt-15(−)*] backgrounds at 25 °C and 15 °C. The images were obtained by using day 1 adult worms. Scale bar: 200 μm. MDT-15, Mediator 15.
(EPS)

S4 Fig Mutations in *nhr-49* decrease the expression of *fat-7*, while increasing overall fat levels at low temperatures. (**A**) mRNA levels of *fat-7* in wild-type and *nhr-49(gk405)* [*nhr-49(−)*] worms at 15 °C were measured by using qRT-PCR (n = 4, two-tailed Student *t*-tests, ***p<0.001). (**B**) Overall fat levels of wild-type and *nhr-49(−)* animals at 25 °C and 15 °C were measured by using Oil red O staining. Scale bar: 200 μm. (**C**) Quantification of the Oil red O signals in panel B (n≥29 from three independent experiments, two-tailed Student *t*-tests, ***p<0.001). (**D**) Lifespan curves of wild-type and *skn-1(zj15)* [*skn-1(−)*] animals at 25 °C and 15 °C. (**E**) Effects of *sbp-1* RNAi on the lifespan of wild-type at 25 °C and 15 °C. Note that low temperatures may have affected RNAi efficiency that is dependent on bacterial metabolism [75]. See S1 Table for statistical analysis and additional repeats of the lifespan assays.
(EPS)

S5 Fig The effects of *mdt-15(−)* mutations on the expression of mitochondrial and ER chaperones, and proteasome and autophagy components. (**A**) qRT-PCR data represent mRNA levels of ER and mitochondrial chaperone genes in wild-type and *mdt-15(−)* worms at 15 °C. *hsp-6* (n = 6), *hsp-60* (n = 6) and *Y22D7AL*.*10* (n = 3) are mitochondrial chaperone genes [76], whereas *hsp-4* (n = 5), *enpl-1* (n = 3), and *hsp-3* (n = 3) encode ER chaperones [77, 78]. *ama-1* was used as a normalization control for *hsp-6*, *hsp-60* and *hsp-4* qRT-PCR, and *pmp-3* was used for *Y22D7AL*.*10*, *enpl-1* and *hsp-3*. (**B**, **C**) qRT-PCR data represent mRNA levels of genes encoding various components of proteasome (**B**) and autophagy (**C**) systems (n = 4). We noticed that the expression of several proteasome-related genes, including *pas-4*, *pas-5*, *outb-1* and *csn-5*, tended to be increased at low temperatures, but the effect was independent of *mdt-15(−)*. Both *pmp-3* and *tba-1* were used as normalization controls for the qRT-PCR analysis of proteasome and autophagy genes. (**D**) Relative mRNA levels of *fat-6* and *fat-7* measured by using qRT-PCR upon treating with two different RNAi clones that target *fat-6* and *fat-7* (*fat-6/7* RNAi and *fat-6/7* RNAi*). As *fat-6/7* RNAi* displayed highly variable effects on the expression of *fat-6*, we excluded the subsequent experimental data using the *fat-6/7* RNAi*, and showed the data using *fat-6/7* RNAi in Fig 5G, 5H and 5J. *mdt-15* RNAi was used as a positive control, and *pmp-3* was used as a normalization control for qRT-PCR (n≥4, two-tailed Student *t*-tests, *p<0.05, **p<0.01, ***p<0.001). ER, endoplasmic reticulum; MDT-15, Mediator 15.
(EPS)

S6 Fig Oleic acid supplementation decreased the elevated *hsp-16*.*1*::*GFP* levels and rescued the small body size of *mdt-15*-inhibited worms at low temperatures. (**A**) Images of *hsp-16*.*1*::*GFP* transgenic worms treated with control RNAi or *mdt-15* RNAi at 15 °C on control or oleic acid (OA)-containing diets. (**B**) Quantification of the *hsp-16*.*1*::*GFP* using the conditions same as the panel A (n≥23 from three independent experiments). Images of individual worms were obtained separately for the quantification. Scale bar: 200 μm. (**C**) OA feeding increased the relatively small body size of *mdt-15(−)* animals at 15 °C (n≥24 from three independent experiments, two-tailed Student *t*-tests, ***p<0.001). GFP, green fluorescent protein; *hsp*, heat shock protein; MDT-15, Mediator 15; OA, oleic acid.
(EPS)

S1 Table Statistical analysis and additional repeats of life-span assays with mutants, RNAi, or dietary glucose. RNAi, RNA interference.
(DOCX)

S2 Table Statistical analysis and additional repeats of life-span assays with or without auxin treatment. (DOCX)

S3 Table Expression changes of genes involved in metabolism from RNA-seq data. RNA-seq, RNA sequencing.
(DOCX)

S4 Table Statistical analysis and additional repeats of life-span assays with or without OA supplementation. OA, oleic acid.
(DOCX)

S5 Table Statistical analysis and additional repeats of paralysis assays. (DOCX)

S6 Table The Cox proportional hazard regression analysis. (DOCX)

S7 Table Standard deviations for box plot data. (DOCX)

S8 Table Primers used for quantitative RT-PCR assays. (DOCX)

S1 Data The raw data shown by box plots in Figures and Supplementary figures. (XLSX)

S2 Data List of genes that were up- or down-regulated at 15 °C in an MDT-15–dependent manner. MDT-15, Mediator 15.
(XLSX)

## Abbreviations

acaa: acetyl-CoA acyltransferase
ACC: acetyl-CoA carboxylase
ACDH: acyl-CoA dehydrogenase
ACO: acyl-CoA oxidase
ACS: acyl-CoA synthetase
aldo: aldolase
atgl: adipose triglyceride lipase
cpm: counts per million
CPT: carnitine palmitoyl transferase
CS: citrate synthase
cts: citrate synthase
cyc: cyclo
DAF: dauer formation
ddH_2_O: double-distilled water
DEG: differentially expressed gene
dld: dihydrolipoamide dehydrogenase
ECH: enoyl-CoA hydratase
eft-3p: eukaryotic translation elongation factor 3 promoter
ELO: fatty acid elongase
EmGFP: Emerald green fluorescent protein
enol: enolase
enpl-1: endoplasmin 1
ER: endoplasmic reticulum
FAME: fatty acid methyl ester
FAR: fatty-acid–and retinol-binding protein
FAS: fatty acid synthase
FOXO: Forkhead box O transcription factor
FPKM: fragments per kilobase of exon model per million reads mapped
FUdR: 5-fluoro-2′-deoxyuridine
fum: fumarase
GAPDH: glyceraldehyde 3-phosphate dehydrogenase
GC/MS: gas chromatography/mass spectrometry
GEO: Gene Expression Omnibus
GFP: green fluorescent protein
GO: gene ontology
gof: gain of function
gpd: glyceraldehyde 3-phosphate dehydrogenase
GPI: glucose-6-phosphate isomerase
HACD: hydroxyacyl-CoA dehydrogenase
HG: high growth
hosl: hormone-sensitive lipase
HSF-1: heat shock factor 1
hsp: heat shock protein
hxk: hexokinase
ipgm: cofactor-independent phosphoglycerate mutase
IPTG: isopropyl β-D-1-thiogalactopyranoside
iso: isomer
kat: 3-ketoacyl-coA thiolase
KBRI: Korea Brain Research Institute
LBP: lipid-binding protein
let: lethal
lipl: lipase-like
MDH: malate dehydrogenase
MDT-15: Mediator 15
MED15: mammalian Mediator 15
NGM: nematode growth medium
nhr: nuclear hormone receptor
NPC1: Niemann-Pick C1
n.s.: not significant
OA: oleic acid
OASIS: online application of survival analysis
paqr: progestin and adipoQ receptor
PFK: phospho-fructo-kinase
PGK: phosphoglycerate kinase
PGM: phosphoglycerate mutase
PK: pyruvate kinase
pod: polarity and osmotic sensitivity defect
polyQ: polyglutamine
pyk: pyruvate kinase
qRT-PCR: quantitative reverse-transcription PCR
RNAi: RNA interference
RNA-seq: RNA sequencing
sbp: sterol regulatory element binding protein
SCS: succinyl-CoA synthetase
SDH: succinate dehydrogenase
SFA: saturated fatty acid
skn: skinhead
SREBP: sterol regulatory element binding protein
suc: succinyl-CoA ligase
tba-1: tubulin α 1a
TIR1: transport inhibitor response 1
TPI: triosephosphate isomerase
UFA: unsaturated fatty acid
UPR^ER^: ER unfolded protein response
YFP: yellow fluorescent protein
α-KGDH: α-ketoglutarate dehydrogenase
