# Supplementary material for: MDT-15/MED15 permits longevity at low temperature via enhancing lipidostasis and proteostasis
Source: PLoS Biol. 2019 Aug 13;17(8):e3000415. doi: 10.1371/journal.pbio.3000415 (PMC6692015; doi:10.1371/journal.pbio.3000415)
Supplement: S1 Table — RNAi, RNA interference. (DOCX) [file pbio.3000415.s007.docx]

**S1 Table.** Statistical analysis and additional repeats of lifespan assays with mutants, RNAi or dietary glucose.

| Strain/treatment | Mean lifespan ±s.e.m. (days) | 75th percentile | % change^Δ^ | Number of animals that died/total | *p* value vs. control | Figure in text |
| --- | --- | --- | --- | --- | --- | --- |
| Wild-type/25°C | 10.9±0.4 | 17 |  | 83/125 |  | Fig 1A |
| Wild-type/15°C | 23.4±0.7 | 34 | +116% | 75/125 | <0.0001 | Fig 1A |
| *mdt-15(tm2182)*/25°C | 9.6±0.3 | 15 | -12% | 99/125 | 0.0089 | Fig 1A |
| *mdt-15(tm2182)*/15°C | 13.7±0.4 | 19 | +43%  -41%^(WT 15°C)^ | 96/125 | <0.0001  <0.0001^(WT 15°C)^ | Fig 1A |
| Wild-type/25°C | 9.9±0.3 | 14 |  | 90/120 |  |  |
| Wild-type/15°C | 21.0±0.9 | 31 | +111% | 62/120 | <0.0001 |  |
| *mdt-15(tm2182)*/25°C | 9.8±0.4 | 16 | -2% | 85/120 | 0.9047 |  |
| *mdt-15(tm2182)*/15°C | 13.1±0.4 | 18 | +34%  -38%^(WT 15°C)^ | 88/120 | <0.0001  <0.0001^(WT 15°C)^ |  |
| Wild-type/25°C | 13.4±0.4 | 19 |  | 86/125 |  |  |
| Wild-type/15°C | 23.8±1.1 | 37 | +78% | 60/125 | <0.0001 |  |
| *mdt-15(tm2182)*/25°C | 11.0±0.4 | 16 | -18% | 109/125 | 0.0003 |  |
| *mdt-15(tm2182)*/15°C | 16.2±0.6 | 23 | +47%  -32%^(WT 15°C)^ | 108/125 | <0.0001  <0.0001^(WT 15°C)^ |  |
| Wild-type/25°C | 15.3±0.4 | 20 |  | 118/125 |  | Fig 1E |
| Wild-type/15°C | 26.0±0.7 | 37 | +70% | 120/125 | <0.0001 | Fig 1E |
| *mdt-15(yh8)*/25°C | 16.2±0.3 | 20 | +6% | 138/150 | 0.0429 | Fig 1E |
| *mdt-15(yh8)*/15°C | 25.7±0.5 | 34 | +58%  -1%^(WT 15°C)^ | 145/150 | <0.0001  0.3352^(WT 15°C)^ | Fig 1E |
| *skn-1(zj15)*/25°C | 16.1±0.3 | 20 | +5% | 116/125 | 0.1575 |  |
| *skn-1(zj15)*/15°C | 24.6±0.8 | 34 | +52%  -6%^(WT 15°C)^ | 108/125 | <0.0001  0.4810^(WT 15°C)^ |  |
| Wild-type/25°C | 11.4±0.2 | 14 |  | 116/125 |  | S4D Fig |
| Wild-type/15°C | 24.1±0.6 | 33 | +112% | 105/125 | <0.0001 | S4D Fig |
| *mdt-15(yh8)*/25°C | 10.8±0.2 | 14 | -5% | 129/150 | <0.0001 |  |
| *mdt-15(yh8)*/15°C | 25.8±0.5 | 33 | +138%  +7%^(WT 15°C)^ | 114/150 | <0.0001  0.1664^(WT 15°C)^ |  |
| *skn-1(zj15)*/25°C | 11.4±0.2 | 14 | +1% | 117/125 | <0.0001 | S4D Fig |
| *skn-1(zj15)*/15°C | 21.2±0.5 | 29 | +85%  -12%^(WT 15°C)^ | 94/125 | <0.0001  <0.0001^(WT 15°C)^ | S4D Fig |
| Wild-type/25°C | 11.9±0.4 | 17 |  | 86/125 |  |  |
| Wild-type/15°C | 28.1±0.7 | 36 | +136% | 112/125 | <0.0001 |  |
| *mdt-15(yh8)*/25°C | 11.0±0.5 | 17 | -8% | 57/125 | 0.2187 |  |
| *mdt-15(yh8)*/15°C | 27.8±1.0 | 39 | +153%  -1%^(WT 15°C)^ | 63/125 | <0.0001  0.4520^(WT 15°C)^ |  |
| *nhr-49(gk405)*/25°C | 10.9±0.2 | 14 | -9% | 123/125 | 0.0002 |  |
| *nhr-49(gk405)*/15°C | 18.8±0.5 | 27 | +72%  -33%^(WT 15°C)^ | 119/125 | <0.0001  <0.0001^(WT 15°C)^ |  |
| Wild-type/25°C | 9.9±0.2 | 12 |  | 88/125 |  | Fig 4C |
| Wild-type/15°C | 25.2±0.4 | 32 | +154% | 117/125 | <0.0001 | Fig 4C |
| *mdt-15(yh8)*/25°C | 10.3±0.4 | 14 | +4% | 52/125 | 0.3271 |  |
| *mdt-15(yh8)*/15°C | 28.6±0.8 | 38 | +178%  +14%^(WT 15°C)^ | 65/125 | <0.0001  <0.0001^(WT 15°C)^ |  |
| *nhr-49(gk405)*/25°C | 8.8±0.3 | 13 | -12% | 120/125 | 0.0220 | Fig 4C |
| *nhr-49(gk405)*/15°C | 16.3±0.4 | 20 | +86%  -35%^(WT 15°C)^ | 103/125 | <0.0001  <0.0001^(WT 15°C)^ | Fig 4C |
| Wild-type/25°C* | 10.9±0.2 | 14 |  | 114/125 |  | Fig 4A; S1B Fig |
| Wild-type/15°C* | 29.2±0.7 | 38 | +167% | 101/125 | <0.0001 | Fig 4A; S1B Fig |
| *fat-6(tm331); fat-7(wa36)*/25°C* | 11.3±0.4 | 16 | +4% | 68/75 | 0.0830 | Fig 4A |
| *fat-6(tm331); fat-7(wa36)*/15°C* | 20.8±0.6 | 26 | +83%  -29%^(WT 15°C)^ | 80/90 | <0.0001  <0.0001^(WT 15°C)^ | Fig 4A |
| *mdt-15(tm2182)*/25°C* | 10.0±0 | 14 | -8% | 114/125 | 0.0345 | S1B Fig |
| *mdt-15(tm2182)*/15°C* | 19.5±0.4 | 26 | +94%  -33%^(WT 15°C)^ | 120/125 | <0.0001  <0.0001^(WT 15°C)^ | S1B Fig |
| Wild-type/25°C* | 13.7±0.3 | 16 |  | 77/101 |  |  |
| Wild-type/15°C* | 28.5±0.6 | 37 | +108% | 82/100 | <0.0001 |  |
| *fat-6(tm331); fat-7(wa36)*/25°C* | 12.6±0.4 | 16 | -8% | 79/82 | 0.2753 |  |
| *fat-6(tm331); fat-7(wa36)*/15°C* | 21.0±0.7 | 28 | +67%  -26%^(WT 15°C)^ | 83/91 | <0.0001  <0.0001^(WT 15°C)^ |  |
| *mdt-15(tm2182)*/25°C* | 13.0±0.4 | 18 | -5% | 96/100 | 0.7854 |  |
| *mdt-15(tm2182)*/15°C* | 20.9±0.6 | 28 | +61%  -26%^(WT 15°C)^ | 96/100 | <0.0001  <0.0001^(WT 15°C)^ |  |
| Wild-type/15°C | 25.3±0.7 | 34 |  | 78/125 |  | Fig 4D |
| *mdt-15(tm2182)*/15°C | 17.3±0.4 | 22 | -32% | 116/125 | <0.0001 | Fig 4D |
| *nhr-49(gk405)*/15°C | 16.5±0.3 | 22 | -35% | 111/125 | <0.0001 | Fig 4D |
| *nhr-49(gk405); mdt-15(tm2182)*/15°C | 16.6±0.3 | 22 | -4%^(^*^mdt-15^* ^15°C)^  +1%^(^*^nhr-49^* ^15°C)^ | 119/125 | 0.0928^(^*^mdt-15^* ^15°C)^  0.8565^(^*^nhr-49^* ^15°C)^ | Fig 4D |
| Wild-type/15°C | 25.6±0.7 | 37 |  | 106/125 |  |  |
| *mdt-15(tm2182)*/15°C | 20.7±0.5 | 25 | -19% | 116/125 | <0.0001 |  |
| *nhr-49(gk405)*/15°C | 20.3±0.6 | 29 | -21% | 112/125 | <0.0001 |  |
| *nhr-49(gk405); mdt-15(tm2182)*/15°C | 20.9±0.5 | 29 | +1%^(^*^mdt-15^* ^15°C)^  +3%^(^*^nhr-49^* ^15°C)^ | 101/125 | 0.7421^(^*^mdt-15^* ^15°C)^  0.3939^(^*^nhr-49^* ^15°C)^ |  |
| Wild-type/25°C* | 8.7±0.2 | 10 |  | 112/120 |  | Fig 4B |
| Wild-type/15°C* | 27.7±0.7 | 32 | +218% | 84/120 | <0.0001 | Fig 4B |
| *paqr-2(tm3410)*/25°C* | 9.1±0.2 | 10 | +4% | 106/120 | 0.1876 | Fig 4B |
| *paqr-2(tm3410)*/15°C* | 12.7±0.4 | 14 | +39%  -54%^(WT 15°C)^ | 81/120 | <0.0001  <0.0001^(WT 15°C)^ | Fig 4B |
| Wild-type/25°C* | 8.6±0.3 | 10 |  | 92/120 |  |  |
| Wild-type/15°C* | 27.5±0.8 | 32 | +218% | 87/120 | <0.0001 |  |
| *paqr-2(tm3410)*/25°C* | 8.7±0.2 | 10 | +0% | 110/120 | 0.7758 |  |
| *paqr-2(tm3410)*/15°C* | 15.7±0.2 | 16 | +81%  -43%^(WT 15°C)^ | 80/120 | <0.0001  <0.0001^(WT 15°C)^ |  |
| Wild-type/25°C | 10.6±0.3 | 12 |  | 100/120 |  | Fig 4E |
| Wild-type/15°C | 26.5±0.7 | 30 | +151% | 81/120 | <0.0001 | Fig 4E |
| Wild-type/25°C glucose | 10.8±0.2 | 14 | +2% | 87/120 | 0.8465 | Fig 4E |
| Wild-type/15°C glucose | 15.3±0.4 | 18 | +42%  -42%^(Ctrl 15°C)^ | 72/120 | <0.0001  <0.0001^(Ctrl 15°C)^ | Fig 4E |
| Wild-type/25°C | 14.3±0.2 | 16 |  | 115/120 |  |  |
| Wild-type/15°C | 31.6±1.0 | 41 | +120% | 78/120 | <0.0001 |  |
| Wild-type/25°C glucose | 13.0±0.3 | 16 | -9% | 99/120 | <0.0001 |  |
| Wild-type/15°C glucose | 16.0±0.4 | 20 | +23%  -49%^(Ctrl 15°C)^ | 83/120 | <0.0001  <0.0001^(Ctrl 15°C)^ |  |
| Wild-type/25°C Kan* | 10.2±0.2 | 14 |  | 99/120 |  | Fig 4F |
| Wild-type/15°C Kan* | 40.8±0.9 | 45 | +300% | 92/120 | <0.0001 | Fig 4F |
| Wild-type/25°C glucose Kan* | 9.4±0.1 | 12 | -8% | 104/120 | <0.0001 | Fig 4F |
| Wild-type/15°C glucose Kan* | 15.1±0.5 | 18 | +61%  -63%^(Ctrl 15°C)^ | 84/120 | <0.0001  <0.0001^(Ctrl 15°C)^ | Fig 4F |
| Wild-type/25°C Kan* | 10.2±0.3 | 15 |  | 115/120 |  |  |
| Wild-type/15°C Kan* | 34.5±1.0 | 43 | +237% | 99/120 | <0.0001 |  |
| Wild-type/25°C glucose Kan* | 8.9±0.1 | 12 | -13% | 115/120 | <0.0001 |  |
| Wild-type/15°C glucose Kan* | 13.7±0.4 | 19 | +54%  -60%^(Ctrl 15°C)^ | 105/120 | <0.0001  <0.0001^(Ctrl 15°C)^ |  |
| Wild-type/25°C control RNAi* | 15.3±0.3 | 18 |  | 85/120 |  | S4E Fig |
| Wild-type/15°C control RNAi* | 24.1±0.4 | 26 | +58% | 84/120 | <0.0001 | S4E Fig |
| Wild-type/25°C *sbp-1* RNAi* | 13.3±0.2 | 16 | -13% | 110/120 | <0.0001 | S4E Fig |
| Wild-type/15°C *sbp-1* RNAi* | 22.7±0.5 | 24 | +70%  -6%^(Ctrl 15°C)^ | 105/120 | <0.0001  0.0223^(Ctrl 15°C)^ | S4E Fig |
| Wild-type/25°C control RNAi* | 16.9±0.3 | 21 |  | 115/120 |  |  |
| Wild-type/15°C control RNAi* | 23.5±0.4 | 27 | +39% | 115/120 | <0.0001 |  |
| Wild-type/25°C *sbp-1* RNAi* | 11.6±0.4 | 14 | -31% | 118/121 | <0.0001 |  |
| Wild-type/15°C *sbp-1* RNAi* | 21.7±0.4 | 27 | +87%  -7%^(Ctrl 15°C)^ | 98/120 | <0.0001  0.0659^(Ctrl 15°C)^ |  |

Lifespan data within the double-solid lines are same experimental sets and biological replicates are separated by solid lines. Lifespan data within the solid lines were performed at the same time. All *p* values were calculated within the individual sets by using the log-rank (Mantel-Cox) method.

Percent (%) changes and *p* values for 15°C conditions were calculated against 25°C conditions within dashed lines in the same experimental set.

Percent (%) changes and *p* values for 25°C conditions were calculated against wild-type/25°C or wild-type/25°C control RNAi within dashed lines in the same experimental set.

^WT 15°C^: percent (%) changes and *p* values calculated against wild-type 15°C in the same experimental sets

^Ctrl 15°C^: percent (%) changes and *p* values calculated against wild-type/15°C control RNAi control treatment in the same experimental sets

* indicates temperature shift experiments from 20°C to indicated temperatures at L4 stage.
