# Supplementary material for: MDT-15/MED15 permits longevity at low temperature via enhancing lipidostasis and proteostasis
Source: PLoS Biol. 2019 Aug 13;17(8):e3000415. doi: 10.1371/journal.pbio.3000415 (PMC6692015; doi:10.1371/journal.pbio.3000415)
Supplement: S2 Table — (DOCX) [file pbio.3000415.s008.docx]

**S2 Table.** Statistical analysis and additional repeats of lifespan assays with or without auxin treatment

| Strain/treatment | Mean lifespan ±s.e.m. (days) | 75th percentile | % change^Δ^ | Number of animals that died/total | *p* value vs. control | Figure in text |
| --- | --- | --- | --- | --- | --- | --- |
| *eft-3p::TIR1::mRuby*; *mdt-15::degron::EmGFP*/25°C control | 9.5±0.2 | 12 |  | 106/120 |  | Fig 1D |
| *eft-3p::TIR1::mRuby*; *mdt-15::degron::EmGFP* /15°C control | 19.4±0.3 | 22 | +105% | 112/120 | <0.0001 | Fig 1D |
| *eft-3p::TIR1::mRuby*; *mdt-15::degron::EmGFP* /25°C auxin | 8.6±0.2 | 10 | -9% | 117/120 | 0.0003 | Fig 1D |
| *eft-3p::TIR1::mRuby*; *mdt-15::degron::EmGFP* /15°C auxin | 13.9±0.2 | 14 | +62%  -28%^(Ctrl 15°C)^ | 117/120 | <0.0001  <0.0001^(Ctrl 15°C)^ | Fig 1D |
| *eft-3p::TIR1::mRuby* /25°C control | 11.1±0.2 | 14 |  | 110/120 |  | S1E Fig |
| *eft-3p::TIR1::mRuby* /15°C control | 25.6±0.3 | 30 | +131% | 92/120 | <0.0001 | S1E Fig |
| *eft-3p::TIR1::mRuby* /25°C auxin | 12.4±0.2 | 16 | +12% | 115/120 | <0.0001 | S1E Fig |
| *eft-3p::TIR1::mRuby* /15°C auxin | 29.4±0.4 | 34 | +138%  +15%^(Ctrl 15°C)^ | 86/120 | <0.0001  <0.0001^(Ctrl 15°C)^ | S1E Fig |
| *eft-3p::TIR1::mRuby*; *mdt-15::degron::EmGFP /*25°C control | 12.5±0.3 | 16 |  | 106/120 |  |  |
| *eft-3p::TIR1::mRuby*; *mdt-15::degron::EmGFP /*15°C control | 23.4±0.5 | 32 | +87% | 115/120 | <0.0001 |  |
| *eft-3p::TIR1::mRuby*; *mdt-15::degron::EmGFP /*25°C auxin | 11.3±0.3 | 16 | -9% | 115/120 | 0.0117 |  |
| *eft-3p::TIR1::mRuby*; *mdt-15::degron::EmGFP /*15°C auxin | 19.0±0.4 | 26 | +67%  -19%^(Ctrl 15°C)^ | 113/120 | <0.0001  <0.0001^(Ctrl 15°C)^ |  |
| *eft-3p::TIR1::mRuby /*25°C control | 14.0±0.3 | 19 |  | 108/120 |  |  |
| *eft-3p::TIR1::mRuby /*15°C control | 31.0±0.7 | 38 | +122% | 110/120 | <0.0001 |  |
| *eft-3p::TIR1::mRuby /*25°C auxin | 15.4±0.3 | 19 | +10% | 115/120 | 0.0012 |  |
| *eft-3p::TIR1::mRuby /*15°C auxin | 35.7±0.7 | 42 | +132%  +15%^(Ctrl 15°C)^ | 96/120 | <0.0001  <0.0001^(Ctrl 15°C)^ |  |
| *eft-3p::TIR1::mRuby*; *mdt-15::degron::EmGFP /*25°C control | 8.5±0.2 | 10 |  | 108/120 |  |  |
| *eft-3p::TIR1::mRuby*; *mdt-15::degron::EmGFP /*15°C control | 17.0±0.3 | 18 | +101% | 116/120 | <0.0001 |  |
| *eft-3p::TIR1::mRuby*; *mdt-15::degron::EmGFP /*25°C auxin | 7.3±0.2 | 10 | -14% | 118/121 | <0.0001 |  |
| *eft-3p::TIR1::mRuby*; *mdt-15::degron::EmGFP /*15°C auxin | 13.8±0.2 | 18 | +90%  -19%^(Ctrl 15°C)^ | 113/120 | <0.0001  <0.0001^(Ctrl 15°C)^ |  |
| *eft-3p::TIR1::mRuby /*25°C control | 9.8±0.2 | 12 |  | 109/120 |  |  |
| *eft-3p::TIR1::mRuby /*15°C control | 22.5±0.4 | 29 | +130% | 100/120 | <0.0001 |  |
| *eft-3p::TIR1::mRuby /*25°C auxin | 10.9±0.2 | 14 | +11% | 113/120 | 0.0003 |  |
| *eft-3p::TIR1::mRuby /15°C auxin* | 29.0±0.5 | 33 | +167%  +29%^(Ctrl 15°C)^ | 81/120 | <0.0001  <0.0001^(Ctrl 15°C)^ |  |

Lifespan assays were performed with or without auxin on the media containing ethanol as the solvent (See the “Lifespan assays” section in Materials and Methods for detail information).

Lifespan data within the solid lines are biological replicates and were performed at the same time. All *p* values were calculated within the individual sets by using the log-rank (Mantel-Cox) method.

Percent (%) changes and *p* values for 15°C conditions were calculated against 25°C conditions within dashed lines in the same experimental set.

Percent (%) changes and *p* values for 25°C conditions were calculated against control/25°C within dashed lines in the same experimental set.

^Ctrl 15°C^: percent (%) changes and *p* values calculated against control/15°C in the same experimental sets
