# Supplementary material for: MDT-15/MED15 permits longevity at low temperature via enhancing lipidostasis and proteostasis
Source: PLoS Biol. 2019 Aug 13;17(8):e3000415. doi: 10.1371/journal.pbio.3000415 (PMC6692015; doi:10.1371/journal.pbio.3000415)
Supplement: S3 Table — RNA-seq, RNA sequencing. (DOCX) [file pbio.3000415.s009.docx]

**S3 Table.** Expression changes of genes involved in metabolism from RNA seq. data

| **Pathway** | **Function** | **Gene name** | **Fold Change**  **(N2 25°C vs. N2 15°C)** | ***p* value**  **(N2 25°C vs. N2 15°C)** | **Fold Change**  **(N2 15°C vs. *mdt-15(-)* 15°C)** | ***p* value**  **(N2 15°C vs. *mdt-15(-)* 15°C)** |
| --- | --- | --- | --- | --- | --- | --- |
| Glycolysis | Hexokinase | *hxk-1* | 1.30 | 0.2621 | 0.60 | 0.0421 |
|  |  | *hxk-2* | 0.69 | 0.0409 | 0.88 | 0.4231 |
|  | GPI | *gpi-1* | 1.14 | 0.6839 | 0.98 | 0.9515 |
|  | PFK | *pfk-1.1* | 0.98 | 0.9026 | 1.05 | 0.7341 |
|  |  | *pfk-1.2* | 1.02 | 0.9538 | 4.14 | 0.0013 |
|  |  | *pfkb-1.1* | 1.06 | 0.6897 | 0.74 | 0.0738 |
|  |  | *pfkb-1.2* | 1.04 | 0.7439 | 0.80 | 0.0927 |
|  | Aldolase | *aldo-1* | 1.10 | 0.7433 | 3.59 | 0.0008 |
|  |  | *aldo-2* | 0.94 | 0.8655 | 0.70 | 0.3286 |
|  | TPI | *tpi-1* | 1.00 | 0.9894 | 0.86 | 0.2194 |
|  | GAPDH | *gpd-1* | 0.91 | 0.6913 | 0.72 | 0.1798 |
|  |  | *gpd-2* | 0.87 | 0.8404 | 1.29 | 0.7140 |
|  |  | *gpd-3* | 1.08 | 0.8786 | 1.43 | 0.5036 |
|  |  | *gpd-4* | 1.08 | 0.7127 | 0.71 | 0.1073 |
|  | PGK | *pgk-1* | 0.79 | 0.1588 | 1.03 | 0.8540 |
|  | PGM | *ipgm-1* | 1.04 | 0.7390 | 1.22 | 0.0966 |
|  | Enolase | *enol-1* | 1.07 | 0.7202 | 1.13 | 0.5348 |
|  | PK | *pyk-1* | 0.98 | 0.9224 | 0.91 | 0.6914 |
|  |  | *pyk-2* | 0.74 | 0.0384 | 1.05 | 0.7159 |
| Krebs cycle | CS | *cts-1* | 1.05 | 0.8145 | 0.73 | 0.1648 |
|  | Aconitase | *aco-2* | 1.03 | 0.8835 | 0.90 | 0.5434 |
|  | IDH | *idha-1* | 1.11 | 0.6421 | 0.78 | 0.3070 |
|  |  | *idhb-1* | 1.00 | 0.9848 | 0.79 | 0.2572 |
|  |  | *idhg-1* | 1.25 | 0.2442 | 0.66 | 0.0453 |
|  |  | *idhg-2* | 0.96 | 0.7599 | 0.99 | 0.9192 |
|  |  | *idh-2* | 0.80 | 0.0827 | 0.96 | 0.7449 |
|  | α-KGDH | *ogdh-1* | 1.19 | 0.5354 | 0.81 | 0.4469 |
|  |  | *dlst-1* | 0.94 | 0.6482 | 0.85 | 0.2452 |
|  |  | *dld-1* | 1.08 | 0.5599 | 0.64 | 0.0058 |
|  | SCS | *sucg-1* | 1.31 | 0.0275 | 0.66 | 0.0024 |
|  |  | *suca-1* | 0.59 | 0.0040 | 1.68 | 0.0049 |
|  |  | *sucl-1* | 0.96 | 0.8163 | 1.02 | 0.9223 |
|  |  | *sucl-2* | 1.18 | 0.6441 | 0.49 | 0.0718 |
|  | SDH | *sdha-1* | 1.15 | 0.6808 | 0.82 | 0.5628 |
|  |  | *sdhb-1* | 1.11 | 0.4127 | 0.90 | 0.3986 |
|  |  | *sdhd-1* | 1.38 | 0.3775 | 0.73 | 0.3920 |
|  | Fumarase | *fum-1* | 1.00 | 0.9872 | 0.93 | 0.7977 |
|  | MDH | *mdh-1* | 0.95 | 0.8541 | 0.79 | 0.3738 |
| Fatty acid synthesis | ACC | *pod-2* | 0.80 | 0.3639 | 1.17 | 0.5334 |
|  | FAS | *fasn-1* | 1.19 | 0.6510 | 0.95 | 0.8995 |
|  | FAT | *fat-1* | 0.81 | 0.4021 | 0.53 | 0.0221 |
|  |  | *fat-2* | 0.63 | 0.1732 | 0.42 | 0.0187 |
|  |  | *fat-3* | 0.85 | 0.3726 | 1.10 | 0.5808 |
|  |  | *fat-4* | 1.04 | 0.8410 | 0.88 | 0.5644 |
|  |  | *fat-5* | 0.94 | 0.7689 | 0.01 | <0.0001 |
|  |  | *fat-6* | 0.85 | 0.6681 | 0.31 | 0.0107 |
|  |  | *fat-7* | 20.73 | <0.0001 | 0.00 | <0.0001 |
|  | ELO | *elo-1* | 1.62 | 0.3702 | 0.53 | 0.2451 |
|  |  | *elo-2* | 1.46 | 0.3361 | 0.75 | 0.4524 |
|  |  | *elo-3* | 1.68 | 0.3655 | 0.53 | 0.2778 |
|  |  | *elo-4* | 2.47 | 0.0806 | 1.15 | 0.7202 |
|  |  | *elo-5* | 0.68 | 0.0843 | 1.30 | 0.2295 |
|  |  | *elo-6* | 0.88 | 0.7189 | 0.49 | 0.0671 |
|  |  | *elo-8* | 0.52 | 0.2607 | 3.86 | 0.0271 |
|  |  | *elo-9* | 1.49 | 0.3383 | 0.62 | 0.2572 |
|  |  | *let-767* | 1.44 | 0.2881 | 0.77 | 0.4440 |
| Lipolysis | Lipase | *lipl-1* | 0.68 | 0.2586 | 8.20 | <0.0001 |
|  |  | *lipl-2* | 0.17 | 0.0001 | 2.04 | 0.0623 |
|  |  | *lipl-3* | 0.64 | 0.1528 | 0.26 | 0.0073 |
|  |  | *lipl-5* | 0.89 | 0.3945 | 1.12 | 0.4007 |
|  |  | *lipl-7* | 1.59 | 0.0686 | 0.95 | 0.8335 |
|  |  | *hosl-1* | 1.33 | 0.4563 | 0.52 | 0.1188 |
|  |  | *atgl-1* | 0.81 | 0.8160 | 2.62 | 0.2821 |
| Fatty acid *β*-oxidation | ACS | *acs-1* | 0.93 | 0.7687 | 2.73 | 0.0017 |
|  |  | *acs-2* | 1.84 | 0.0312 | 0.05 | <0.0001 |
|  |  | *acs-3* | 0.59 | 0.0237 | 1.26 | 0.2666 |
|  |  | *acs-4* | 1.49 | 0.2719 | 0.69 | 0.3006 |
|  |  | *acs-5* | 0.89 | 0.4633 | 0.79 | 0.1590 |
|  |  | *acs-6* | 1.31 | 0.3803 | 2.58 | 0.0049 |
|  |  | *acs-7* | 0.50 | 0.0020 | 0.42 | 0.0008 |
|  |  | *acs-10* | 0.71 | 0.4757 | 5.03 | 0.0034 |
|  |  | *acs-11* | 1.59 | 0.3288 | 0.63 | 0.3337 |
|  |  | *acs-12* | 0.79 | 0.1873 | 1.55 | 0.0226 |
|  |  | *acs-13* | 1.39 | 0.1665 | 0.59 | 0.0366 |
|  |  | *acs-14* | 1.50 | 0.0276 | 1.37 | 0.0644 |
|  |  | *acs-16* | 0.97 | 0.9144 | 2.40 | 0.0041 |
|  |  | *acs-17* | 3.47 | 0.0026 | 0.98 | 0.9325 |
|  |  | *acs-19* | 1.08 | 0.6818 | 0.60 | 0.0153 |
|  |  | *acs-20* | 1.31 | 0.3303 | 0.88 | 0.6270 |
|  |  | *acs-21* | 0.77 | 0.2534 | 1.46 | 0.1064 |
|  |  | *acs-22* | 2.81 | 0.3488 | 0.28 | 0.2482 |
|  | CPT | *cpt-1* | 0.88 | 0.6186 | 0.63 | 0.0992 |
|  |  | *cpt-2* | 1.22 | 0.1693 | 0.79 | 0.1111 |
|  |  | *cpt-5* | 1.11 | 0.4480 | 0.08 | <0.0001 |
|  |  | *cpt-6* | 0.74 | 0.1021 | 0.46 | 0.0018 |
|  | ACO | *acox-1* | 0.95 | 0.7481 | 0.46 | 0.0005 |
|  |  | *acox-2* | 1.06 | 0.6800 | 0.39 | <0.0001 |
|  |  | *acox-3* | 0.44 | 0.0079 | 0.29 | 0.0030 |
|  |  | *acox-5* | 2.21 | 0.0047 | 1.98 | 0.0054 |
|  |  | *F59F4.1* | 1.06 | 0.7617 | 1.03 | 0.8631 |
|  |  | *acox-1.4* | 2.05 | 0.4371 | 0.59 | 0.5411 |
|  | ACDH | *acdh-1* | 3.64 | 0.1152 | 0.00 | 0.0011 |
|  |  | *acdh-2* | 5.40 | <0.0001 | 0.01 | <0.0001 |
|  |  | *acdh-3* | 0.91 | 0.6486 | 0.78 | 0.2635 |
|  |  | *acdh-4* | 3.79 | 0.0305 | 3.69 | 0.0065 |
|  |  | *acdh-5* | 1.38 | 0.3684 | 2.80 | 0.0059 |
|  |  | *acdh-7* | 0.75 | 0.1195 | 1.21 | 0.2943 |
|  |  | *acdh-8* | 1.70 | 0.2604 | 2.21 | 0.0749 |
|  |  | *acdh-9* | 0.74 | 0.0604 | 0.35 | <0.0001 |
|  |  | *acdh-10* | 0.70 | 0.2358 | 1.35 | 0.3112 |
|  |  | *acdh-11* | 0.72 | 0.0453 | 0.98 | 0.8689 |
|  |  | *acdh-12* | 1.09 | 0.7599 | 0.82 | 0.4644 |
|  |  | *acdh-13* | 1.47 | 0.1193 | 0.58 | 0.0396 |
|  | ECH | *ech-1.2* | 1.22 | 0.6282 | 0.77 | 0.5283 |
|  |  | *ech-2* | 0.98 | 0.9299 | 0.83 | 0.5047 |
|  |  | *ech-3* | 0.88 | 0.5342 | 1.60 | 0.0322 |
|  |  | *ech-4* | 0.87 | 0.2735 | 0.92 | 0.4889 |
|  |  | *ech-5* | 0.83 | 0.5488 | 1.31 | 0.3719 |
|  |  | *ech-6* | 0.76 | 0.2708 | 0.32 | 0.0004 |
|  |  | *ech-7* | 1.41 | 0.2386 | 1.02 | 0.9570 |
|  |  | *ech-8* | 1.02 | 0.9348 | 0.67 | 0.0708 |
|  |  | *ech-9* | 1.67 | 0.3951 | 13.63 | 0.0001 |
|  | HACD | *hacd-1* | 0.72 | 0.1247 | 1.51 | 0.0573 |
|  |  | *B0272.3* | 0.67 | 0.1644 | 0.69 | 0.1903 |
|  |  | *F54C8.1* | 1.05 | 0.9031 | 2.41 | 0.0326 |
|  | Thiolase | *acaa-2* | 0.79 | 0.2925 | 0.65 | 0.0683 |
|  |  | *kat-1* | 1.01 | 0.8938 | 0.66 | 0.0014 |
|  |  | *B0303.3* | 1.09 | 0.6125 | 0.91 | 0.5761 |
|  |  | *T02G5.7* | 0.86 | 0.2258 | 0.71 | 0.0154 |
| Lipid transport | LBP | *lbp-1* | 0.87 | 0.4110 | 0.89 | 0.4607 |
|  |  | *lbp-2* | 0.80 | 0.3119 | 1.40 | 0.1304 |
|  |  | *lbp-3* | 0.87 | 0.4886 | 1.32 | 0.1729 |
|  |  | *lbp-4* | 0.98 | 0.9238 | 1.19 | 0.3101 |
|  |  | *lbp-5* | 1.66 | 0.0250 | 0.88 | 0.5197 |
|  |  | *lbp-6* | 1.06 | 0.7694 | 1.16 | 0.4611 |
|  |  | *lbp-7* | 0.53 | 0.0279 | 1.00 | 0.9920 |
|  |  | *lbp-9* | 0.85 | 0.7478 | 1.03 | 0.9509 |
|  | FAR | *far-1* | 1.00 | 0.9896 | 1.43 | 0.2254 |
|  |  | *far-2* | 1.03 | 0.8771 | 1.31 | 0.1789 |
|  |  | *far-3* | 0.16 | 0.0002 | 0.22 | 0.0042 |
|  |  | *far-4* | 0.49 | 0.0236 | 0.40 | 0.0248 |
|  |  | *far-5* | 0.77 | 0.3933 | 1.20 | 0.5441 |
|  |  | *far-6* | 1.22 | 0.7027 | 1.70 | 0.2809 |
|  |  | *far-7* | 0.59 | 0.3125 | 3.65 | 0.0186 |

GPI: glucose-6-phosphate isomerase; PFK: phospho-fructo-kinase; TPI: triosephosphate isomerase; GAPDH: glyceraldehyde 3-phosphate dehydrogenase; PGK: phosphoglycerate kinase; PGM: phosphoglycerate mutase; PK: pyruvate kinase; CS: citrate synthase; IDH: isocitrate dehydrogenase; α-KGDH: α-ketoglutarate dehydrogenase; SCS: succinyl-CoA synthetase; SDH: succinate dehydrogenase; MDH: malate dehydrogenase; ACC: acetyl-CoA carboxylase; FAS: fatty acid synthase; FAT: fatty acid desaturase; ELO: fatty acid elongase; LBP: lipid-binding protein; FAR: fatty acid- and retinol-binding protein; ACS: acyl-CoA synthetase; CPT: carnitine palmitoyl transferase; ACO: acyl-CoA oxidase; ACDH: acyl-CoA dehydrogenase; ECH: enoyl-CoA hydratase; HACD: hydroxyacyl-CoA dehydrogenase
