# Supplementary material for: MDT-15/MED15 permits longevity at low temperature via enhancing lipidostasis and proteostasis
Source: PLoS Biol. 2019 Aug 13;17(8):e3000415. doi: 10.1371/journal.pbio.3000415 (PMC6692015; doi:10.1371/journal.pbio.3000415)
Supplement: S4 Table — OA, oleic acid. (DOCX) [file pbio.3000415.s010.docx]

**S4 Table.** Statistical analysis and additional repeats of lifespan assays with or without oleic acid (OA) supplementation

| Strain/treatment | Mean lifespan ±s.e.m. (days) | 75th percentile | % change^Δ^ | Number of animals that died/total | *p* value vs. control | Figure in text |
| --- | --- | --- | --- | --- | --- | --- |
| Wild-type/15°C control^#^ | 31.1±0.8 | 35 |  | 52/90 |  | Fig 7G |
| Wild-type/15°C OA^#^ | 29.6±0.7 | 30 | -5% | 62/120 | 0.1442 | Fig 7G |
| *mdt-15(tm2182)*/15°C control^#^ | 16.0±0.4 | 16 | -49% | 107/120 | <0.0001 | Fig 7G |
| *mdt-15(tm2182)*/15°C OA^#^ | 22.6±0.5 | 23 | -27%  +41%^(^*^mdt-15(-)^* ^Ctrl)^ | 75/120 | <0.0001  <0.0001^(^*^mdt-15(-)^* ^Ctrl)^ | Fig 7G |
| Wild-type/25°C control^#^ | 14.0±0.3 | 15 |  | 95/120 |  |  |
| Wild-type/25°C OA^#^ | 12.0±0.3 | 14 | -14% | 104/120 | <0.0001 |  |
| *mdt-15(tm2182)*/25°C control^#^ | 10.1±0.2 | 11 | -28% | 119/120 | <0.0001 |  |
| *mdt-15(tm2182)*/25°C OA^#^ | 10.2±0.2 | 11 | -27%  +1%^(^*^mdt-15(-)^* ^Ctrl)^ | 120/121 | <0.0001  0.9516^(^*^mdt-15(-)^* ^Ctrl)^ |  |
| Wild-type/15°C control^#^ | 28.3±0.5 | 32 |  | 71/120 |  |  |
| Wild-type/15°C OA^#^ | 32.0±0.8 | 35 | +13% | 79/100 | <0.0001 |  |
| *mdt-15(tm2182)*/15°C control^#^ | 16.2±0.3 | 20 | -43% | 98/109 | <0.0001 |  |
| *mdt-15(tm2182)*/15°C OA^#^ | 22.5±0.5 | 27 | -20%  +39%^(^*^mdt-15(-)^* ^Ctrl)^ | 69/115 | <0.0001  <0.0001^(^*^mdt-15(-)^* ^Ctrl)^ |  |
| Wild-type/25°C control^#^ | 13.0±0.2 | 14 |  | 114/120 |  |  |
| Wild-type/25°C OA^#^ | 12.1±0.2 | 14 | -7% | 105/120 | 0.0042 |  |
| *mdt-15(tm2182)*/25°C control^#^ | 10.7±0.2 | 12 | -17.57% | 102/120 | <0.0001 |  |
| *mdt-15(tm2182)*/25°C OA^#^ | 11.0±0.2 | 12 | -15.50%  +3%^(^*^mdt-15(-)^* ^Ctrl)^ | 97/120 | <0.0001  0.5522^(^*^mdt-15(-)^* ^Ctrl)^ |  |
| Wild-type/15°C control | 25.2±0.7 | 27 |  | 49/120 |  |  |
| Wild-type/15°C OA | 27.1±0.6 | 32 | 7% | 89/120 | 0.014 |  |
| *mdt-15(tm2182)*/15°C control | 20.3±0.5 | 25 | -20% | 95/121 | <0.0001 |  |
| *mdt-15(tm2182)*/15°C OA | 24.0±0.8 | 29 | -5%  +18%^(^*^mdt-15(-)^* ^Ctrl)^ | 57/120 | 0.4001  0.0001^(^*^mdt-15(-)^* ^Ctrl)^ |  |
| Wild-type/25°C control | 15.8±0.4 | 19 |  | 99/120 |  |  |
| Wild-type/25°C OA | 15.6±0.3 | 16 | -1% | 106/120 | 0.2771 |  |
| *mdt-15(tm2182)*/25°C control | 11.3±0.2 | 12 | -29% | 104/120 | <0.0001 |  |
| *mdt-15(tm2182)*/25°C OA | 11.8±0.2 | 12 | -25%  +5%^(^*^mdt-15(-)^* ^Ctrl)^ | 67/120 | <0.0001  0.0691^(^*^mdt-15(-)^* ^Ctrl)^ |  |
| Wild-type/15°C control | 27.3±0.7 | 35 |  | 71/120 |  |  |
| Wild-type/15°C OA | 30.5±0.6 | 35 | +12% | 90/120 | 0.0002 |  |
| *mdt-15(tm2182)*/15°C control | 19.1±0.4 | 22 | -30% | 115/120 | <0.0001 |  |
| *mdt-15(tm2182)*/15°C OA | 22.2±0.5 | 30 | -19%  +16%^(^*^mdt-15(-)^* ^Ctrl)^ | 109/120 | <0.0001  <0.0001^(^*^mdt-15(-)^* ^Ctrl)^ |  |
| Wild-type/25°C control | 12.1±0.3 | 15 |  | 107/120 |  |  |
| Wild-type/25°C OA | 12.9±0.3 | 15 | +6% | 98/120 | 0.0814 |  |
| *mdt-15(tm2182)*/25°C control | 12.2±0.2 | 16 | 0% | 111/120 | 0.5927 |  |
| *mdt-15(tm2182)*/25°C OA | 12.3±0.2 | 14 | +1%  +1%^(^*^mdt-15(-)^* ^Ctrl)^ | 99/120 | 0.6684  0.6903^(^*^mdt-15(-)^* ^Ctrl)^ |  |

Lifespan assays were performed with or without OA on the media with 0.1% NP-40-containing ethanol as a solvent (See the “Preparation of plates for oleic acid (OA) feeding assays” section in Materials and Methods for detail information).

Lifespan data within the solid lines are biological replicates and were performed at the same time. Bold dashed lines distinguish lifespan data obtained from different temperatures. All *p* values were calculated within the individual sets by using the log-rank (Mantel-Cox) method.

Percent (%) changes and *p* values were calculated against the first column within bold dashed lines in the same experimental set.

*^mdt-15(-)^* ^Ctrl^: percent (%) changes and *p* values for OA feeding conditions in *mdt-15(−)* mutants were calculated against the control at each temperature in the same genetic background and experimental sets.

^#^ indicates the lifespan assays that were performed by using adult worms that were synchronized from eggs.
