# Supplementary material for: MDT-15/MED15 permits longevity at low temperature via enhancing lipidostasis and proteostasis
Source: PLoS Biol. 2019 Aug 13;17(8):e3000415. doi: 10.1371/journal.pbio.3000415 (PMC6692015; doi:10.1371/journal.pbio.3000415)
Supplement: S5 Table — (DOCX) [file pbio.3000415.s011.docx]

**S5 Table.** Statistical analysis and additional repeats of paralysis assays.

| Strain/treatment | Mean motility (not paralyzed) ±s.e.m. (days) | 75th percentile | % change^Δ^ | Number of animals that paralyzed/total | *p* value vs. control | Figure in text |
| --- | --- | --- | --- | --- | --- | --- |
| *polyQ::YFP*/15°C control | 21.7±0.4 | 26 |  | 101/115 |  | Fig 7E |
| *polyQ::YFP*/15°C OA | 22.1±0.5 | 26 | +2% | 84/120 | 0.2615 | Fig 7E |
| *mdt-15(tm2182); polyQ::YFP*/15°C control | 15.0±0.4 | 17 | -31% | 95/120 | <0.0001 | Fig 7E |
| *mdt-15(tm2182); polyQ::YFP*/15°C OA | 18.0±0.3 | 21 | -17%  +20%^(^*^mdt-15(-)^* ^Ctrl)^ | 83/120 | <0.0001  <0.0001^(^*^mdt-15(-)^* ^Ctrl)^ | Fig 7E |
| *polyQ::YFP*/25°C control | 12.5±0.3 | 14 |  | 99/120 |  |  |
| *polyQ::YFP*/25°C OA | 12.2±0.4 | 14 | -3% | 42/120 | 0.6576 |  |
| *mdt-15(tm2182); polyQ::YFP*/25°C control | 7.2±0.2 | 7 | -43% | 116/120 | <0.0001 |  |
| *mdt-15(tm2182); polyQ::YFP*/25°C OA | 8.8±0.3 | 10 | -30%  +23%^(^*^mdt-15(-)^* ^Ctrl)^ | 107/120 | <0.0001  <0.0001^(^*^mdt-15(-)^* ^Ctrl)^ |  |
| *polyQ::YFP*/15°C control | 16.6±0.3 | 19 |  | 96/120 |  |  |
| *polyQ::YFP*/15°C OA | 17.0±0.3 | 19 | +2% | 100/120 | 0.3299 |  |
| *mdt-15(tm2182); polyQ::YFP*/15°C control | 13.0±0.4 | 14 | -21% | 77/121 | <0.0001 |  |
| *mdt-15(tm2182); polyQ::YFP*/15°C OA | 14.5±0.4 | 18 | -13%  +11%^(^*^mdt-15(-)^* ^Ctrl)^ | 66/90 | <0.0001  0.0129^(^*^mdt-15(-)^* ^Ctrl)^ |  |
| *polyQ::YFP*/25°C control | 7.3±0.2 | 8 |  | 87/120 |  |  |
| *polyQ::YFP*/25°C OA | 8.0±0.2 | 10 | +9% | 72/120 | 0.0243 |  |
| *mdt-15(tm2182); polyQ::YFP*/25°C control | 8.1±0.2 | 9 | +10% | 84/120 | 0.0031 |  |
| *mdt-15(tm2182); polyQ::YFP*/25°C OA | 7.5±0.1 | 9 | +2%  -7%^(^*^mdt-15(-)^* ^Ctrl)^ | 104/120 | 0.1326  0.0047^(^*^mdt-15(-)^* ^Ctrl)^ |  |
| *polyQ::YFP*/15°C control | 23.2±0.4 | 25 |  | 110/120 |  |  |
| *polyQ::YFP*/15°C OA | 22.6±0.4 | 25 | -2% | 105/120 | 0.9809 |  |
| *mdt-15(tm2182); polyQ::YFP*/15°C control | 14.1±0.3 | 14 | -39% | 113/120 | <0.0001 |  |
| *mdt-15(tm2182); polyQ::YFP*/15°C OA | 14.2±0.3 | 18 | -39%  +1%^(^*^mdt-15(-)^* ^Ctrl)^ | 112/120 | <0.0001  0.0011^(^*^mdt-15(-)^* ^Ctrl)^ |  |
| *polyQ::YFP*/25°C control | 13.3±0.4 | 16 |  | 104/120 |  |  |
| *polyQ::YFP* /25°C OA | 12.9±0.3 | 16 | -4% | 120/120 | 0.171 |  |
| *mdt-15(tm2182); polyQ::YFP*/25°C control | 9. 7±0.3 | 12 | -27% | 109/120 | <0.0001 |  |
| *mdt-15(tm2182); polyQ::YFP*/25°C OA | 10.6±0.3 | 12 | -21%  +9%^(^*^mdt-15(-)^* ^Ctrl)^ | 109/120 | <0.0001  0.023^(^*^mdt-15(-)^* ^Ctrl)^ |  |
| *polyQ::YFP*/15°C control | 20. 8±0.4 | 23 |  | 92/120 |  | Fig 6C |
| *polyQ::YFP*/15°C OA | 21.2±0.4 | 23 | +2% | 80/119 | 0.4377 |  |
| *mdt-15(tm2182); polyQ::YFP*/15°C control | 15.2±0.3 | 19 | -27% | 81/109 | <0.0001 | Fig 6C |
| *mdt-15(tm2182); polyQ::YFP*/15°C OA | 17.5±0.4 | 19 | -16%  +15%^(^*^mdt-15(-)^* ^Ctrl)^ | 92/120 | <0.0001  <0.0001^(^*^mdt-15(-)^* ^Ctrl)^ |  |
| *polyQ::YFP*/25°C control | 9.1±0.2 | 11 |  | 87/120 |  | Fig 6C |
| *polyQ::YFP*/25°C OA | 9.3±0.2 | 11 | +1% | 101/120 | 0.9739 |  |
| *mdt-15(tm2182); polyQ::YFP*/25°C control | 7.7±0.2 | 9 | -16% | 89/113 | <0.0001 | Fig 6C |
| *mdt-15(tm2182); polyQ::YFP*/25°C OA | 8.3±0.2 | 9 | -9%  +8%^(^*^mdt-15(-)^* ^Ctrl)^ | 98/120 | 0.0003  0.0067^(^*^mdt-15(-)^* ^Ctrl)^ |  |
| *polyQ::YFP*/15°C control^#^ | 24.7±0.4 | 30 |  | 94/118 |  |  |
| *polyQ::YFP*/15°C OA^#^ | 24.0±0.5 | 30 | -3% | 86/120 | 0.3895 |  |
| *mdt-15(tm2182); polyQ::YFP*/15°C control^#^ | 15.5±0.4 | 20 | -37% | 108/120 | <0.0001 |  |
| *mdt-15(tm2182); polyQ::YFP*/15°C OA^#^ | 20.9±0.5 | 23 | -15%  +35%^(^*^mdt-15(-)^* ^Ctrl)^ | 106/120 | <0.0001  <0.0001^(^*^mdt-15(-)^* ^Ctrl)^ |  |
| *polyQ::YFP*/25°C control^#^ | 11.5±0.3 | 14 |  | 107/120 |  |  |
| *polyQ::YFP*/25°C OA^#^ | 12.2±0.3 | 14 | +6% | 87/120 | 0.0312 |  |
| *mdt-15(tm2182); polyQ::YFP*/25°C control^#^ | 8.4±0.3 | 11 | -27% | 84/84 | <0.0001 |  |
| *mdt-15(tm2182); polyQ::YFP*/25°C OA^#^ | 7.9±0.2 | 11 | -32%  -6%^(^*^mdt-15(-)^* ^Ctrl)^ | 89/101 | <0.0001  0.0939^(^*^mdt-15(-)^* ^Ctrl)^ |  |
| *polyQ::YFP*/15°C control^#^ | 18.8±0.4 | 21 |  | 106/120 |  |  |
| *polyQ::YFP*/15°C OA^#^ | 21.9±0.4 | 24 | +16% | 100/120 | <0.0001 |  |
| *mdt-15(tm2182); polyQ::YFP*/15°C control^#^ | 14.6±0.4 | 19 | -23% | 101/122 | <0.0001 |  |
| *mdt-15(tm2182); polyQ::YFP*/15°C OA^#^ | 16.3±0.4 | 20 | -13%  +12%^(^*^mdt-15(-)^* ^Ctrl)^ | 103/120 | <0.0001  <0.0001^(^*^mdt-15(-)^* ^Ctrl)^ |  |
| *polyQ::YFP*/25°C control^#^ | 10.1±0.3 | 12 |  | 111/120 |  |  |
| *polyQ::YFP*/25°C OA^#^ | 10.2±0.3 | 12 | +1% | 94/120 | 0.5396 |  |
| *mdt-15(tm2182); polyQ::YFP*/25°C control^#^ | 7.1±0.2 | 9 | -30% | 102/120 | <0.0001 |  |
| *mdt-15(tm2182); polyQ::YFP*/25°C OA^#^ | 7.8±0.2 | 9 | -22%  +11%^(^*^mdt-15(-)^* ^Ctrl)^ | 78/115 | <0.0001  0.0069^(^*^mdt-15(-)^* ^Ctrl)^ |  |
| *Aβ*/15°C control | 19.9±0.4 | 20 |  | 91/120 |  | Fig 7F |
| *Aβ*/15°C OA | 20.8±0.3 | 24 | +5% | 86/120 | 0.0091 | Fig 7F |
| *nhr-49(gk405); Aβ*/15°C control | 15.3±0.3 | 16 | -23% | 108/120 | <0.0001 | Fig 7F |
| *nhr-49(gk405); Aβ*/15°C OA | 18.5±0.3 | 20 | -7%  +21%^(^*^nhr-49(-)^* ^Ctrl)^ | 87/120 | 0.0362  <0.0001^(^*^nhr-49(-)^* ^Ctrl)^ | Fig 7F |
| *Aβ*/25°C control | 5.7±0.1 | 7 |  | 113/120 |  |  |
| *Aβ*/25°C OA | 5.3±0.1 | 7 | -8% | 120/120 | 0.0437 |  |
| *nhr-49(gk405); Aβ*/25°C control | 4.6±0.1 | 5 | -19% | 107/110 | <0.0001 |  |
| *nhr-49(gk405); Aβ*/25°C OA | 4.2±0.1 | 5 | -26%  -8%^(^*^nhr-49(-)^* ^Ctrl)^ | 102/104 | <0.0001  0.0108^(^*^nhr-49(-)^* ^Ctrl)^ |  |
| *Aβ*/15°C control | 17.5±0.4 | 18 |  | 102/120 |  |  |
| *Aβ*/15°C OA | 20.3±0.4 | 22 | +16% | 106/120 | <0.0001 |  |
| *nhr-49(gk405); Aβ*/15°C control | 13.6±0.2 | 14 | -22% | 108/115 | <0.0001 |  |
| *nhr-49(gk405); Aβ*/15°C OA | 17.9±0.4 | 22 | +2%  +31%^(^*^nhr-49(-)^* ^Ctrl)^ | 84/106 | 0.4427  <0.0001^(^*^nhr-49(-)^* ^Ctrl)^ |  |
| *Aβ*/25°C control | 2.6±0.2 | 3 |  | 109/116 |  |  |
| *Aβ*/25°C OA | 2.2±0.2 | 3 | -14% | 124/128 | 0.0549 |  |
| *nhr-49(gk405); Aβ*/25°C control | 1.7±0.1 | 2 | -33% | 96/97 | <0.0001 |  |
| *nhr-49(gk405); Aβ*/25°C OA | 1.8±0.1 | 2 | -29%  +6%^(^*^nhr-49(-)^* ^Ctrl)^ | 104/106 | 0.0002  0.6122^(^*^nhr-49(-)^* ^Ctrl)^ |  |

Paralysis assays were performed with or without OA on the media with 0.1% NP-40-containing ethanol as a solvent (See the “Preparation of plates for oleic acid (OA) feeding assays” section in Materials and Methods for detail information). *Q35::YFP* [*polyQ::YFP*] in wild-type and *mdt-15(−)* mutant backgrounds were used for the paralysis assays.

Paralysis assay data within the double-solid lines are same experimental sets and biological replicates are separated by solid lines. Paralysis assay data within the solid lines were performed at the same time. Bold dashed lines distinguish paralysis assay data from different temperatures. All *p* values were calculated within the individual sets by using the log-rank (Mantel-Cox) method.

Percent (%) changes and *p* values were calculated against the first column within bold dashed lines in the same experimental set.

*^mdt-15(-)^* ^Ctrl^ or *^nhr-49(-)^* ^Ctrl^: percent (%) changes and *p* values for OA feeding conditions in *mdt-15(−)* or *nhr-49(−)* mutants were calculated against the control at each temperature in the same genetic background and experimental sets.

^#^ indicates the paralysis assays that were performed by using adult worms that were synchronized from eggs.
