# Supplementary material for: MDT-15/MED15 permits longevity at low temperature via enhancing lipidostasis and proteostasis
Source: PLoS Biol. 2019 Aug 13;17(8):e3000415. doi: 10.1371/journal.pbio.3000415 (PMC6692015; doi:10.1371/journal.pbio.3000415)
Supplement: S6 Table — (DOCX) [file pbio.3000415.s012.docx]

**S6 Table.** The Cox proportional hazard regression analysis.

| Conditions | Risk factor | Coefficient | *exp(Coefficient)* | *p*-value | Figures in text |
| --- | --- | --- | --- | --- | --- |
| 15°C | *mdt-15(tm2182)* | 1.0100 | 2.7430 | <0.0001 | Fig 1A |
| 25°C | *mdt-15(tm2182)* | 0.4150 | 1.5150 | 0.0067 | Fig 1A |
| 15°C | *mdt-15(tm2182)* | 1.5500 | 4.7000 | <0.0001 |  |
| 25°C | *mdt-15(tm2182)* | 0.0010 | 1.0010 | 0.9949 |  |
| 15°C | *mdt-15(tm2182)* | 1.0200 | 2.7680 | <0.0001 |  |
| 25°C | *mdt-15(tm2182)* | 0.4710 | 1.6010 | 0.0013 |  |
| 15°C | *mdt-15::degron::EmGFP* | 2.1400 | 8.5240 | <0.0001 | Fig 1D |
| 25°C | *mdt-15::degron::EmGFP* | 0.4600 | 1.5840 | 0.0008 | Fig 1D |
| 15°C | *mdt-15::degron::EmGFP* | 0.8930 | 2.4420 | <0.0001 |  |
| 25°C | *mdt-15::degron::EmGFP* | 0.2730 | 1.3130 | 0.0450 |  |
| 15°C | *mdt-15::degron::EmGFP* | 1.0700 | 2.9040 | <0.0001 |  |
| 25°C | *mdt-15::degron::EmGFP* | 0.5930 | 1.8090 | <0.0001 |  |
| 15°C | *mdt-15(yh8)* | -0.6310 | 0.5320 | 0.0001 | Fig 1E |
| 25°C | *mdt-15(yh8)* | -0.2440 | 0.7830 | 0.1738 | Fig 1E |
| 15°C | *skn-1(zj15)* | 0.6230 | 1.8640 | 0.0000 | S4D Fig |
| 25°C | *skn-1(zj15)* | -0.5610 | 0.5710 | 0.0001 | S4D Fig |
| 15°C | *mdt-15(yh8)* | -0.1940 | 0.8240 | 0.2391 |  |
| 25°C | *mdt-15(yh8)* | 0.1900 | 1.2090 | 0.2709 |  |
| 15°C | *skn-1(zj15)* | 0.0697 | 1.0720 | 0.6010 |  |
| 25°C | *skn-1(zj15)* | -0.0693 | 0.9330 | 0.5935 |  |
| 15°C | *fat-6(tm331); fat-7(wa36)* | 1.5000 | 4.4620 | <0.0001 | Fig 4A |
| 25°C | *fat-6(tm331); fat-7(wa36)* | -0.3400 | 0.7120 | 0.0342 | Fig 4A |
| 15°C | *fat-6(tm331); fat-7(wa36)* | 1.0700 | 2.9100 | <0.0001 |  |
| 25°C | *fat-6(tm331); fat-7(wa36)* | 0.1040 | 1.1100 | 0.5188 |  |
| 15°C | *paqr-2(tm3410)* | 3.2600 | 26.1170 | <0.0001 | Fig 4B |
| 25°C | *paqr-2(tm3410)* | -0.1600 | 0.8520 | 0.2420 | Fig 4B |
| 15°C | *paqr-2(tm3410)* | 3.1500 | 23.3950 | <0.0001 |  |
| 25°C | *paqr-2(tm3410)* | 0.1580 | 1.1710 | 0.2768 |  |
| 15°C | *nhr-49(gk405)* | 2.4200 | 11.2250 | <0.0001 | Fig 4C |
| 25°C | *nhr-49(gk405)* | 0.2720 | 1.3120 | 0.0566 | Fig 4C |
| 15°C | *nhr-49(gk405)* | 1.6100 | 5.0110 | <0.0001 |  |
| 25°C | *nhr-49(gk405)* | 0.5800 | 1.7870 | 0.0001 |  |
| 15°C | *mdt-15(tm2182)* | 1.5900 | 4.8870 | <0.0001 | Fig 4D |
| 15°C | *nhr-49(gk405)* | 1.8600 | 6.4410 | <0.0001 | Fig 4D |
| 15°C | *mdt-15(tm2182)* | 0.8700 | 2.3860 | <0.0001 |  |
| 15°C | *nhr-49(gk405)* | 0.8710 | 2.3900 | <0.0001 |  |
| 15°C | Glucose | 2.2700 | 9.6330 | <0.0001 | Fig 4E |
| 25°C | Glucose | 0.0576 | 1.0590 | 0.7052 | Fig 4E |
| 15°C | Glucose | 2.4900 | 12.0930 | <0.0001 |  |
| 25°C | Glucose | 0.4690 | 1.5980 | 0.0007 |  |
| 15°C | Glucose Kan * | 4.2200 | 68.2550 | <0.0001 | Fig 4F |
| 25°C | Glucose Kan * | 0.6320 | 1.8810 | <0.0001 | Fig 4F |
| 15°C | Glucose Kan * | 2.7200 | 15.1660 | <0.0001 |  |
| 25°C | Glucose Kan * | 0.6980 | 2.0090 | <0.0001 |  |
| 15°C | *mdt-15(tm2182)* ^#^ | 3.0600 | 21.3260 | <0.0001 | Fig 7G |
| 15°C | OA^#^ | -1.0200 | 0.3620 | <0.0001 | Fig 7G |
| 25°C | *mdt-15(tm2182)* ^#^ | 1.3200 | 3.7360 | <0.0001 | Fig 7G |
| 25°C | OA^#^ | 0.3530 | 1.4230 | 0.0004 | Fig 7G |
| 15°C | *mdt-15(tm2182)* ^#^ | 2.5600 | 12.9830 | <0.0001 |  |
| 15°C | OA^#^ | -1.1400 | 0.3210 | <0.0001 |  |
| 25°C | *mdt-15(tm2182)* ^#^ | 0.7400 | 2.0960 | <0.0001 |  |
| 25°C | OA^#^ | 0.1720 | 1.1880 | 0.0817 |  |
| 15°C | *mdt-15(tm2182)* | 0.8420 | 2.3220 | <0.0001 |  |
| 15°C | OA | -0.6460 | 0.5240 | <0.0001 |  |
| 25°C | *mdt-15(tm2182)* | 2.0000 | 7.3870 | <0.0001 |  |
| 25°C | OA | 0.0032 | 1.0030 | 0.9760 |  |
| 15°C | *mdt-15(tm2182)* | 1.4800 | 4.4090 | <0.0001 |  |
| 15°C | OA | -0.5560 | 0.5740 | <0.0001 |  |
| 25°C | *mdt-15(tm2182)* | 0.1900 | 1.2100 | 0.0641 |  |
| 25°C | OA | -0.0671 | 0.9350 | 0.4979 |  |
| 15°C | *mdt-15(tm2182)* * | 2.0400 | 7.6580 | <0.0001 | S1B Fig |
| 25°C | *mdt-15(tm2182)* * | 0.2060 | 1.2280 | 0.1212 | S1B Fig |
| 15°C | *mdt-15(tm2182)* * | 1.3100 | 3.7150 | <0.0001 |  |
| 25°C | *mdt-15(tm2182)* * | -0.0448 | 0.9560 | 0.7748 |  |
| 15°C | *eft-3p::TIR1::mRuby* | -1.0600 | 0.3450 | <0.0001 | S1E Fig |
| 25°C | *eft-3p::TIR1::mRuby* | -0.5320 | 0.5870 | 0.0001 | S1E Fig |
| 15°C | *eft-3p::TIR1::mRuby* | -0.7550 | 0.4700 | <0.0001 |  |
| 25°C | *eft-3p::TIR1::mRuby* | -0.3600 | 0.6970 | 0.0085 |  |
| 15°C | *eft-3p::TIR1::mRuby* | -0.4450 | 0.6410 | 0.0011 |  |
| 25°C | *eft-3p::TIR1::mRuby* | -1.4400 | 0.2360 | <0.0001 |  |
| 15°C | *sbp-1* RNAi * | 0.2740 | 1.3150 | 0.1489 | S4E Fig |
| 25°C | *sbp-1* RNAi * | 0.8970 | 2.4520 | 0.1566 | S4E Fig |
| 15°C | *sbp-1* RNAi * | 0.2500 | 1.2840 | 0.1454 |  |
| 25°C | *sbp-1* RNAi * | 1.5000 | 4.4990 | 0.1523 |  |

Data for performing the Cox proportional hazard regression analysis within the double-solid lines are the same experimental sets and biological replicates are separated by solid lines. The data within the solid lines were performed at the same time. The results of the Cox proportional hazard regression analysis were calculated using partial likelihood estimator.

* represents temperature shift experiments from 20°C to indicated temperatures at L4 stage.

^#^ indicates the lifespan assays that were performed by using adult worms that were synchronized from eggs.
