# Supplementary material for: MDT-15/MED15 permits longevity at low temperature via enhancing lipidostasis and proteostasis
Source: PLoS Biol. 2019 Aug 13;17(8):e3000415. doi: 10.1371/journal.pbio.3000415 (PMC6692015; doi:10.1371/journal.pbio.3000415)
Supplement: S7 Table — (DOCX) [file pbio.3000415.s013.docx]

**S7 table.** Standard deviations for box plot data

| **Assays** | **Target genes/**  **fatty acids** | **Conditions** | **Genotypes** | **Mean± standard deviation** | ***p*-value** | **Figures in text** |
| --- | --- | --- | --- | --- | --- | --- |
| Relative body area |  | 25°C | *mdt-15(tm2182)*/ Wild-type | 0.81±0.07 |  | Fig 1F |
|  |  | 15°C | *mdt-15(tm2182)*/ Wild-type | 0.68±0.01 | 0.0318 | Fig 1F |
| Hatching (%) |  | 25°C | Wild-type | 98±2 |  | Fig 1H |
|  |  | 15°C | Wild-type | 97±3 | 0.3972 | Fig 1H |
|  |  | 25°C | *mdt-15(tm2182)* | 21±4 | <0.0001 | Fig 1H |
|  |  | 15°C | *mdt-15(tm2182)* | 3±2 | <0.0001  <0.0001 *^mdt-15(-)^* ^at 25°C^  <0.0001 ^Wild-type at 15°C^ | Fig 1H |
| qRT-PCR  (relative mRNA levels) | *fat-1* | 25°C | Wild-type | 1.0±0.0 |  | Fig 2B |
|  |  | 15°C | Wild-type | 0.76±0.09 | 0.0092 | Fig 2B |
|  |  | 25°C | *mdt-15(tm2182)* | 0.60±0.12 | 0.0039 | Fig 2B |
|  |  | 15°C | *mdt-15(tm2182)* | 0.51±0.01 | <0.0001  0.0083 ^Wild-type at 15°C^ | Fig 2B |
|  | *fat-2* | 25°C | Wild-type | 1.0±0.0 |  | Fig 2B |
|  |  | 15°C | Wild-type | 0.50±0.25 | <0.0001 | Fig 2B |
|  |  | 25°C | *mdt-15(tm2182)* | 0.84±0.32 | 0.1574 | Fig 2B |
|  |  | 15°C | *mdt-15(tm2182)* | 0.29±0.11 | <0.0001  0.0383 ^Wild-type at 15°C^ | Fig 2B |
|  | *fat-3* | 25°C | Wild-type | 1.0±0.0 |  | Fig 2B |
|  |  | 15°C | Wild-type | 1.16±0.28 | 0.3846 | Fig 2B |
|  |  | 25°C | *mdt-15(tm2182)* | 1.14±0.42 | 0.5959 | Fig 2B |
|  |  | 15°C | *mdt-15(tm2182)* | 1.45±0.51 | 0.1998  0.0803 ^Wild-type at 15°C^ | Fig 2B |
|  | *fat-4* | 25°C | Wild-type | 1.0±0.0 |  | Fig 2B |
|  |  | 15°C | Wild-type | 0.89±0.19 | 0.3591 | Fig 2B |
|  |  | 25°C | *mdt-15(tm2182)* | 1.11±0.33 | 0.6063 | Fig 2B |
|  |  | 15°C | *mdt-15(tm2182)* | 1.37±0.51 | 0.2755  0.1082 ^Wild-type at 15°C^ | Fig 2B |
|  | *fat-5* | 25°C | Wild-type | 1.0±0.0 |  | Fig 2B |
|  |  | 15°C | Wild-type | 1.08±0.39 | 0.5450 | Fig 2B |
|  |  | 25°C | *mdt-15(tm2182)* | 0.41±0.33 | 0.0001 | Fig 2B |
|  |  | 15°C | *mdt-15(tm2182)* | 0.02±0.02 | <0.0001  <0.0001 ^Wild-type at 15°C^ | Fig 2B |
|  | *fat-6* | 25°C | Wild-type | 1.0±0.0 |  | Fig 2B |
|  |  | 15°C | Wild-type | 0.77±0.50 | 0.1811 | Fig 2B |
|  |  | 25°C | *mdt-15(tm2182)* | 0.55±0.31 | 0.0005 | Fig 2B |
|  |  | 15°C | *mdt-15(tm2182)* | 0.27±0.10 | <0.0001  0.0092 ^Wild-type at 15°C^ | Fig 2B |
|  | *fat-7* | 25°C | Wild-type | 1.0±0.0 |  | Fig 2B |
|  |  | 15°C | Wild-type | 28.64±6.07 | 0.0014 | Fig 2B |
|  |  | 25°C | *mdt-15(tm2182)* | 0.01±0.01 | <0.0001 | Fig 2B |
|  |  | 15°C | *mdt-15(tm2182)* | 0.28±0.32 | 0.0174  0.0013 ^Wild-type at 15°C^ | Fig 2B |
| *fat-7::GFP* expression  (arbitrary units) |  | 25°C | Wild-type | 5.03±2.98 |  | Fig 2C |
|  |  | 15°C | Wild-type | 22.27±5.66 | <0.0001 | Fig 2C |
|  |  | 25°C | *mdt-15(tm2182)* | 0.85±0.34 | <0.0001 | Fig 2C |
|  |  | 15°C | *mdt-15(tm2182)* | 0.57±0.29 | <0.0001  0.0075 *^mdt-15(-)^* ^at 25°C^  <0.0001 ^Wild-type at 15°C^ | Fig 2C |
| Oil red O intensity (arbitrary unit) |  | 25°C | Wild-type | 0.14±0.07 |  | Fig 3B |
|  |  | 15°C | Wild-type | 0.16±0.10 | 0.4563 | Fig 3B |
|  |  | 25°C | *mdt-15(tm2182)* | 0.10±0.04 | 0.0477 | Fig 3B |
|  |  | 15°C | *mdt-15(tm2182)* | 0.04±0.03 | <0.0001  <0.0001 *^mdt-15(-)^* ^at 25°C^  <0.0001 ^Wild-type at 15°C^ | Fig 3B |
| Fatty acids (%) | C14:0 | 25°C | Wild-type | 1.32±0.11 |  | Fig 3D |
|  |  | 15°C | Wild-type | 0.86±0.05 | 0.0001 | Fig 3D |
|  | C15:iso | 25°C | Wild-type | 5.17±0.16 |  | Fig 3D |
|  |  | 15°C | Wild-type | 2.84±0.11 | <0.0001 | Fig 3D |
|  | C16:0 | 25°C | Wild-type | 5.40±0.61 |  | Fig 3D |
|  |  | 15°C | Wild-type | 3.56±0.42 | 0.0005 | Fig 3D |
|  | C16:1n-7 | 25°C | Wild-type | 2.96±0.15 |  | Fig 3D |
|  |  | 15°C | Wild-type | 3.69±0.16 | 0.0001 | Fig 3D |
|  | 17:iso | 25°C | Wild-type | 3.76±0.23 |  | Fig 3D |
|  |  | 15°C | Wild-type | 2.52±0.11 | <0.0001 | Fig 3D |
|  | C17:0 | 25°C | Wild-type | 5.14±1.67 |  | Fig 3D |
|  |  | 15°C | Wild-type | 3.60±1.10 | 0.1230 | Fig 3D |
|  | C17:cyc | 25°C | Wild-type | 19.18±2.14 |  | Fig 3D |
|  |  | 15°C | Wild-type | 16.72±1.51 | 0.0688 | Fig 3D |
|  | C18:0 | 25°C | Wild-type | 3.83±0.41 |  | Fig 3D |
|  |  | 15°C | Wild-type | 4.28±0.56 | 0.1845 | Fig 3D |
|  | C18:1n-9 | 25°C | Wild-type | 2.15±1.03 |  | Fig 3D |
|  |  | 15°C | Wild-type | 2.04±0.50 | 0.8354 | Fig 3D |
|  | C18:1n-7 | 25°C | Wild-type | 21.01±1.13 |  | Fig 3D |
|  |  | 15°C | Wild-type | 26.60±2.03 | 0.0007 | Fig 3D |
|  | C18:2n-6 | 25°C | Wild-type | 3.72±0.48 |  | Fig 3D |
|  |  | 15°C | Wild-type | 3.42±0.65 | 0.4261 | Fig 3D |
|  | C18:3n-6 | 25°C | Wild-type | 2.41±0.34 |  | Fig 3D |
|  |  | 15°C | Wild-type | 2.34±0.25 | 0.7231 | Fig 3D |
|  | C19:cyc | 25°C | Wild-type | 4.94±0.46 |  | Fig 3D |
|  |  | 15°C | Wild-type | 5.87±0.35 | 0.0066 | Fig 3D |
|  | C20:3n-6 | 25°C | Wild-type | 3.35±0.26 |  | Fig 3D |
|  |  | 15°C | Wild-type | 3.65±0.25 | 0.1017 | Fig 3D |
|  | C20:4n-6 | 25°C | Wild-type | 1.44±0.12 |  | Fig 3D |
|  |  | 15°C | Wild-type | 1.47±0.36 | 0.8703 | Fig 3D |
|  | C20:4n-3 | 25°C | Wild-type | 3.63±0.41 |  | Fig 3D |
|  |  | 15°C | Wild-type | 4.50±0.53 | 0.0196 | Fig 3D |
|  | C20:5n-3 | 25°C | Wild-type | 10.57±1.32 |  | Fig 3D |
|  |  | 15°C | Wild-type | 12.21±1.29 | 0.0838 | Fig 3D |
| Fatty acids (%) | Saturated | 25°C | Wild-type | 15.69±0.66 |  | Fig 3E |
|  |  | 15°C | Wild-type | 12.12±0.80 | 0.0001 | Fig 3E |
|  |  | 25°C | *mdt-15(tm2182)* | 20.24±2.13 | 0.0018 | Fig 3E |
|  |  | 15°C | *mdt-15(tm2182)* | 18.48±0.42 | <0.0001  0.1076 *^mdt-15(-)^* ^at 25°C^  <0.0001 ^Wild-type at 15°C^ | Fig 3E |
|  | Unsaturated | 25°C | Wild-type | 51.26±1.74 |  | Fig 3F |
|  |  | 15°C | Wild-type | 59.94±1.64 | <0.0001 | Fig 3F |
|  |  | 25°C | *mdt-15(tm2182)* | 43.33±1.46 | 0.0001 | Fig 3F |
|  |  | 15°C | *mdt-15(tm2182)* | 46.44±1.89 | 0.0030  0.0194 *^mdt-15(-)^* ^at 25°C^  <0.0001 ^Wild-type at 15°C^ | Fig 3F |
|  | Unsaturated/saturated | 25°C | Wild-type | 3.27±0.14 |  | Fig 3G |
|  |  | 15°C | Wild-type | 5.21±0.36 | <0.0001 | Fig 3G |
|  |  | 25°C | *mdt-15(tm2182)* | 2.25±0.17 | <0.0001 | Fig 3G |
|  |  | 15°C | *mdt-15(tm2182)* | 2.61±0.16 | <0.0001  0.0086 *^mdt-15(-)^* ^at 25°C^  <0.0001 ^Wild-type at 15°C^ | Fig 3G |
| RNA-seq. (FPKM) | *hsp-16.1* | 25°C | Wild-type | 33.64±11.52 |  | Fig 5A |
|  |  | 15°C | Wild-type | 2.14±0.56 | 0.0091 | Fig 5A |
|  |  | 25°C | *mdt-15(tm2182)* | 59.14±10.86 | 0.0493 | Fig 5A |
|  |  | 15°C | *mdt-15(tm2182)* | 211.44±71.26 | 0.0130  0.0216 *^mdt-15(-)^* ^at 25°C^  0.0070 ^Wild-type at 15°C^ | Fig 5A |
|  | *hsp-16.11* | 25°C | Wild-type | 28.61±9.04 |  | Fig 5A |
|  |  | 15°C | Wild-type | 1.95±0.48 | 0.0070 | Fig 5A |
|  |  | 25°C | *mdt-15(tm2182)* | 52.23±9.33 | 0.0346 | Fig 5A |
|  |  | 15°C | *mdt-15(tm2182)* | 185.07±64.04 | 0.0138  0.0237 *^mdt-15(-)^* ^at 25°C^  0.0077 ^Wild-type at 15°C^ | Fig 5A |
|  | *hsp-16.49* | 25°C | Wild-type | 32.32±9.37 |  | Fig 5A |
|  |  | 15°C | Wild-type | 1.85±0.43 | 0.0049 | Fig 5A |
|  |  | 25°C | *mdt-15(tm2182)* | 53.80±13.13 | 0.0825 | Fig 5A |
|  |  | 15°C | *mdt-15(tm2182)* | 131.44±47.07 | 0.0232  0.0513 *^mdt-15(-)^* ^at 25°C^  0.0089 ^Wild-type at 15°C^ | Fig 5A |
|  | *hsp-16.48* | 25°C | Wild-type | 31.01±8.45 |  | Fig 5A |
|  |  | 15°C | Wild-type | 1.83±0.49 | 0.0039 | Fig 5A |
|  |  | 25°C | *mdt-15(tm2182)* | 51.97±12.25 | 0.0712 | Fig 5A |
|  |  | 15°C | *mdt-15(tm2182)* | 125.29±44.11 | 0.0220  0.0501 *^mdt-15(-)^* ^at 25°C^  0.0084 ^Wild-type at 15°C^ | Fig 5A |
|  | *hsp-16.41* | 25°C | Wild-type | 5.00±1.59 |  | Fig 5A |
|  |  | 15°C | Wild-type | 0.30±0.24 | 0.0072 | Fig 5A |
|  |  | 25°C | *mdt-15(tm2182)* | 5.73±1.06 | 0.5431 | Fig 5A |
|  |  | 15°C | *mdt-15(tm2182)* | 22.03±8.64 | 0.0283  0.0316 *^mdt-15(-)^* ^at 25°C^  0.0121 ^Wild-type at 15°C^ | Fig 5A |
|  | *hsp-16.2* | 25°C | Wild-type | 2.98±0.44 |  | Fig 5A |
|  |  | 15°C | Wild-type | 0.38±0.02 | 0.0005 | Fig 5A |
|  |  | 25°C | *mdt-15(tm2182)* | 4.30±0.99 | 0.1024 | Fig 5A |
|  |  | 15°C | *mdt-15(tm2182)* | 17.72±6.58 | 0.0180  0.0250 *^mdt-15(-)^* ^at 25°C^  0.0103 ^Wild-type at 15°C^ | Fig 5A |
|  | *F44E5.4 (Hsp70)* | 25°C | Wild-type | 3.57±2.49 |  | Fig 5A |
|  |  | 15°C | Wild-type | 0.53±0.46 | 0.1058 | Fig 5A |
|  |  | 25°C | *mdt-15(tm2182)* | 4.45±0.57 | 0.5816 | Fig 5A |
|  |  | 15°C | *mdt-15(tm2182)* | 9.27±2.24 | 0.0419  0.0225 *^mdt-15(-)^* ^at 25°C^  0.0027 ^Wild-type at 15°C^ | Fig 5A |
|  | *F44E5.5 (Hsp70)* | 25°C | Wild-type | 3.54±2.49 |  | Fig 5A |
|  |  | 15°C | Wild-type | 0.53±0.46 | 0.1083 | Fig 5A |
|  |  | 25°C | *mdt-15(tm2182)* | 4.13±0.34 | 0.7062 | Fig 5A |
|  |  | 15°C | *mdt-15(tm2182)* | 9.00±2.26 | 0.0482  0.0210 *^mdt-15(-)^* ^at 25°C^  0.0031 ^Wild-type at 15°C^ | Fig 5A |
| qRT-PCR  (relative mRNA levels) | *hsp-16.1/11* | 15°C | Wild-type | 1.0±0.0 |  | Fig 5C |
|  |  | 15°C | *mdt-15(tm2182)* | 102.12±84.81 | 0.0088 | Fig 5C |
|  | *F44E5.5 (Hsp70)* | 15°C | Wild-type | 1.0±0.0 |  | Fig 5D |
|  |  | 15°C | *mdt-15(tm2182)* | 6.19±4.06 | 0.0114 | Fig 5D |
|  | *hsp-16.41* | 15°C | Wild-type | 1.0±0.0 |  | Fig 5E |
|  |  | 15°C | *mdt-15(tm2182)* | 115.61±128.67 | 0.1977 | Fig 5E |
| *hsp-16.1::GFP* expression  (arbitrary units) |  | 15°C | Control RNAi | 0.31±0.12 |  | Fig 5H |
|  |  | 15°C | *fat-6/7* RNAi | 6.05±3.29 | <0.0001 | Fig 5H |
| qRT-PCR  (relative mRNA levels) | *hsp-16.1/11* | 15°C | Wild-type | 1.0±0.0 |  | Fig 5I |
|  |  | 15°C | *paqr-2(tm3410)* | 7.27±2.24 | 0.0083 | Fig 5I |
| qRT-PCR  (relative mRNA levels) | *hsp-16.1/2/11* | 15°C | Wild-type Control RNAi | 1.0±0.0 |  | Fig 5J |
|  |  | 15°C | Wild-type *fat-6/7* RNAi | 195.48±203.57 | 0.0652 | Fig 5J |
|  |  | 15°C | Wild-type *mdt-15* RNAi | 139.42±100.06 | 0.0148 | Fig 5J |
|  | *hsp-16.41* | 15°C | Wild-type Control RNAi | 1.0±0.0 |  | Fig 5J |
|  |  | 15°C | Wild-type *fat-6/7* RNAi | 6.44±1.76 | 0.0002 | Fig 5J |
|  |  | 15°C | Wild-type *mdt-15* RNAi | 11.60±10.38 | 0.0518 | Fig 5J |
|  | *hsp-16.48/49* | 15°C | Wild-type Control RNAi | 1.0±0.0 |  | Fig 5J |
|  |  | 15°C | Wild-type *fat-6/7* RNAi | 248.12±227.36 | 0.0412 | Fig 5J |
|  |  | 15°C | Wild-type *mdt-15* RNAi | 226.36±149.47 | 0.0098 | Fig 5J |
|  | *hsp-70* | 15°C | Wild-type Control RNAi | 1.0±0.0 |  | Fig 5J |
|  |  | 15°C | Wild-type *fat-6/7* RNAi | 10.53±10.03 | 0.0672 | Fig 5J |
|  |  | 15°C | Wild-type *mdt-15* RNAi | 9.42±5.73 | 0.0111 | Fig 5J |
|  | *fat-6* | 15°C | Wild-type Control RNAi | 1.0±0.0 |  | S5D Fig |
|  |  | 15°C | Wild-type *fat-6/7* RNAi | 0.26±0.21 | 0.0005 | S5D Fig |
|  |  | 15°C | Wild-type *fat-6/7* RNAi* | 1.42±1.15 | 0.4919 | S5D Fig |
|  |  | 15°C | Wild-type *mdt-15* RNAi | 0.02±0.01 | <0.0001 | S5D Fig |
|  | *fat-7* | 15°C | Wild-type Control RNAi | 1.0±0.0 |  | S5D Fig |
|  |  | 15°C | Wild-type *fat-6/7* RNAi | 0.32±0.34 | 0.0021 | S5D Fig |
|  |  | 15°C | Wild-type *fat-6/7* RNAi* | 0.06±0.07 | <0.0001 | S5D Fig |
|  |  | 15°C | Wild-type *mdt-15* RNAi | 0.01±0.01 | <0.0001 | S5D Fig |
| Aggregates (number) |  | 15°C | *polyQ::YFP* | 84.36±24.60 |  | Fig 6B, Fig 7D |
|  |  | 15°C+OA | *polyQ::YFP* | 85.55±13.55 | 0.8204 | Fig 7D |
|  |  | 15°C | *mdt-15(tm2182); polyQ::YFP* | 99.87±14.41 | 0.0041 | Fig 6B, Fig 7D |
|  |  | 15°C+OA | *mdt-15(tm2182); polyQ::YFP* | 75.58±19.23 | 0.1232  <0.0001 *^mdt-15(tm2182)^* | Fig 7D |
|  |  | 15°C | *polyQ::YFP* Control RNAi | 52.72±19.34 |  | Fig 6F |
|  |  | 15°C | *polyQ::YFP* *mdt-15* RNAi | 65.39±10.24 | 0.0033 | Fig 6F |
|  |  | 15°C | *polyQ::YFP* *hsf-1* RNAi | 57.96±15.40 | 0.2638 | Fig 6F |
|  |  | 15°C | *polyQ::YFP* *mdt-15/hsf-1* RNAi | 84.50±11.81 | <0.0001  <0.0001 *^mdt-15^* ^RNAi^  <0.0001 *^hsf-1^* ^RNAi^ | Fig 6F |
| *hsp-16.1::GFP* expression  (arbitrary units) |  | 15°C | Wild-type | 0.13±0.04 |  | Fig 7B |
|  |  | 15°C+OA | Wild-type | 0.18±0.23 | 0.3205 | Fig 7B |
|  |  | 15°C | *mdt-15(tm2182)* | 7.92±6.73 | <0.0001 | Fig 7B |
|  |  | 15°C+OA | *mdt-15(tm2182)* | 2.82±1.49 | <0.0001  0.0007 *^mdt-15(tm2182)^* | Fig 7B |
| Body area (arbitrary unit) |  | 25°C | Wild-type | 169977±18313 |  | S1F Fig |
|  |  | 15°C | Wild-type | 164792±15844 | 0.2997 | S1F Fig |
|  |  | 25°C | *mdt-15(tm2182)* | 137569±11802 | <0.0001 | S1F Fig |
|  |  | 15°C | *mdt-15(tm2182)* | 112497±10774 | <0.0001  <0.0001 *^mdt-15(-)^* ^at 25°C^  <0.0001 ^Wild-type at 15°C^ | S1F Fig |
| Hatching (%) |  | 25°C | *mdt-15(tm2182)* | 25.74±6.21 |  | S1G Fig |
|  |  | 16°C | *mdt-15(tm2182)* | 0.85±0.85 | 0.0023 | S1G Fig |
| Number of progeny |  | 25°C | Wild-type | 166.98±45.20 |  | S1H Fig |
|  |  | 15°C | Wild-type | 283.90±30.33 | 0.0031 | S1H Fig |
|  |  | 25°C | *mdt-15(tm2182)* | 1.28±2.59 | <0.0001 | S1H Fig |
|  |  | 15°C | *mdt-15(tm2182)* | 2.04±1.96 | 0.0002  <0.0001 ^Wild-type at 15°C^  0.6429 *^mdt-15(-)^* ^at 25°C^ | S1H Fig |
| RNA-seq. (FPKM) | *fat-1* | 25°C | Wild-type | 202.24±139.45 |  | S3G Fig |
|  |  | 15°C | Wild-type | 163.98±63.77 | 0.6879 | S3G Fig |
|  |  | 25°C | *mdt-15(tm2182)* | 144.92±79.60 | 0.5698 | S3G Fig |
|  |  | 15°C | *mdt-15(tm2182)* | 87.62±33.83 | 0.2387  0.3152 *^mdt-15(-)^* ^at 25°C^  0.1409 ^Wild-type at 15°C^ | S3G Fig |
|  | *fat-2* | 25°C | Wild-type | 294.04±225.26 |  | S3G Fig |
|  |  | 15°C | Wild-type | 184.43±97.67 | 0.4825 | S3G Fig |
|  |  | 25°C | *mdt-15(tm2182)* | 227.79±167.90 | 0.7039 | S3G Fig |
|  |  | 15°C | *mdt-15(tm2182)* | 75.16±29.29 | 0.1704  0.1958 *^mdt-15(-)^* ^at 25°C^  0.1370 ^Wild-type at 15°C^ | S3G Fig |
|  | *fat-3* | 25°C | Wild-type | 117.35±43.19 |  | S3G Fig |
|  |  | 15°C | Wild-type | 106.85±15.54 | 0.7123 | S3G Fig |
|  |  | 25°C | *mdt-15(tm2182)* | 129.49±50.55 | 0.7675 | S3G Fig |
|  |  | 15°C | *mdt-15(tm2182)* | 120.05±27.02 | 0.9312  0.7896 *^mdt-15(-)^* ^at 25°C^  0.5039 ^Wild-type at 15°C^ | S3G Fig |
|  | *fat-4* | 25°C | Wild-type | 76.76±40.48 |  | S3G Fig |
|  |  | 15°C | Wild-type | 83.20±21.04 | 0.8191 | S3G Fig |
|  |  | 25°C | *mdt-15(tm2182)* | 104.79±67.96 | 0.5725 | S3G Fig |
|  |  | 15°C | *mdt-15(tm2182)* | 75.30±24.14 | 0.9599  0.5179 *^mdt-15(-)^* ^at 25°C^  0.6915 ^Wild-type at 15°C^ | S3G Fig |
|  | *fat-5* | 25°C | Wild-type | 40.37±11.57 |  | S3G Fig |
|  |  | 15°C | Wild-type | 41.38±3.34 | 0.8908 | S3G Fig |
|  |  | 25°C | *mdt-15(tm2182)* | 13.85±3.45 | 0.0190 | S3G Fig |
|  |  | 15°C | *mdt-15(tm2182)* | 0.29±0.12 | 0.0039  0.0024 *^mdt-15(-)^* ^at 25°C^  <0.0001 ^Wild-type at 15°C^ | S3G Fig |
|  | *fat-6* | 25°C | Wild-type | 146.57±116.25 |  | S3G Fig |
|  |  | 15°C | Wild-type | 123.87±62.41 | 0.7805 | S3G Fig |
|  |  | 25°C | *mdt-15(tm2182)* | 63.36±53.81 | 0.3235 | S3G Fig |
|  |  | 15°C | *mdt-15(tm2182)* | 36.90±12.05 | 0.1794  0.4527 *^mdt-15(-)^* ^at 25°C^  0.0768 ^Wild-type at 15°C^ | S3G Fig |
|  | *fat-7* | 25°C | Wild-type | 6.05±2.62 |  | S3G Fig |
|  |  | 15°C | Wild-type | 141.36±59.25 | 0.0168 | S3G Fig |
|  |  | 25°C | *mdt-15(tm2182)* | 0.01±0.02 | 0.0163 | S3G Fig |
|  |  | 15°C | *mdt-15(tm2182)* | 0.04±0.07 | 0.0166  0.5728 *^mdt-15(-)^* ^at 25°C^  0.0145 ^Wild-type at 15°C^ | S3G Fig |
| qRT-PCR  (relative mRNA levels) | *fat-7* | 15°C | Wild-type | 1.0±0.0 |  | S4A Fig |
|  |  | 15°C | *nhr-49(gk405)* | 0.00±0.00 | <0.0001 | S4A Fig |
| Oil red O intensity (arbitrary unit) |  | 25°C | Wild-type | 0.23±0.07 |  | S4C Fig |
|  |  | 15°C | Wild-type | 0.21±0.06 | 0.4123 | S4C Fig |
|  |  | 25°C | *nhr-49(gk405)* | 0.25±0.09 | 0.1841 | S4C Fig |
|  |  | 15°C | *nhr-49(gk405)* | 0.34±0.09 | <0.0001  0.0009 *^nhr-49(-)^* ^at 25°C^  <0.0001 ^Wild-type at 15°C^ | S4C Fig |
| qRT-PCR  (relative mRNA levels) | *hsp-6* | 15°C | Wild-type | 1.0±0.0 |  | S5A Fig |
|  |  | 15°C | *mdt-15(tm2182)* | 1.32±0.23 | 0.0065 | S5A Fig |
|  | *hsp-60* | 15°C | Wild-type | 1.0±0.0 |  | S5A Fig |
|  |  | 15°C | *mdt-15(tm2182)* | 1.17±0.49 | 0.4091 | S5A Fig |
|  | *Y22D7AL.10* | 15°C | Wild-type | 1.0±0.0 |  | S5A Fig |
|  |  | 15°C | *mdt-15(tm2182)* | 1.16±0.58 | 0.6510 | S5A Fig |
|  | *hsp-4* | 15°C | Wild-type | 1.0±0.0 |  | S5A Fig |
|  |  | 15°C | *mdt-15(tm2182)* | 1.34±0.13 | 0.0019 | S5A Fig |
|  | *enpl-1* | 15°C | Wild-type | 1.0±0.0 |  | S5A Fig |
|  |  | 15°C | *mdt-15(tm2182)* | 1.30±0.36 | 0.2235 | S5A Fig |
|  | *hsp-3* | 15°C | Wild-type | 1.0±0.0 |  | S5A Fig |
|  |  | 15°C | *mdt-15(tm2182)* | 1.83±0.20 | 0.0020 | S5A Fig |
| qRT-PCR  (relative mRNA levels) | *pas-4* | 25°C | Wild-type | 1.0±0.0 |  | S5B Fig |
|  |  | 15°C | Wild-type | 1.36±0.16 | 0.0037 | S5B Fig |
|  |  | 25°C | *mdt-15(tm2182)* | 0.93±0.25 | 0.5918 | S5B Fig |
|  |  | 15°C | *mdt-15(tm2182)* | 1.31±0.15 | 0.0073  0.0425 *^mdt-15(-)^* ^at 25°C^  0.6637 ^Wild-type at 15°C^ | S5B Fig |
|  | *pas-5* | 25°C | Wild-type | 1.0±0.0 |  | S5B Fig |
|  |  | 15°C | Wild-type | 1.23±0.33 | 0.2094 | S5B Fig |
|  |  | 25°C | *mdt-15(tm2182)* | 0.93±0.31 | 0.6682 | S5B Fig |
|  |  | 15°C | *mdt-15(tm2182)* | 1.56±0.21 | 0.0017  0.0146 *^mdt-15(-)^* ^at 25°C^  0.1471 ^Wild-type at 15°C^ | S5B Fig |
|  | *pbs-1* | 25°C | Wild-type | 1.0±0.0 |  | S5B Fig |
|  |  | 15°C | Wild-type | 1.09±0.18 | 0.3435 | S5B Fig |
|  |  | 25°C | *mdt-15(tm2182)* | 0.85±0.19 | 0.1581 | S5B Fig |
|  |  | 15°C | *mdt-15(tm2182)* | 1.00±0.14 | 0.9493  0.2321 *^mdt-15(-)^* ^at 25°C^  0.4709 ^Wild-type at 15°C^ | S5B Fig |
|  | *rpt-3* | 25°C | Wild-type | 1.0±0.0 |  | S5B Fig |
|  |  | 15°C | Wild-type | 1.16±0.14 | 0.0574 | S5B Fig |
|  |  | 25°C | *mdt-15(tm2182)* | 0.86±0.08 | 0.0148 | S5B Fig |
|  |  | 15°C | *mdt-15(tm2182)* | 1.20±0.15 | 0.0332  0.0066 *^mdt-15(-)^* ^at 25°C^  0.7235 ^Wild-type at 15°C^ | S5B Fig |
|  | *rpn-9* | 25°C | Wild-type | 1.0±0.0 |  | S5B Fig |
|  |  | 15°C | Wild-type | 0.99±0.50 | 0.9603 | S5B Fig |
|  |  | 25°C | *mdt-15(tm2182)* | 0.78±0.32 | 0.2249 | S5B Fig |
|  |  | 15°C | *mdt-15(tm2182)* | 1.07±0.36 | 0.6891  0.2674 *^mdt-15(-)^* ^at 25°C^  0.7837 ^Wild-type at 15°C^ | S5B Fig |
|  | *rpn-12* | 25°C | Wild-type | 1.0±0.0 |  | S5B Fig |
|  |  | 15°C | Wild-type | 1.14±0.18 | 0.1668 | S5B Fig |
|  |  | 25°C | *mdt-15(tm2182)* | 0.67±0.24 | 0.0357 | S5B Fig |
|  |  | 15°C | *mdt-15(tm2182)* | 1.28±0.19 | 0.0286  0.0081 *^mdt-15(-)^* ^at 25°C^  0.3377 ^Wild-type at 15°C^ | S5B Fig |
|  | *elb-1* | 25°C | Wild-type | 1.0±0.0 |  | S5B Fig |
|  |  | 15°C | Wild-type | 1.07±0.26 | 0.6029 | S5B Fig |
|  |  | 25°C | *mdt-15(tm2182)* | 0.90±0.18 | 0.3222 | S5B Fig |
|  |  | 15°C | *mdt-15(tm2182)* | 1.09±0.28 | 0.5452  0.3026 *^mdt-15(-)^* ^at 25°C^  0.9360 ^Wild-type at 15°C^ | S5B Fig |
|  | *otub-1* | 25°C | Wild-type | 1.0±0.0 |  | S5B Fig |
|  |  | 15°C | Wild-type | 1.26±0.25 | 0.0817 | S5B Fig |
|  |  | 25°C | *mdt-15(tm2182)* | 1.14±0.25 | 0.3116 | S5B Fig |
|  |  | 15°C | *mdt-15(tm2182)* | 1.49±0.25 | 0.0077  0.0971 *^mdt-15(-)^* ^at 25°C^  0.2396 ^Wild-type at 15°C^ | S5B Fig |
|  | *csn-5* | 25°C | Wild-type | 1.0±0.0 |  | S5B Fig |
|  |  | 15°C | Wild-type | 1.53±0.52 | 0.0877 | S5B Fig |
|  |  | 25°C | *mdt-15(tm2182)* | 1.19±0.40 | 0.3711 | S5B Fig |
|  |  | 15°C | *mdt-15(tm2182)* | 1.62±0.53 | 0.0570  0.2480 *^mdt-15(-)^* ^at 25°C^  0.8184 ^Wild-type at 15°C^ | S5B Fig |
| qRT-PCR  (relative mRNA levels) | *lgg-1* | 25°C | Wild-type | 1.0±0.0 |  | S5C Fig |
|  |  | 15°C | Wild-type | 0.82±0.25 | 0.1991 | S5C Fig |
|  |  | 25°C | *mdt-15(tm2182)* | 1.19±0.40 | 0.3735 | S5C Fig |
|  |  | 15°C | *mdt-15(tm2182)* | 1.26±0.46 | 0.2990  0.8310 *^mdt-15(-)^* ^at 25°C^  0.1437 ^Wild-type at 15°C^ | S5C Fig |
|  | *atg-18* | 25°C | Wild-type | 1.0±0.0 |  | S5C Fig |
|  |  | 15°C | Wild-type | 1.04±0.11 | 0.4926 | S5C Fig |
|  |  | 25°C | *mdt-15(tm2182)* | 1.28±0.14 | 0.0069 | S5C Fig |
|  |  | 15°C | *mdt-15(tm2182)* | 1.21±0.38 | 0.2992  0.7512 *^mdt-15(-)^* ^at 25°C^  0.4071 ^Wild-type at 15°C^ | S5C Fig |
|  | *bec-1* | 25°C | Wild-type | 1.0±0.0 |  | S5C Fig |
|  |  | 15°C | Wild-type | 0.84±0.24 | 0.2333 | S5C Fig |
|  |  | 25°C | *mdt-15(tm2182)* | 0.82±0.15 | 0.0510 | S5C Fig |
|  |  | 15°C | *mdt-15(tm2182)* | 1.16±0.30 | 0.3382  0.0916 *^mdt-15(-)^* ^at 25°C^  0.1527 ^Wild-type at 15°C^ | S5C Fig |
|  | *unc-51* | 25°C | Wild-type | 1.0±0.0 |  | S5C Fig |
|  |  | 15°C | Wild-type | 1.01±0.02 | 0.2017 | S5C Fig |
|  |  | 25°C | *mdt-15(tm2182)* | 1.03±0.15 | 0.7055 | S5C Fig |
|  |  | 15°C | *mdt-15(tm2182)* | 1.13±0.36 | 0.5043  0.6313 *^mdt-15(-)^* ^at 25°C^  0.5513 ^Wild-type at 15°C^ | S5C Fig |
|  | *atg-7* | 25°C | Wild-type | 1.0±0.0 |  | S5C Fig |
|  |  | 15°C | Wild-type | 0.87±0.19 | 0.2200 | S5C Fig |
|  |  | 25°C | *mdt-15(tm2182)* | 1.25±0.21 | 0.0498 | S5C Fig |
|  |  | 15°C | *mdt-15(tm2182)* | 0.86±0.16 | 0.1341  0.0241 *^mdt-15(-)^* ^at 25°C^  0.9191 ^Wild-type at 15°C^ | S5C Fig |
| *hsp-16.1::GFP* expression  (arbitrary units) |  | 15°C | Control RNAi | 0.12±0.04 |  | S6B Fig |
|  |  | 15°C+OA | Control RNAi | 0.09±0.04 | 0.0075 | S6B Fig |
|  |  | 15°C | *mdt-15* RNAi | 3.69±1.79 | <0.0001 | S6B Fig |
|  |  | 15°C+OA | *mdt-15* RNAi | 1.50±0.94 | <0.0001  <0.0001 *^mdt-15(tm2182)^* | S6B Fig |
| Body area (arbitrary unit) |  | 15°C | Wild-type | 154131±15329 |  | S6C Fig |
|  |  | 15°C+OA | Wild-type | 143386±18262 | 0.0270 | S6C Fig |
|  |  | 15°C | *mdt-15(tm2182)* | 102429±15159 | <0.0001 | S6C Fig |
|  |  | 15°C+OA | *mdt-15(tm2182)* | 118470±15667 | <0.0001  <0.0001 ^Wild-type at 15°C^  0.0007 *^mdt-15(tm2182)^* | S6C Fig |

Double-solid lines distinguish different assays and different targets are separated by solid lines. *p* values were calculated by using unpaired two sample two-tailed Student’s *t*-test. The *p* values that were calculated from specific conditions other than wild-type (the first raw of each experiment that is separated by solid line) are additionally described with superscripts.
