# Supplementary material for: MDT-15/MED15 permits longevity at low temperature via enhancing lipidostasis and proteostasis
Source: PLoS Biol. 2019 Aug 13;17(8):e3000415. doi: 10.1371/journal.pbio.3000415 (PMC6692015; doi:10.1371/journal.pbio.3000415)
Supplement: S8 Table — (DOCX) [file pbio.3000415.s014.docx]

**Supplementary materials and methods**

Following are primer sequences used in this study.

| **Target gene** | **Sequence (Forward)** | **Target gene** | **Sequence (Reverse)** |
| --- | --- | --- | --- |
| *ama-1*-F | TGGAACTCTGGAGTCACACC | *ama-1*-R | CATCCTCCTTCATTGAACGG |
| *fat-1*-F | TTCACCATGCTTTCACCAACCAC | *fat-1*-R | GTGTACACTGGGAACCATTTAAGCC |
| *fat-2*-F | AGTTTCTGGAGTTGCATGCGCTATC | *fat-2*-R | CAGCTTCGTAGACCTCAATATCCTC |
| *fat-3*-F | GGCAACAATTCGGATGGTTAACAC | *fat-3*-R | GATGAGTGTTATGCTTGTCCTTCC |
| *fat-4*-F | GGTCTTAACTATCAGATTGAGCACC | *fat-4*-R | CGGAATTGCTCAATTTCAAGCC |
| *fat-5*-F | GTTCCAGAGGAAGAACTACCTCCCC | *fat-5*-R | GGGTGAAGCAGTAACGGAAGAGGGC |
| *fat-6*-F | GCGCTGCTCACTATTTCGGATGG | *fat-6*-R | GTGGGAATGTGTGATGGAAGTTGTG |
| *fat-7*-F | CTGCACGTCGCCGCAGCCATTG | *fat-7*-R | GAGAGCAAATGAGAAGACGGCC |
| *hsp-16.1/11*-F | CTCATGAGAGATATGGCTCAG | *hsp-16.1/11*-R | CATTGTTAACAATCTCAGAAG |
| *hsp-16.41*-F | CATATTCTGATTCAAATGCTCTTGA | *hsp-16.41*-R | TCCAAGTTTTCGGTTCAACTCG |
| *hsp-16.48/49*-F | TCATGCTCCGTTCTCCATTTTCTGATTC | *hsp-16.48/49*-R | CTTCTTTGGAGCCTCAATTTGAAGTTTTCC |
| *F44E5.4*/*F44E5.5*-F | GAATGGAAAGGTTGAGATCCTC | *F44E5.4*/*F44E5.5*-R | CCAACCAATCTTTCCGTATCTG |
| *hsp-70*-F | GATGAAGTTGTCTTGGTTGG | *hsp-70*-R | CAGTTGAGGTCCTTCCCATTG |
| *hsp-6*-F | CTATGGGCCCAAAAGGAAGAAACGTG | *hsp-6*-R | GGGAATACACTTTTCCTTGAGCCTC |
| *hsp-60*-F | CTATGGGCCCAAAAGGAAGAAACGTG | *hsp-60*-R | GGATTTCGCGACGGTGACTCCGTCC |
| *hsp-3*-F | CTCGGAACCACCTACTCGTGT | *hsp-3*-R | GTGATACGGTTTCCTTGGTCG |
| *hsp-4*-F | TCAGAAACTTCGCCGTGAGGT | *hsp-4*-R | AGAGTGACTCGATCTCGATCT |
| *Y22D7AL.10*-F | CCTCAAGACCTTCAAGCCAC | *Y22D7AL.10*-R | CGGTGGCTTCAAGGACTTTTC |
| *enpl-1*-F | CGTTGGAAAGACACCTCATTC | *enpl-1*-R | ATTTGGAATGAAGGCTGAAACTG |
| *pas-4*-F | TGCAAACACGAAGCAGCGC | *pas-4*-R | GGTTTTGAAAAGACGTGGAGTTC |
| *pas-5*-F | GTCGACCAACATATTGGCGTC | *pas-5*-R | TTTTGCGGTTATAAGTGAACCAG |
| *pbs-1*-F | GAAGAAATCAGCACTGGAACC | *pbs-1*-R | CGGGAGGTGATGAACGAGC |
| *rpt-3*-F | CAGAAATATCTTGGAGAAGGACC | *rpt-3*-R | CGTTTCGTAGCAATCGCGTC |
| *rpn-9*-F | GAAGGCGTTGAGCAAGGATC | *rpn-9*-R | CGCCATTTGCATTATCTGAGG |
| *rpn-12*-F | CGACGATTACATCAATCAGATGC | *rpn-12*-R | CTGTTGGCAGCGAGCAGG |
| *elb-1*-F | CGGAATCACACGGGACCC | *elb-1*-R | GAAGCCGGCGTCAGAAAGC |
| *otub-1*-F | CTGATGATAACGGTCAGGCTG | *otub-1*-R | AAGTGGAACAGATTTTTGCTCATC |
| *csn-5*-F | CTTCTTCAAATCACAACTTGACG | *csn-5*-R | GAGGAATTCGACGTTTGAGAAG |
| *atg-7*-F | GTGAAGCAGAAAAGATCTGG | *atg-7*-R | CGATGATGAAGAATTGCGAC |
| *atg-18*-F | CCGAAGTCAGACACTAGTCG | *atg-18*-R | GGAACCGATTGGTTGCTTGC |
| *bec-1*-F | GTTCAGCATAGAAAACTAAC | *bec-1*-R | CCGAAATCCATTGATTTCAC |
| *lgg-1*-F | CAGAAGATGCTCTGTTCTTC | *lgg-1*-R | CTTCCTCGTGATGGTCCTGG |
| *unc-51*-F | CTATGCCGAATCCGACCTTC | *unc-51*-R | GATGGTGGAGCGGCGAACGC |
